# The Sequencing Bead Array (SBA), a Next-Generation Digital Suspension Array

**DOI:** 10.1371/journal.pone.0076696

**Published:** 2013-10-07

**Authors:** Michael S. Akhras, Erik Pettersson, Lisa Diamond, Magnus Unemo, Jennifer Okamoto, Ronald W. Davis, Nader Pourmand

**Affiliations:** 1 Stanford Genome Technology Center, Stanford University, Palo Alto, California, United States of America; 2 World Health Organization Collaborating Centre for Gonorrhoea and other Sexually Transmitted Infections, Swedish Reference Laboratory for Pathogenic Neisseria, Department of Laboratory Medicine, Microbiology, Örebro University Hospital, Örebro, Sweden; 3 Department of Bioengineering, Stanford University, Stanford, California, United States of America; 4 Howard Hughes Medical Institute, Stanford University, Stanford, California, United States of America; 5 Department of Biomolecular Engineering, University of California Santa Cruz, Santa Cruz, California, United States of America; Deutsches Krebsforschungszentrum, Germany

## Abstract

Here we describe the novel Sequencing Bead Array (SBA), a complete assay for molecular diagnostics and typing applications. SBA is a digital suspension array using Next-Generation Sequencing (NGS), to replace conventional optical readout platforms. The technology allows for reducing the number of instruments required in a laboratory setting, where the same NGS instrument could be employed from whole-genome and targeted sequencing to SBA broad-range biomarker detection and genotyping. As proof-of-concept, a model assay was designed that could distinguish ten Human Papillomavirus (HPV) genotypes associated with cervical cancer progression. SBA was used to genotype 20 cervical tumor samples and, when compared with amplicon pyrosequencing, was able to detect two additional co-infections due to increased sensitivity. We also introduce in-house software Sphix, enabling easy accessibility and interpretation of results. The technology offers a multi-parallel, rapid, robust, and scalable system that is readily adaptable for a multitude of microarray diagnostic and typing applications, e.g. genetic signatures, single nucleotide polymorphisms (SNPs), structural variations, and immunoassays. SBA has the potential to dramatically change the way we perform probe-based applications, and allow for a smooth transition towards the technology offered by genomic sequencing.

## Introduction

DNA microarray technology originally evolved from Southern blotting with the general principle of attaching DNA to a surface, and interrogating known DNA sequences. A major breakthrough was reported in 1995, with a miniaturized microarray designed for gene-expression profiling [1], which has since been formatted to a multitude of applications to expand our molecular toolbox [2]. Microarrays dominated the high-throughput genomic space until the advent of Next-Generation Sequencing (NGS) in 2005 [3,4]. With increased speed and a continuous drop in cost-per-base-sequenced, NGS may eventually replace the need for probe and hybridization based technologies, such as Real-Time PCR and microarrays [5-7]. Until then, there is still need for arrayed signatures and targeted sequencing, and with novel approaches the benefits of both systems can be combined, as in, for example, Array-Seq [8,9].

Suspension array technology presents an embodiment of microarray technology in which the typical spotted planar array is replaced with microspheres with distinct optical properties that can move freely in a solution [10,11]. Benefits include i) ease of use, ii) low cost, iii) statistical superiority in data acquisition, iv) rapid hybridization kinetics, v) improved specificity and sensitivity, vi) multiple biomolecule testing (e.g. DNA and proteins), and vii) simplified array preparation. However, a major limitation lies in the relatively low array sizes due to limited optical combinations of the spheres. The nanoparticle-based bio-barcode is an ultra-sensitive suspension array variant, where coated nanoparticles are selected for via targets, with subsequent release of bio-barcode nucleic acids for downstream processing [12].

The NGS Roche 454 Life Sciences technology [4] combined Pyrosequencing [13] with emulsion PCR [14] sample preparation of randomly generated DNA libraries for massively parallel sequencing applications. In brief, emulsion PCR traps an individual bead coated with a primer together with one unique DNA target containing the corresponding priming site. Via PCR, the sequence is cloned onto the bead in an emulsion with ideally only one template initially present. Each bead is immobilized onto a slide with picoliter sized wells and individually Pyrosequenced. Life Technologies Ion Torrent semiconductor sequencing [15,16] took a novel approach by replacing Pyrosequencing chemistry with a direct electrical detection of polymerase mediated nucleotide incorporation events [15-18]. This approach is highly scalable and allows for large-scale production, thus offering a low-cost alternative, using CMOS chips that include both the microwells and the biosensor.

**Figure 1:**
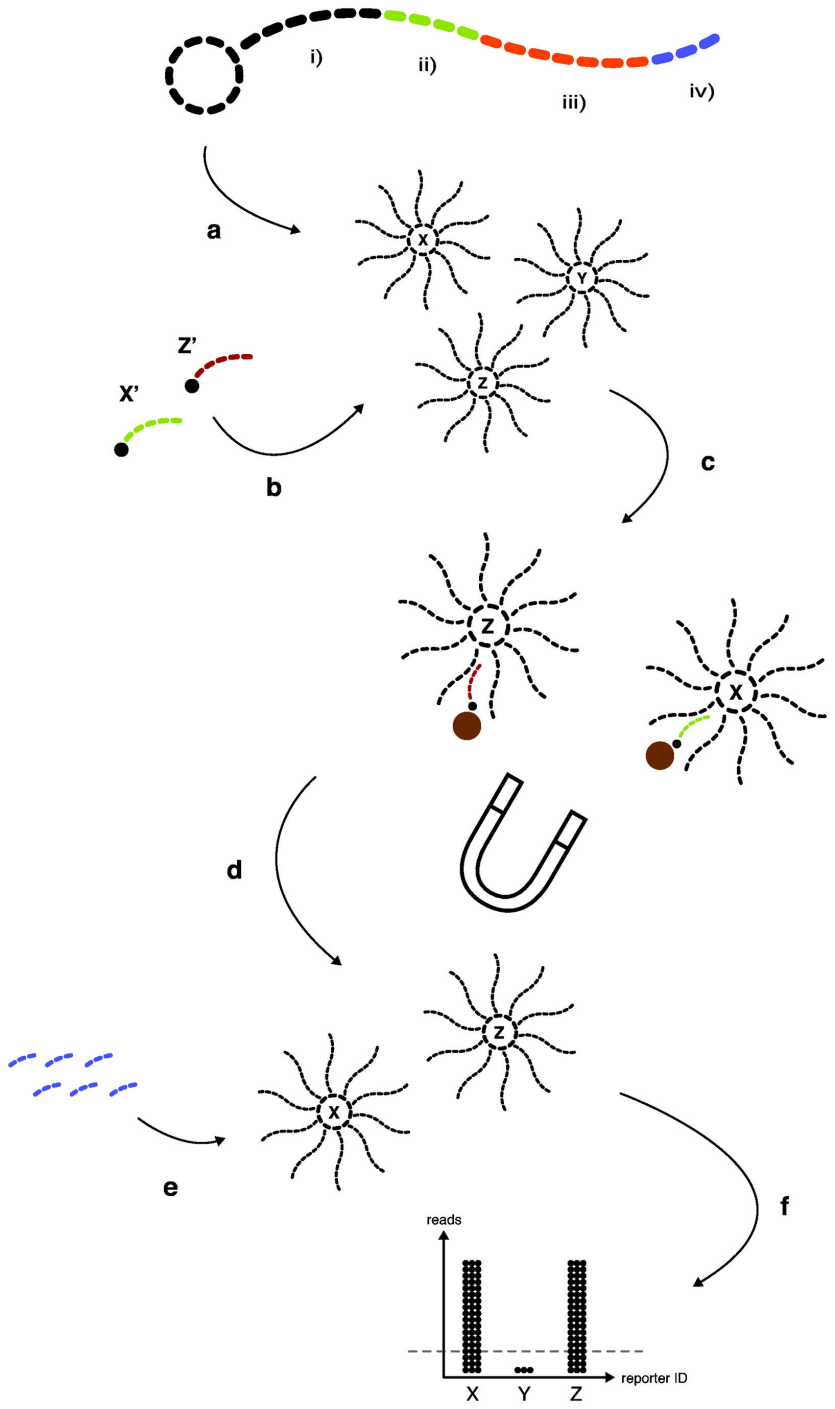
Schematic illustration of the Sequencing Bead Array (SBA) technology. The digital reporter selection workflow is comprised of target hybridization, reporter activation, immobilization, selection, priming and reporter calling. (**a**) A synthetic oligonucleotide viewed from the bead 5´-end consists of i) a general linker site for bead coupling (black), ii) a target hybridization site (green), iii) a target unique ID trace (orange), and iv) a general priming site (purple). A reporter consists of a multifold of identical clustered oligonucleotides on a bead. Each reporter is assigned a unique ID trace corresponding to specific biomarker target of interest (e.g. X). A library of reporters includes multiple reporter types, that each targets a specific biomarker (e.g. X, Y, Z). (**b**) Biotinylated target molecules X (green) and Z (dark red) (**c**) Hybridization of target molecules activate corresponding reporters by the addition of a biotin molecule. Activated reporters are immobilized and are magnetically selected via biotin coupling to streptavidin-coated paramagnetic beads (brown). (**d**) The number of reporters passing through the magnetic selection renders digital information of biomarker presence. (**e**) A sequencing primer complementary to the priming site is added at high concentrations to saturate all beads and, due to the reporter-oligonucleotide clusters, each selected reporter amplifies its own signal. Sequencing of the ID trace identifies all reporters in parallel. (**f**) Data is processed and reporters are called and sorted by Sphix software. Due to unspecific interactions between the reporters and the magnetic beads, some background noise (un-selected reporters) will survive the assay. Therefore a threshold line (dashed black line) is included to differentiate a positive from negative call. In this case X and Z were detectable above the threshold line.

Here we introduce the novel Sequencing Bead Array (SBA) technique (Figure 1) accompanied by in-house software solution, Sphix. SBA is a digital suspension array, where optical readout has been replaced with label-free NGS digital readouts of known sequence ID traces (i.e. bar codes). Furthermore, the reporter selection methodology in SBA takes a fundamentally new approach in which the targets select the synthetic reporters as digital indicators of the sample’s original content. SBA offers a i) highly scalable, ii) specific and sensitive, and iii) rapid alternative to microarray applications. In a proof-of-concept study, a model assay was developed for genotyping of Human Papillomavirus (HPV), formatted for the NGS Ion Personal Genome Machine (PGM). HPV is well known for the existence of multiple genotypes [19,20] and for the association of certain HPV infections with cervical cancer. Based on their oncogenic potential, HPV subtypes are classified as “high-risk” or “low-risk” HPVs. The developed model SBA assay targets ten high-risk HPV genotypes commonly associated with cervical cancer progression [21], and has been applied in this work to screen 20 cervical tumor samples (extracted genomic DNA). 

## Results and Discussion

### Data management and interpretation

A software tool Sphix, was developed to complement the Sequencing Bead Array (SBA) assay (Software S1). The Ion Torrent Personal Genome Machine (PGM) Torrent Suite pre-filters all sequencing data into three general categories: i) polyclonal filters, ii) low-quality filters and iii) primer-dimer (further discussed and defined in Table 1). All reads that pass thru these filters are then accessible in a FASTQ file format. Sphix handles the data processing of these files and provides algorithmic profiling of the sample. The resulting comma-separate value (CSV) files are easily integrated to other software application for further analysis and generation of graphical representation in portable document format (PDF), which could be further developed for a user-friendly experience on laptops or handheld devices (Figure S1). Sphix and training data sets (Torrent Suite reports and sequencing data files reported here) are all freely accessible (Software S1 and Data S1).

**Table 1:** Quality parameters for a 10-plex Human Papillomavirus (HPV) Sequencing Bead Array (SBA) assay.

	**a.**			**b.**			**c.**	
	**AVG**	**STDEV**		**AVG**	**STDEV**		**AVG**	**STDEV**
I. Reporter population	6.87x10^5^	±7.18%		1.46x10^4^	±29.67%		2.04x10^5^	±30.44%
II. Polyclonality	35.20%	±3.95%		19.65%	±7.16%		39.59%	±2.22%
III. Low quality	9.68%	±1.85%		58.47%	±14.61%		31.74%	±5.57%
IV. Uncalled reporters	3.07%	±0.22%		1.50%	±0.44%		1.57%	± 0.33%
V. Called reporters	52.04%	±2.68%		20.38%	±7.40%		27.10%	±3.89%
VI. Bead density	5.93x10^5^	±15.11%		N/A	N/A		N/A	N/A

Average (AVG) calls and standard deviation percentages (STDEV) for assay parameters. (a) Library variability of six identical individually constructed reporter libraries (metadata Table S2). (b) Background noise of six parallel negative (water) selections (metadata Table S3). (c) Six parallel quintuple HPV selections (metadata Table S4). The Ion Personal Genome Machine (PGM) Torrent Suite software automatically filters parameters I-III during a sequencing run, while IV and V are generated during Sphix analysis, and VI was quantitatively estimated via fluorescent readout data. I) Reporter population denotes the number of reporters that yield a strong enough sequencing read signal. II) Polyclonality denotes filtering of reporters with mixed sequence reads due to beads containing two or more different sequences, or loading of multiple beads in the same well (rare). III) Low quality denotes filtering of sequence reads with low signal quality and other abnormalities. To this category we have also included the smaller contributing primer-dimer filer (removes inserts < 8-bp), which has been merged for simplified interpretation. IV) Uncalled reporters denote obtained sequence reads that do not match any of the ID traces (caused by sequencing errors and alike). V) Called reporters denote obtained sequence reads that match ID traces. VI) Bead density denotes a quantitative estimate of the amount of beads/μl for a constructed reporter library. Bead density measurements were not performed in (**b** and **c**).

**Figure 2:**
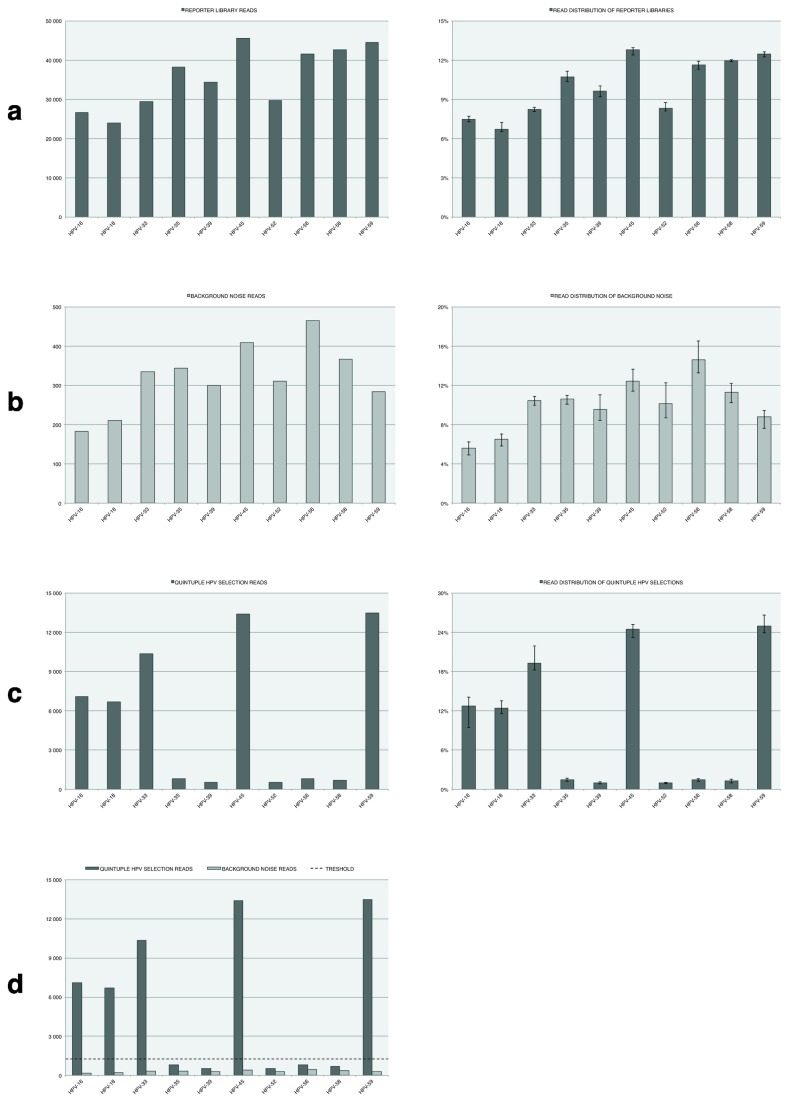
Reporter library construction and Sequencing Bead Array (SBA) assay performance. In (**a**-**c**), the left-hand side bar-histograms detail y-axis average read counts, while the right-hand side detail y-axis read distribution percentages amongst the ten reporters. Error-bars are also included in the right-hand side graphs to illustrate the read distribution variability amongst the replicates. The upper error-bar value is the highest observed frequency, while the lower error-bar value is the lowest observed frequency. (**a**) Results from six individually constructed reporter libraries sequenced side-by-side without any prior selections (metadata Table S2). (**b**) Results from six parallel negative (water) selections showing the background noise effects (metadata Table S3). (**c**) Results from six quintuple HPV selections of equimolar amounts of PCR-products derived from Human Papillomavirus (HPV) plasmids HPV-16, HPV-18, HPV-33, HPV-45, and HPV-59 (metadata Table S4). (**d**) Read count bar-histograms (**b** and **c**) superimposed with the experimentally established threshold value (dashed line) of ~1261.5 reads, which clearly distinguishes a positive call from the background noise.

Theoretically it would be sufficient to sequence the target hybridization region (Figure 1). However a 10-bp ID trace was included for a rapid readout process and to lay the groundwork for more complex future high-throughput applications (Table S1). Future Sphix algorithms designs could then compensate for differences in reporter distributions [22] (Figure 2), and use of error-correcting barcodes [23] to decrease sequencing errors. A multidimensional ID trace strategy [24] could also be used, i.e. where a specific panel of biomarkers are used with different patient specific sets of ID traces (e.g. patient A with biomarkers X, patient B with the same biomarkers X, etc.). This would lower the overall cost per sample by screening multiple patients in the same sequencing run. Data sorting and interpretation could be nearly instantaneous when using Sphix algorithms with different ID trace information.

### Reporter library construction

A reporter library is defined as an equimolar set of reporters with distinct target and ID traces (Figure 1). For compatibility with the PGM instrument, the reporters constituted of ~1-micron acrylamide beads with approximately 800,000 clustered oligonucleotides [15,16]. A preferred reporter library construction strategy would be to directly conjugate synthetic oligonucleotides to the beads, thus allowing for a high quality robust manufacturing setup. Then modified nucleic-acid oligonucleotides, e.g. locked nucleic acids (LNA) [25] and peptide nucleic acids (PNA) [25], could be used for improved target hybridization. Furthermore, direct conjugation would allow for use of oligonucleotides with the target hybridization site at the 3´-end, resulting in a variety of reporter activation modes (Figure S2). Potential added benefits could allow for increased sensitivity, specificity, and dynamic range, enabling single-base resolution (e.g. SNP-calling). Direct conjugation of other types of biomolecules (e.g. antibodies) to the beads could allow for flexibility in the choice of interrogated biomarker molecule (Figure S3).

However, since direct conjugation was not yet available with PGM compatible acrylamide beads. The proof-of-concept setup was based on clonal amplification of synthetic oligonucleotide templates via emulsion PCR. Emulsion PCR technology relies on an enzyme producing the DNA, with increased risk of beads with suboptimal DNA copy number, and other introduced artifacts. For added compatibility, the general priming site was placed at the 3´-end of all reporters (Figure 1). Three different template resources were investigated: i) a synthetic single-stranded DNA oligonucleotide with a linker sequence complementary to the attached bead primer (the positive strand), ii) its reverse-complement strand (the negative strand), and iii) complementary strands annealed to form a double-stranded DNA template. All template variants were able to generate reporter libraries containing the correct sequence composition; however the double-stranded templates were used for this assay to achieve a more even reporter distribution. The synthetic oligonucleotide templates used for emulsion PCR were, on average, ~115-bp in length (Table S1). These ultramers add complexity in terms of costs and inherent need for extensive purification. Variants with a shorter linker region (average size 85-bp) were also investigated and successfully implemented (data not shown), making the longer versions optional depending on library complexity and quality parameters desired. In-house oligonucleotide synthesis was employed without extensive purification protocols; while sufficient for an initial proof-of-concept, the lower quality grade accounts for variability. This phenomenon is clearly reflected in the library reporter read distribution (Figure 2), and thus the assay could be further optimized by more extensive purification of the oligonucleotide templates, and inclusion of internal normalization standards. Since the reporter oligonucleotide templates have many common regions (Table S1), one could employ a ligation assembly strategy of smaller oligonucleotides to increase oligonucleotide quality and lower production cost [26,27], or produce longer oligonucleotides via a PCR derived strategy [28].

A model 10-plex SBA assay was designed to genotype ten high-risk HPV genotypes (HPV-16, HPV-18, HPV-33, HPV-35, HPV-39, HPV-45, HPV-52, HPV-56, HPV-58, and HPV-59) commonly associated with cervical cancer progression [21]. For each targeted genotype, unique oligonucleotide templates with distinct target hybridization sites and a corresponding ID trace were used to generate libraries of reporters (Table S1). To investigate construction reproducibility, identical reporter libraries were generated from six separate emulsion PCRs and sequenced individually to examine library variability (Figure 2a, Table 1a, and Table S2). The emulsion PCR reaction generated approximately 100-µl of reporter library with an estimated average bead density of ~5.93 x10^5^ beads/μl. A fixed reporter library volume of 10-µl was used per SBA reaction (total ~5.93 x10^6^ beads). However the average reporter population in a sequencing run was only 6.87 x10^5^, resulting in a loading efficiency of only ~10% and, accordingly loss of resolution. In an ideal case for a 10-plex library, each reporter type would constitute 10% of all the called reporters. The observed average reporter distribution range was 6.63%-12.48%, attributable to differences in sequence composition, template concentrations, template quality, and handling. A variety of oligonucleotide template concentrations were explored; however, it proved difficult to lower the polyclonality factor, which is an inherent problem with the emulsion PCR method. Once produced, the library was refrigerated and stored for up to one month. Reporter libraries sequenced directly after production and those sequenced within a one-month time frame demonstrated little or no degradation in quality, which could be tracked via fluorescence measurements of the bead-clustered oligonucleotides and via sequencing runs (data not shown).

### SBA assay validation and performance

The SBA assay methodology was divided into two events; i) target hybridization to activate the reporters, and ii) selection of activated reporters. The SBA assay experimental settings were optimized with synthetic targets and PCR products derived from plasmids with cloned HPV DNA fragments (henceforth referred to as HPV plasmids). Target hybridization temperature profiles were explored ranging in temperature from 37-50°C in 5 minute to 24-hour protocols. Annealing times less than one hour resulted in reproducibility difficulties, and thus a 1-hour protocol was chosen, as little or no improvements were observed with longer protocols. A higher annealing temperature also resulted in lowered observed background noise. Reporter selection was explored using both a manual approach (with a handheld magnetic-rack), and an automated robotic system; the automated variant offered uniform reproducibility and ease-of-use, and was therefore used in all detailed experiments. Target-to-bead ratios were explored by combing a fixed amount of reporter library beads (~5.93 x10^6^) with 1-picomolar target molecules in variable reaction volumes of 5-40-µl, (equivalent of ~1.48 x10^5^ -1.19 x10^6^ beads/μl). The higher bead densities rendered high background signals, while a less dense reaction volume produced a stronger signal-to-noise ratio. Activated reporters clumping and falsely co-selecting unwanted neighboring reporters, and or general steric hindrance due to high concentration of beads might have caused this. However bead density calculations were based on fluorescent measurements and estimations (Table 1 and Table S2), and should not be considered absolute values. Also optical effects caused by the beads was not accounted for in the fluorescent measurements.

Background noise is caused by unspecific aggregation between reporters and magnetic selection beads. To confidently distinguish a positive call, a minimum detectable value is needed. A threshold was therefore experimentally determined by performing six replicate selections on a negative (pure water), processed with the same conditions as the genomic samples (Figure 2b, Table 1b, and Table S3). Next-Generation Sequencing (NGS) read count data follows a Poisson or negative binominal distribution (when overdispersion persists) [29]. When read counts are below ~100 (e.g. individual reporter read counts, Figure 2), even a small increase in counts reduces the impact of shot noise, while at higher counts shot noise effects are lower. However since the proof-of-concept SBA assay operates at higher read count a level, a simplifying assumption that the background read count noise follows a normal distribution was made. The statistical three-sigma rule (the mean + 3x the standard deviation) was applied to the background noise data set, to establish a minimum threshold that would account for variability. This assumption is only applicable for sufficient number of background reads (i.e. mean reporter read counts > 100 reads), and would need to be statistically adjusted for future SBA embodiments with lowered background noise. There is also an uneven distribution of reporter reads in the background noise. In theory, reporter specific thresholds could be used to account for differences. However, a conservative threshold value of ~1261.5 was used, based on the reporter for HPV-56 with the highest background read count. Henceforth: reporter reads > threshold = positive call, and reporter read counts ≤ threshold = background noise. The threshold is specific to this library set, and for other applications similar thresholds need to be established.

To determine a lower detection limit of the SBA assay, target-to-bead ratios were explored using a fixed bead density, while varying the amount of targets from 1-picomolar to 1-femtomolar molecules. Assay capability was investigated employing a two-target detection model in which two synthetic targets, T-45 and T-59 (corresponding to biomarker sequences HPV-45, and HPV-59, respectively) were present at various concentrations; T-45 was present at a fixed amount of 1-picomolar molecules, while T-59 ranged from 1-picomolar down to 1-femtomolar molecules through a ten-fold dilution series. A lower detection limit of 10-femtomolar was observed (data not shown). However with an estimated bead-loading efficiency of ~10%, only a subset of the reporters are actually read. This fraction is dependent on total reporter population in the set, and could be further optimized with improved loading schemes, and chips supporting more wells. Target-to-reporter ratio is essential and will affect the capabilities of the assay. Any given reporter requires a minimum finite number of targets in order to be activated and selected. Targets in abundance will saturate the reporters, which could allow for improved ranges by adjusting amounts of total reporters used in the assay.

PCR products derived from HPV plasmids of the ten targeted HPV genotypes were individually detected with the SBA assay, and results were validated by amplicon Pyrosequencing [26,30]. Patients may carry multiple HPV infections and, though rare, have been shown to carry as many as five genotypes [30]. Therefore, the SBA assay was further deployed to screen pools of PCR products derived from different HPV plasmids. Equimolar pooling of PCR products from the five high-risk genotypes HPV-16, HPV-18, HPV-33, HPV-45, and HPV-59 (1/5 component each) was used as a target source to simulate a quintuple infection. The experiment was repeated six times to also investigate reproducibility of the assay, and in all cases all five HPV subtypes were successfully detected above the threshold (Figure 2c and d, Table 1c, and Table S4).

Differences in i) oligonucleotide template quality, ii) emulsion PCR construct yield, iii) sample source quality, iv) chip-loading, v) batches of kits and chips, and vi) handling, contributed to library variability between sequencing runs (Table 1 and Tables S2-S4). Reporter selections slightly increase polyclonal and significantly increase the low quality filtering, while the percentage of uncalled reads decrease (Table 1c). This phenomenon is also true for the background noise profile with significantly higher degree of low-quality filtering albeit lowered polyclonal filtering (Table 1b). Selection of beads with a multitude of target hybridization match and mismatch sites, may cause the increase in polyclonal filtering. In both cases (Table 1b and c) the total amount of beads to be loaded on the sequencing chip is lower after the selection when comparing with sequencing of a native reporter library (Table 1a). This also results in higher total read-count variability, with an increase in the standard deviation of the reporter population. This might further favor certain background noise effect with sup-optimal beads interfering with the process. However since the PGM Torrent Suite filtering algorithms is company proprietary knowledge, it is difficult to further speculate on the causal effects. It could be advantageous for future work to alter or completed remove these filters as a main PGM screening objective is to ensure long high-quality reads, while the SBA assay requires significantly lower read-lengths with less emphasis on read quality, which could be further compensated by use of error-correcting barcodes [23]. When the bar-histograms are viewed with y-axis read distributions of called reporters percentages (reporter read count/called reporters) the profile variation is significantly lower (Figure 2a-c right-hand graphs), which indicates potential for a very robust assay once handling can be automated. Optimizing target-to-bead ratio, reaction salt conditions and exploring alternative activation strategies (Figures S2 and S3), could further enhance overall assay performance and utility. Future improvements in sequencing sensors could allow for nanoparticle-sized reporters with lowered demand on numbers of clustered oligonucleotides. Such improvements would greatly increase target surface-area display and possibly target binding capacities, thus allowing for sensitivity near the nanoparticle bio-barcode assay attomolar range [12], thus circumventing need for prior nucleic acid amplification.

The Ion PGM Ion 314 chip was used throughout this work to achieve cost benefits. For situations that call for expanded reporter libraries, such as parallel sample interrogations, and increased dynamic range, sequencing chips with extended features should be explored. At present, the Ion PGM [15,16] is compatible with the Ion 314 chip having ~1.2 M wells, the Ion 316 chip having ~6.3 M wells, and the Ion 318 chip having ~11.3 M wells. The Ion Proton [15,16] uses sub-micron beads, allowing for decreased well-size and an even higher throughput with the PI chip having ~165 M wells, the PII chip having ~660 M wells, and the upcoming PIII chip having ~1.2 B wells (http://www.lifetechnologies.com. Accessed 2013 Aug 24).

### Genotyping of clinical samples

**Figure 3:**
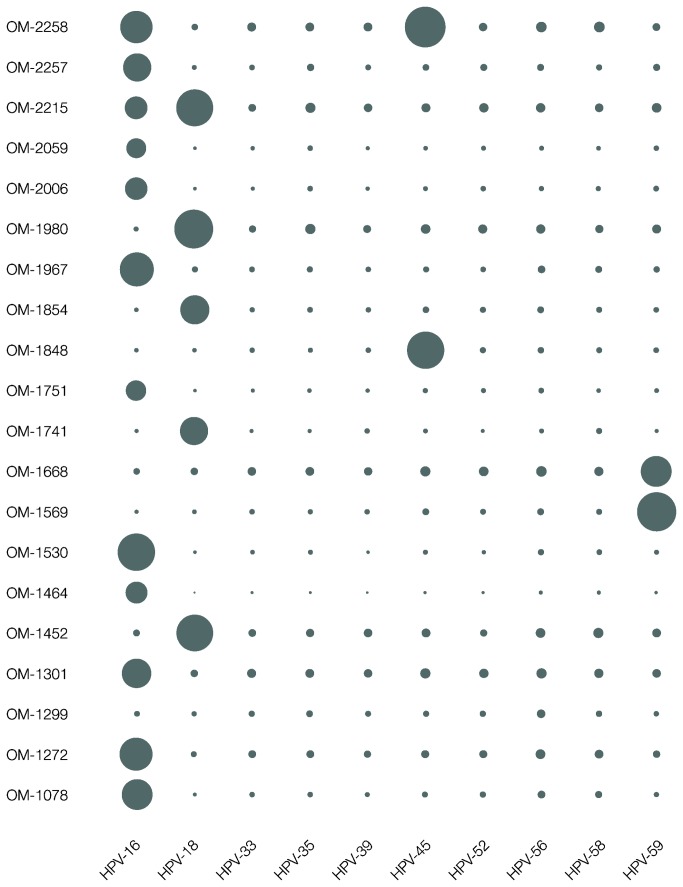
Sequencing Bead Array (SBA) assay screens of 20 cervical tumor samples. Bubble chart representation of the SBA assay screens of 20 cervical tumor samples (extracted genomic DNA). The data is based on number of called reporters (metadata in Figure S1 and Table S5). Results were called as follows: ten single infections for Human Papillomavirus (HPV) genotype HPV-16, four single infections for HPV-18, two single infections for HPV-59, one single infection for HPV-45, one dual co-infection for HPV-16, and HPV-18, one dual co-infection for HPV-16, and HPV-45, and one genotype negative sample. OM-1299 was negative as no reporter read count was above the background noise threshold.

Our main objective was to screen samples with unknown viral loads and profiles using PCR products derived from authentic clinical samples. Standardized nested PCR using general HPV primer sets PGMY09/11 and GP5+/6+ [31] was performed on 20 cervical tumor samples (extracted genomic DNA). As a comparator, amplicons were directly sequenced by Pyrosequencing [26,30]. The presence of HPV was confirmed in 19 of the 20 samples, and genotype results achieved by the two methods were in agreement, with the exception for samples OM-2215 and OM-2258 (Figure 3, Figure S1 and, and Table S5). A co-infection of HPV-16 and HPV-18 in OM-2215, and a co-infection of HPV-16 and HPV-45 in OM-2258 were detected with the SBA assay, whereas amplicon Pyrosequencing only detected the dominant genotype in each sample (HPV-18 and HPV-45 respectively). This was due to a higher sensitivity and dynamic range for the SBA assay. To confirm these findings, separate PCR screens were performed employing genotype-specific primers for HPV-16, HPV-18 and HPV-45, which in all cases generated the correct products with accurate sequence information. For sample OM-1299, neither method was able to detect any HPV genotype. Therefore ten additional separate PCR reactions were made using all ten possible genotype-specific primers [26,30], which did not either result in any genotype match rendering it a true negative with current assay conditions. Among the remaining 17 samples, ten were positive for HPV-16, four for HPV-18, one for HPV-45, and two for HPV-59.

The general nested primer set PGMY09/11 and GP5+/6+ [31], is described capable of amplifying a spectrum of approximately 30 different genotypes. Future embodiments of the HPV genotyping SBA assay will aim to explore multiple amplification regions and expand the SBA reporter set to cover the vast majority of classified HPV genotypes [19,20], as the true strength of the technology lies in its multiplexing capabilities. Since only a limited set of HPV genotypes were interrogated using the SBA assay, the negative result for OM-1299 may be due to the presence of an un-interrogated HPV genotype. Other possible reasons for why the sample was negative despite its tumor origin may be; i) a true negative that does not carry anymore HPV virus due to prior cleared infections, ii) the genomic DNA extract did not captured any HPV DNA, iii) the HPV DNA was integrated into the host genome without the incorporation of L1 gene for which the PCR targets, vi) the PCR primer sets might have been ineffective to capture the HPV DNA due to genetic sequence variations or similar, or v) it may also reflect that the OM-1299 case (http://www.oncomatrix.com. Accessed 2013 Aug 24) was derived from an invasive adenocarcinoma, of which some types do not contain any HPV DNA [32]. 

## Conclusion

The SBA method offers a unique and scalable assay for a multitude of molecular diagnostic and genotyping applications, where rapidness, sensitivity, specificity, and robustness are desired. The Proof-of-Concept study employs an emulsion PCR strategy to produce the beads, with its well-characterized challenges. For future versions of the technology a preferred reporter library manufacturing strategy would allow for direct conjugation of the synthetic oligonucleotides to an NGS platform compatible beads. The technology could be applied to a multitude of applications where conventional microarrays might otherwise be used, including: i) pathogen diagnostics, resistance detection and genotyping ii) single-nucleotide polymorphism (SNP) calling, iii) gene expression profiling, iv) miRNA detection, v) comparative genomic hybridization (CGH), vi) chromatin immunoprecipitation on chip (ChIP), vii) DNA adenine methyltransferase (DamID), viii) DNA barcode screenings, ix) alternative splicing (including fusion genes), and x) short tandem repeats (STRs) typing and xi) tilling arrays [2,22,33,34]. The current configuration is compatible with the 454 Life Sciences instruments GS FLX+, and GS Junior (http://www.454.com. Accessed 2013 Aug 24), the Life Technologies SOLID platform (htttp://www. lifetechnologies.com. Accessed 2013 Aug 24), and other upcoming bead-based technologies such as Fluorogenic Pyrosequencing [35]. Although the downstream readout instrumentation for the reporters could be of another nature (flow cytometer, simple flat-array or TaqMan probe-based optical readout), NGS offers unprecedented, massively parallel performance.

Furthermore, the proof-of-concept shows promise for even higher sensitivity and dynamic range, ultimately aiming for a PCR-free detection scheme. With future advancements in NGS technology, we envision low-cost alternative handheld instruments that are optimized for ID trace readout of reporters with fewer demands on sequencing performance (e.g. read-lengths, and demanding sequence combinations). This would allow for point-of-care diagnostics, desperately needed for many pathogens. SBA has the potential to dramatically change the way we perform probe-based applications, and allow for a smooth transition towards the technology offered by genomic sequencing. 

## Material and Methods

### Data management and interpretation

The software tool Sphix, was written in PERL (http://www.perl.org. Accessed 2013 Aug 24). Experimental data generated by the Ion Personal Genome Machine (PGM) software, the Torrent Suite 3.42 (Life Technologies, Carlsbad, CA), was manually retrieved from the Torrent Server, including FASTQ sequence data and sequencing run metrics of importance. Sphix performs barcode de-multiplexing by matching a given set of 10-bp ID traces with the sequence at the very beginning (5'-end) of each read, followed by binning and digital quantification of reporters. Only perfect matches were scored, counted and added to the results matrix. Based on the results, a CSV file is generated from which graphical bar-histogram representation can be generated using various third party software applications (e.g. Microsoft Excel), revealing the genotype profile of the sample. Sphix and training data sets are available online (Software S1 and Data S1).

The PGM dispenses a predefined custom flow order of nucleotides, wherein 4-flows make one cycle. The sequencing workflow requires that an initial 4-bp key sequence is used for read indexing for filtering and quality assessments. A theoretical minimum PGM dispensation order to cover the 4-bp key followed by a 10-bp ID trace from the predefined set of 153 ID traces (Table S1), was determined to be 10 cycles (i.e. 40 flows), corresponding to an estimated sequencing run time of 10 minutes. However, to cover reads that were shorter than expected, we chose to use 100 flows, corresponding to a sequencing runtime of ~37 minutes.

### Reporter library construction

A 10-plex HPV reporter library was constructed for multiplex genotyping of PCR products derived from the nested PCR product of general PCR primer-sets PGMY09/11 and GP5+/6+ [31]. The PCR generates an ~150-bp amplicon containing genotype specific sequence variations within the region. By aligning all the possible combinations, genotype unique sites of 20-25-bp could be found within the primer-flanking region. The GenBank sequences for respective HPV genotype (HPV-16; HQ644299, HPV-18; GQ180792, HPV-33; HQ537707, HPV-35; JX129488, HPV-39; M62849, HPV-45; EF202156, HPV-52; HQ537739, HPV-56; EF177181, HPV-58; HQ537768, and HPV-59; EU918767) were aligned to find genotype-specific hybridization candidate sites. Multiple candidates were chosen for each of the ten interrogated genotypes. The following design criteria was setup to filter the candidates: i) 20-25-bp of length, ii) melting temperatures (T_m_) of 52°C < Tm < 61°C, and iii) GC contents of 45% < GC < 60%. Sequences with hairpins or more than 5 single repeat were also eliminated. The online software application OligoAnalyzer 3.1 (Integrated DNA Technologies, Coralville, IA) was used to calculate all the criteria parameters. To ensure relative uniqueness, all sequences were compared to one another using megaBLAST (v 2.2.10) to eliminate any with an 80% match in 20 bases. The alignment and filtering resulted in one unique hybridization site for each of the ten interrogated HPV genotypes (Table S1).

The SBA oligonucleotides [100 µM] were synthesized in house (Stanford Genome Technology Center, Palo Alto, CA), and subsequently pooled with corresponding reverse complement strand in 1X STE-buffer [10 mM Tris (pH 7.6), 50 mM NaCl, and 0.1 mM EDTA]. Duplex templates were annealed in the GeneAmp PCR system 9700 (Life Technologies, Carlsbad, CA) at 95°C for 2 minutes followed by slow cooling until room temperature was reached. Following the hybridization step, all template duplexes were pooled at equimolar concentrations, and further diluted to 32 pM with ultraPURE H_2_O (Life Technologies, Carlsbad, CA). The diluted pool later served as the reporter library template for emulsion PCR. The emulsion PCR was performed using the Ion One Touch DL System with the Ion OneTouch 200 Template Kit v2 DL (Life Technologies, Carlsbad, CA), using ~2-micron acrylamide beads [15,16], in order to be compatible with the Ion PGM instrument (Life Technologies, Carlsbad, CA). The automated Ion OneTouch ES (Life Technologies, Carlsbad, CA) emulsion PCR library enrichment and cleanup procedure yields approximately 100-µl of reporter library stock in 1X Wash/Storage Buffer [PBS, supplemented with 0.2% Tween-20 (pH 7.7)].

For each SBA assay reaction, a fixed amount of 10-µl of reporter library was used. Bead density estimates of the constructed reporter libraries, were based on fluorescently based quantitative measurements using a single-stranded DNA assay on the Qubit 2.0 Fluorometer (Life Technologies, Carlsbad, CA). Each constructed reporter library was measured in three replicates of 3-µl, with three independent measurements. Furthermore the fluorescent measurements provided means for tracking degradation of the bead-clustered oligonucleotides for storage purposes, which was notable post one-month time frame. Quality control was also performed by sequencing the native reporter libraries with the Ion PGM instrument when using the Ion 314 chips with the Ion PGM 200 Sequencing Kit (Life Technologies, Carlsbad, CA) according to the manufacturer’s instructions.

### SBA assay validation and performance

Cloned HPV-plasmids for HPV-16, HPV-18, and HPV-45 were kindly provided by Dr. E.M. de Villiers (DKFZ, Heidelberg, Germany); HPV-33 and HPV-39 from Dr. M. Favre (Institute Pasteur, Paris, France); HPV-35 and HPV-56 DNA from Dr. A. Lorincz (Digene Corporation, Gaithersburg, MD, USA), HPV-52 DNA from Dr. W. Lancaster (Wayne State University School of Medicine, Detroit, MI, USA), and HPV-58 and HPV-59 DNA from Dr. T. Matsukura (National Institute of Health, Tokyo, Japan). The HPV plasmid concentrations were normalized at 100-ng/ml using an ND-100 Spectrophotometer (Thermo, Fisher Scientific, Waltham, MA). PCR amplification was performed using the nested PCR with general primers PGMY09/11 and GP5+/6+ [31] according to a previously described protocol [36], with an initial plasmid DNA concentration of 100-ng/ml, or ultraPURE H_2_O for background noise selections (Figure 2b, Table 1b, and Table S3). The GP6+ primer carried a 5'-end biotin molecule for cleaning and selection purposes. A magnetic workstation, the Magnatrix 1200 (NorDiag, Biotrin International Ltd., Dublin, Ireland) was employed for automating the lab work. Running custom-made scripts, the robot generated single-stranded templates of the PCR amplicons. M-270 beads (Life Technologies, Carlsbad, CA) were used for immobilizing the DNA followed by magnetic collection and washing, initially in 1X TE-buffer [10 mM Tris–HCl (pH 7.5), 0.1 mM EDTA] followed by thorough washes with purified H_2_O [18.2 MΩ.cm at 25 °C) (EMD Millipore, Billerica, MA] and NaOH [0.1 M] with constant mixing. Finally, the single-stranded products were released by heating to 80°C for 1 second in purified H_2_O [37].

The SBA assay was executed by i) target hybridization to activate and immobilize the reporters, and ii) selection of activated reporters. A 10-µl aliquot of merged reporter library stock was further diluted to a total of 30-µl in 1X Annealing Buffer [PBS, supplemented with 0.2% Tween-20 (pH 7.7)]. Target DNA was added in 10-µl aliquots (using only 10-µl of the 50-µl PCR products) in 1X Annealing Buffer to make the total reaction volume to 40-µl. The mixture was sonicated at maximum intensity for 10 seconds using the desktop water bath and sonicator B2500A-DTH (VWR, Radnor, PA). Sonication ensures maximum mixture and target exposure. Target hybridization was accomplished using an initial heating at 95°C for 2 minutes with a GeneAmp PCR system 9700, then immediately relocated to a Thermomixer R & MTP Microblock (Eppendorf, Hamburg, Germany) programmed at 70°C, followed by slow cooling to 45°C with constant shaking at 1300 rpm to ensure the reporters avoided sedimentation for maximum target exposure. The activated reporters were then incubated at room temperature for 5 minutes with continuous mixing with 13-µl streptavidin coated paramagnetic MyOne C1-beads (Life Technologies, Carlsbad, CA) in 1X Annealing Buffer Magnetic selection was performed manually with 1X Wash/Storage Buffer and 1X Elution buffer [0.1 M NaOH supplemented with 0.1% Tween-20] while using a Dynal Invitrogen bead separation magnetic rack (Life Technologies, Carlsbad, CA). The final product was further washed and concentrated with 1X Annealing Buffer to yield a total volume of 3-µl ready for sequencing primer annealing and sequencing readout with Ion PGM instrument using the Ion 314 chips with the Ion PGM 200 Sequencing Kit according to manufacturer instructions. The Sequencing runs were programmed at a constant of 100 flows for all reactions to ensure full-length ID traces. In addition to the manual magnetic selection process, the process was also compatible with the Ion OneTouch ES protocol (Life Technologies, Carlsbad, CA).

### Genotyping of clinical samples

The 20 cervical tumor samples (extracted genomic DNA) were obtained commercially (Oncomatrix, Inc., San Marcos, CA) from different females with accession codes OM-1078, OM-1272, OM-1299, OM-1301, OM-1452, OM-1464, OM-1530, OM-1569, OM-1668, OM-1741, OM-1751, OM-1848, OM-1854, OM-1967, OM-1980, OM-2006, OM-2059, OM-2215, OM-2257, and OM-2258. The samples were PCR amplified using a nested PCR with general primers PGMY09/11 and GP5+/6+ [31,36], with an initial sample concentration of 40-ng/ml genomic DNA, which yielded 50-µl PCR product. The full 50-µl PCR sample volume was used for all OM-samples, except: OM-1301, OM-1569, OM-1668, OM-1751, OM-1848, and OM-1854, for which only 10-µl PCR sample volume was used due to initial high DNA target concentration. The Magnatrix 1200 was programmed to generate single-stranded templates of the PCR amplicons.

In the cases of OM-2215 and OM-2258, amplicon Pyrosequencing [26,30] detected only a single (dominant) genotype, while the SBA detected two genotypes in each sample (HPV-16 and HPV-18 in OM-2215, and HPV-16 and HPV-45 in OM-2258). To confirm these findings, secondary PCR amplifications were performed using the genotype specific Multiple Sequencing Primer (MSP) MSP-16, MSP-18 and MSP-45 in separate reactions together with GP6+ [30], followed by Pyrosequencing using MSP-16, MSP-18 and MSP-45 as sequencing primers. In the case of OM-1299, where none of the methods manage to confirm a genotype, secondary PCR amplifications were performed using all the ten included genotype specific Multiple Sequencing Primer (MSP) MSP-16, MSP-18, MSP-33, MSP-35, MSP-39, MSP-45, MSP-52, MSP-56, MSP-58, and MSP-59 in separate reactions together with GP6+ [30], followed by Pyrosequencing using the included MSP sequencing primers individually. 

## Supporting Information

Data S1 **Raw sequencing data files for presented experiments.** The compressed file contains a table of content for all files included (0. TOC Supporting Data Files.txt) and raw sequencing data (PGM Torrent Suite run reports, FASTQ files and Sphix generated CSV files) for experiments presented in Figure 2 and Figure 3 (same data set as Figure S1).
(ZIP)

Figure S1 **Bar-histograms for 20 cervical tumor samples (extracted genomic DNA).** The reporter bars in the bar-histograms are represented with y-axis read counts (metadata in Table S5). Positive genotype calls reach beyond the included threshold (dashed line), while calls below are within background noise variation. The y-axis is scaled to a maximum of 15,000 reads in all of the graphs to allow for side-by-side comparison. Co-infections were observed in samples OM-2215 and OM-2258. OM-1299 was negative for all ten investigated HPV genotypes, i.e. no peak signal was distinguishable from background noise. Metadata for each sample-sequencing run is also included (upper right corners) for reporter population, polyclonality, low quality, uncalled reporters, and called reporters (defined in Table 1). Results were called as: ten single infections for HPV-16, four single infections for HPV-18, two single infections for HPV-59, one single infection for HPV-45, one dual co-infection for HPV-16, and HPV-18, one dual co-infection for HPV-16, and HPV-45, and one genotype-negative sample.
(PDF)

Figure S2 **Conceptual illustration of alternative reporter activation strategies.** A reporter library construction method in which synthetic oligonucleotides are directly coupled to the beads would allow for alternative reporter activation strategies. In contrast to the scheme outlined in Figure 1, the proposed cluster of synthetic oligonucleotides viewed from the 5´-end would instead consist of: i) a general linker site for bead coupling (black), ii) a target unique ID trace (orange), iii) a general priming site (purple), and iv) a target hybridization site (green). Biotin molecules are depicted as solid black dots that will bind to a streptavidin coated solid surface, here depicted as a paramagnetic bead (brown), for downstream separation. (**a**) Selectively hybridizing a complementary target (or tag) to a bead-clustered oligonucleotide at the 3´-end allows for a polymerization reaction using biotinylated nucleotides. This creates covalently bonded biotin molecules and thus possibly enables a stronger selection, and furthermore allows for single-base interrogations, e.g. single nucleotide polymorphisms (SNPs) detection. If the target template is longer (blue) the polymerization could continue further generating a longer complementary strand (red) with multiple biotin reporter activating molecules (black dots), given that a fraction of the dNTPs in the reaction are biotinylated. (**b**) Selectively hybridizing a biotinylated complementary target (or tag) to a bead-clustered oligonucleotide at the 3´-end could possibly ease steric hindrance. Also, by including multiple target hybridization sites (replicates or different targets), the process can be made more sensitive, with the possibility of binding multiple-targets (here represented as two duplicates). This strategy could be combined with a polymerase extension of the bead-clustered oligonucleotide at the 3´-end, generating a complementary strand (red), thus enabling a stronger selection basis. (**c**) Selectively hybridizing a complementary target (or tag) indirectly via a biotinylated capture probe (green) to a bead-clustered oligonucleotide at the 3´-end. This approach could be further combined with ligation or a gap-fill reaction (red) joining the fragments covalently via a polymerase and/or ligase-enzyme. This requires the capture probe to be 5´-phosphorylated, similar to that of the padlock probe design [38,39]. Combining a single-base specific polymerase extension of the 3´-end (or an complete extended gap-fill) with ligation to the biotinylated probe would allow for further interrogation of the bases in the juxtaposition of the ends, e.g. SNP calling [24,26,40]. (**d**) An initial polymerization reaction constructing a complementary sequence (red) to the target template (blue) could be introduced prior to activation. Activation could then be achieved by targeting a known downstream region of the cloned target (green), through hybridization or any of the strategies outlined above (**a**-**c**). Optionally in (**b**, **c** and **d**) the biotin could be directly conjugated to the magnetic bead.
(PDF)

Figure S3 **Conceptual illustration of immunoassay reporter activation strategies.** A reporter library construction method in which synthetic oligonucleotides and other biomolecules (e.g. antibodies) are directly coupled to the beads. This would allow for alternative reporter activation strategies, and targeting other biomolecules than nucleic acids. Biotin molecules are depicted as solid black dots that will bind to a streptavidin coated solid surface, here depicted as a paramagnetic bead (brown), for downstream separation. (**a**) A cluster of synthetic oligonucleotides viewed from the 5´-end consisting of: i) a general linker site for bead coupling (black), ii) a target unique ID trace (orange), iii) a general priming site (purple), and iv) a target hybridization site (green). The oligonucleotides could also be oriented as described in Figure 1. In contrast to previous versions the oligonucleotide-target or tag is conjugated to antibody-reporter, as used in e.g. the proximity ligation assay (PLA) for immunoassays [39,41-43]. Reporter activation in this case can be any of the previously described strategies (Figure 1 and Figure S2). (**b**) A reporter containing both bead-clustered oligonucleotides for signal amplification, and bead-conjugated antibodies (or other biomarkers) (green) for immunoassay target reporter activation and selection. The clustered oligonucleotides would in this case contain i) a general linker site for bead coupling (black), ii) a target unique ID trace (orange), and iii) a general priming site (purple). The reporters are activated and selected via an antibody-antigen-antibody complex (antigen in blue). A combinatorial assay targeting both genomic and proteomic markers could thus be envisioned [44].
(PDF)

Software S1 **Sphix software.** The compressed file contains an instruction file (readMe.txt) and the Sphix PERL script (sphix_v_01.pl) that was used for sorting and analyzing the data presented in this work.
(ZIP)

Table S1 **The Sequencing Bead Array (SBA) reporter oligonucleotide templates.** Oligonucleotide templates for emulsion PCR reporter library construction. All oligonucleotides are presented in the 5´ to 3´ direction. The upper half of the table represents the oligonucleotides used for the described 10-plex HPV SBA assay, while the lower half details a list of 153 Multiplex Identifiers (MIDs) which could be used as ID traces for future SBA assay embodiments. The upper half table (white) is compartmentalized into five different sections. Sequence follows assembly of compartments, i.e. 5´-Priming site -Key - ID trace X -Target hybridization site X - Linker site -3´. Each oligonucleotide was synthesized as both the strand displayed in the table, and its reverse complementary strand to form duplex DNA templates. The priming site decodes where the sequence primer attaches. The Key is for PGM decoding and quality control purposes during the sequencing run. The ID trace is unique to each reporter and biomarker target. The target hybridization sites correspond to a genotype-specific sequence present in the L1 gene of the HPV-genome. The linker was used to clone the fragments onto the beads during the emulsion PCR step. The lower half table (grey) details a pre-defined library of 153 Multiplex Identifiers (MIDs) from 454 sequencing GS FLX Titanium chemistry, which provides an excellent resource for future ID traces (Roche Diagnostics 2009. Using multiplex identifier (MID) adaptors for the GS FLX titanium chemistry–extended MID set. Technical Bulletin: Genome Sequencer FLX System. TCB no. 005-2009. Roche, Branchburg, NJ.).
(PDF)

Table S2 **Metadata for library variability studies of six individually emulsion PCR constructed 10-plex HPV reporter libraries (L1-L6).** The table contains Torrent Suite and Sphix-generated data from the sequencing runs performed on the interrogated libraries. Calls denote actual sequence read counts. Left columns contain sequence run information with reporter population and subcategories (defined in Table 1). Frequency denotes the fraction of the subcategories compared to the reporter population. Middle columns contain sequence read counts for reporter distribution, and frequency denotes the fractions of reporters as compared to called reporters. The right columns contain bead density estimates based on fluorescent quantification of single-stranded DNA in the libraries. Following estimates were made: i) an average of 800,000 oligonucleotides clustered on each bead, and ii) oligonucleotides have an average length of 114.8-bp, resulting in a single strand DNA molecular weight of 34943.76 g/mole (number of nucleotides x 303.7 + 79.0). Calculation of weight/bead = (34943.76/Avogadro’s constant) x 800,000. The table is summarized with average (AVG) and standard deviation (STDEV) values for all components. Furthermore maximum (MAXDEV) and minimum (MINDEV) values refer to the upper and lower deviation from the average value.
(PDF)

Table S3 **Metadata for background noise negative (water) selections in six replicates (X1-X6).** The table contains Torrent Suite and Sphix-generated data from the sequencing runs performed on the interrogated libraries. Calls denote actual sequence read counts. Left columns contain sequence run information with reporter population and subcategories (defined in Table 1). Frequency denotes the fraction of the subcategories compared to the reporter population. Right columns contain sequence read counts for reporter distribution, and frequency denotes the fractions of reporters as compared to called reporters. The table is summarized with average (AVG) and standard deviation (STDEV) values for all components. Furthermore maximum (MAXDEV) and minimum (MINDEV) values refer to the upper and lower deviation from the average value. The last row of the table details the threshold line based on the statistical three-sigma rule (mean + 3x standard deviation) for the different reporters. The highest value (reporter-56) was chosen for a conservative threshold line, which equals ~1261.5 reads.
(PDF)

Table S4 **Metadata for quintuple Sequencing Bead Array (SBA) assay selections of HPV plasmids (HPV-16, HPV-18, HPV-33, and HPV-59) in six replicates (X1-X6).** The table contains Torrent Suite and Sphix-generated data from the sequencing runs performed on the interrogated libraries. Calls denote actual sequence read counts. Left columns contain sequence run information with reporter population and subcategories (defined in Table 1). Frequency denotes the fraction of the subcategories compared to the reporter population. Right columns contain sequence read counts for reporter distribution, and frequency denotes the fractions of reporters as compared to called reporters. The table is summarized with average (AVG) and standard deviation (STDEV) values for all components. Furthermore maximum (MAXDEV) and minimum (MINDEV) values refer to the upper and lower deviation from the average value.
(PDF)

Table S5 **Metadata for Sequencing Bead Array (SBA) assay screens of 20 cervical tumor samples.** Cervical tumor samples (extracted genomic DNA) derived from different females (OM-1078, OM-1272, OM-1299, OM-1301, OM-1452, OM-1464, OM-1530, OM-1569, OM-1668, OM-1741, OM-1751, OM-1848, OM-1854, OM-1967, OM-1980, OM-2006, OM-2059, OM-2215, OM-2257, and OM-2258). The table contains Torrent Suite and Sphix-generated data from the sequencing runs performed on the interrogated libraries. Calls denote actual sequence read counts. Left columns contain sequence run information with reporter population and subcategories (defined in Table 1). Frequency denotes the fraction of the subcategories compared to the reporter population. Right columns contain sequence read counts for reporter distribution, and frequency denotes the fractions of reporters as compared to called reporters.
(PDF)
