# Supplementary material for: The Sequencing Bead Array (SBA), a Next-Generation Digital Suspension Array
Source: PLoS One. 2013 Oct 7;8(10):e76696. doi: 10.1371/journal.pone.0076696 (PMC3792038; doi:10.1371/journal.pone.0076696)
Supplement: Data S1 — Raw sequencing data files for presented experiments. The compressed file contains a table of content for all files included (0. TOC Supporting Data Files.txt) and raw sequencing data (PGM Torrent Suite run reports, FASTQ files and Sphix generated CSV files) for experiments presented in Figure 2 and Figure 3 (same data set as Figure S1). (ZIP) [file pone.0076696.s001.zip › Supporting Data Files/1. Figure 2/1.1 Figure 2a files/1.1.1 TS Reports/1_Figure_2a_L1_TS-342.pdf]

Run Summary

**10.4M** Total Bases	**85** Key Signal

56%
Bead Loading

ISP Density

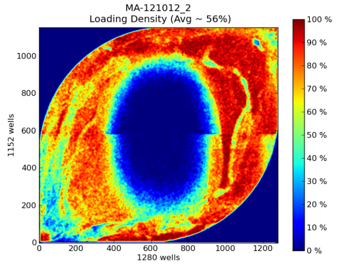

371,694
Total Reads

57%
Usable Sequence

ISP Summary

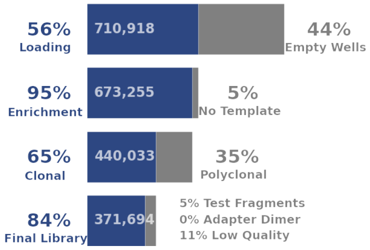

28 bp
Mean Read Length

Read Length

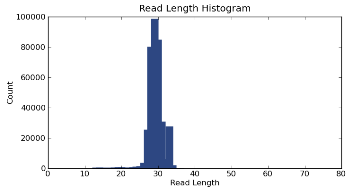

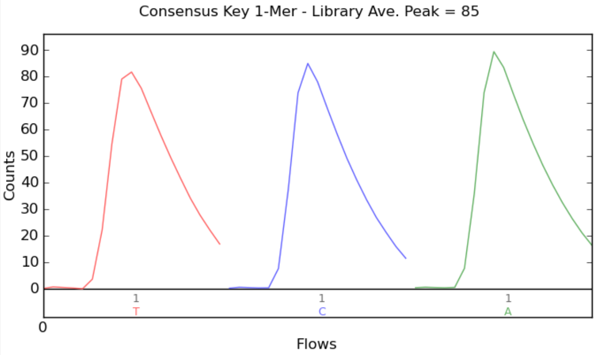

**Addressable Wells**	**1,262,519**	
With ISPs	710,918	56.3%
Live	673,255	94.7%
Test Fragment	22,956	03.4%
Library	650,299	96.6%

**Library ISPs**	**650,299**
Filtered: Polyclonal	233,222
Filtered: Low Quality	45,246
Filtered: Primer Dimer	137
**Final Library ISPs**	**371,694**

Test Fragment	Reads	Percent 50AQ17	Read Length Histogram
**TF_A**	**22,057**	**91%**	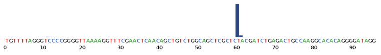
