# Supplementary material for: The Sequencing Bead Array (SBA), a Next-Generation Digital Suspension Array
Source: PLoS One. 2013 Oct 7;8(10):e76696. doi: 10.1371/journal.pone.0076696 (PMC3792038; doi:10.1371/journal.pone.0076696)
Supplement: Data S1 — Raw sequencing data files for presented experiments. The compressed file contains a table of content for all files included (0. TOC Supporting Data Files.txt) and raw sequencing data (PGM Torrent Suite run reports, FASTQ files and Sphix generated CSV files) for experiments presented in Figure 2 and Figure 3 (same data set as Figure S1). (ZIP) [file pone.0076696.s001.zip › Supporting Data Files/1. Figure 2/1.1 Figure 2a files/1.1.1 TS Reports/2_Figure_2a_L2_TS-342.pdf]

Run Summary

**10.9M**	**85**
Total Bases	Key Signal

65%
Bead Loading

ISP Density

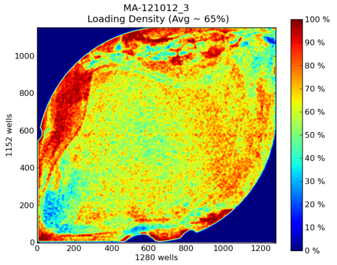

389,623
Total Reads

53%
Usable Sequence

ISP Summary

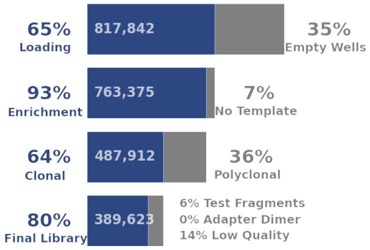

28 bp
Mean Read Length

Read Length

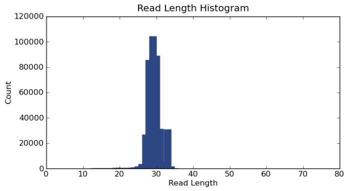

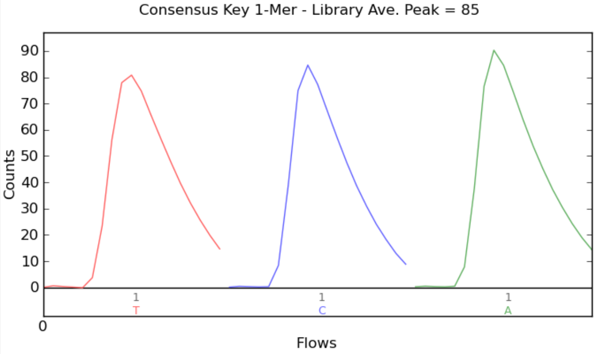

**Addressable Wells**	**1,262,519**	
With ISPs	817,842	64.8%
Live	763,375	93.3%
Test Fragment	29,518	03.9%
Library	733,857	96.1%

**Library ISPs**	**733,857**
Filtered: Polyclonal	275,463
Filtered: Low Quality	68,631
Filtered: Primer Dimer	140
**Final Library ISPs**	**389,623**

Test Fragment	Reads	Percent 50AQ17	Read Length Histogram
**TF_A**	**28,251**	**90%**	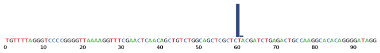

ion torrent
by life technologies
