# Supplementary material for: The Sequencing Bead Array (SBA), a Next-Generation Digital Suspension Array
Source: PLoS One. 2013 Oct 7;8(10):e76696. doi: 10.1371/journal.pone.0076696 (PMC3792038; doi:10.1371/journal.pone.0076696)
Supplement: Data S1 — Raw sequencing data files for presented experiments. The compressed file contains a table of content for all files included (0. TOC Supporting Data Files.txt) and raw sequencing data (PGM Torrent Suite run reports, FASTQ files and Sphix generated CSV files) for experiments presented in Figure 2 and Figure 3 (same data set as Figure S1). (ZIP) [file pone.0076696.s001.zip › Supporting Data Files/1. Figure 2/1.1 Figure 2a files/1.1.1 TS Reports/3_Figure_2a_L3_TS-342.pdf]

Run Summary

10.7M	82
Total Bases	Key Signal

62%
Bead Loading

ISP Density

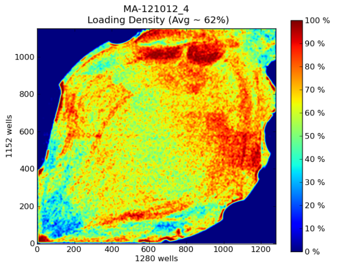

379,559
Total Reads

55%
Usable Sequence

ISP Summary

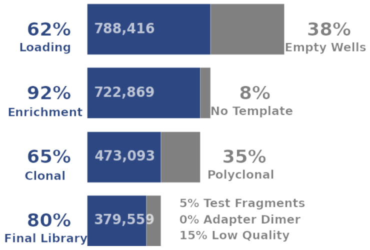

28 bp
Mean Read Length

Read Length

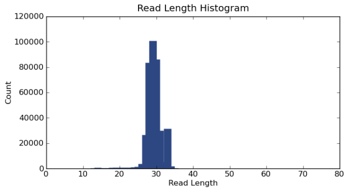

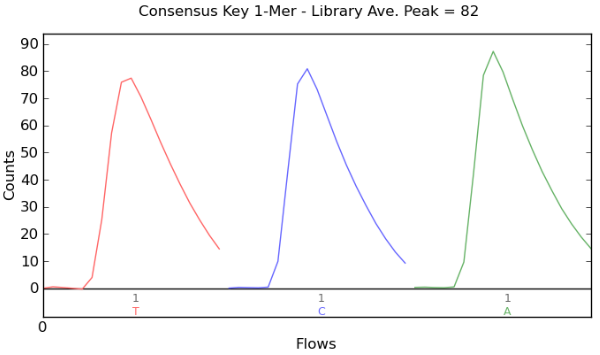

Addressable Wells	1,262,519	
With ISPs	788,416	62.4%
Live	722,869	91.7%
Test Fragment	26,435	03.7%
Library	696,434	96.3%

Library ISPs	696,434
Filtered: Polyclonal	249,776
Filtered: Low Quality	66,937
Filtered: Primer Dimer	162
**Final Library ISPs**	**379,559**

Test Fragment	Reads	Percent 50AQ17	Read Length Histogram
**TF_A**	**24,678**	**88%**	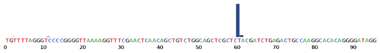
