# Supplementary material for: The Sequencing Bead Array (SBA), a Next-Generation Digital Suspension Array
Source: PLoS One. 2013 Oct 7;8(10):e76696. doi: 10.1371/journal.pone.0076696 (PMC3792038; doi:10.1371/journal.pone.0076696)
Supplement: Data S1 — Raw sequencing data files for presented experiments. The compressed file contains a table of content for all files included (0. TOC Supporting Data Files.txt) and raw sequencing data (PGM Torrent Suite run reports, FASTQ files and Sphix generated CSV files) for experiments presented in Figure 2 and Figure 3 (same data set as Figure S1). (ZIP) [file pone.0076696.s001.zip › Supporting Data Files/1. Figure 2/1.1 Figure 2a files/1.1.1 TS Reports/4_Figure_2a_L4_TS-342.pdf]

Run Summary

10.9M Total Bases | **82** Key Signal

65% Bead Loading

ISP Density

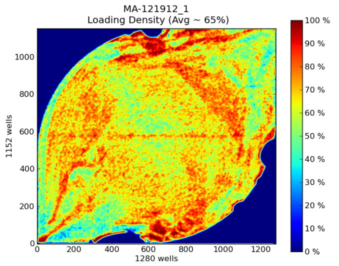

388,130 Total Reads

51% Usable Sequence

ISP Summary

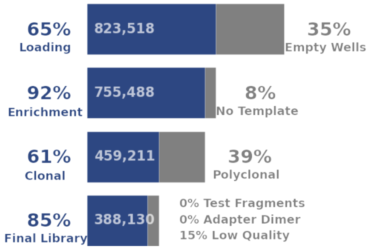

28 bp Mean Read Length

Read Length

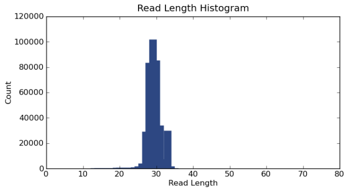

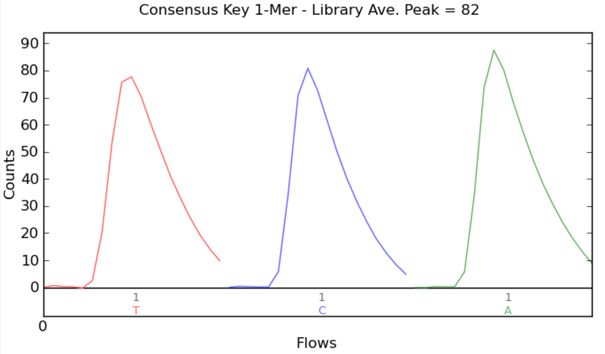

Addressable Wells	1,262,519	
With ISPs	823,518	65.2%
Live	755,488	91.7%
Test Fragment	155	00.0%
Library	755,333	100.0%

Library ISPs	755,333
Filtered: Polyclonal	296,277
Filtered: Low Quality	70,741
Filtered: Primer Dimer	185
**Final Library ISPs**	**388,130**

ion torrent by life technologies
