# Supplementary material for: The Sequencing Bead Array (SBA), a Next-Generation Digital Suspension Array
Source: PLoS One. 2013 Oct 7;8(10):e76696. doi: 10.1371/journal.pone.0076696 (PMC3792038; doi:10.1371/journal.pone.0076696)
Supplement: Data S1 — Raw sequencing data files for presented experiments. The compressed file contains a table of content for all files included (0. TOC Supporting Data Files.txt) and raw sequencing data (PGM Torrent Suite run reports, FASTQ files and Sphix generated CSV files) for experiments presented in Figure 2 and Figure 3 (same data set as Figure S1). (ZIP) [file pone.0076696.s001.zip › Supporting Data Files/1. Figure 2/1.1 Figure 2a files/1.1.1 TS Reports/6_Figure_2a_L6_TS-342.pdf]

# Run Summary

**10.6M** Total Bases

**78** Key Signal

**57%** Bead Loading

## ISP Density

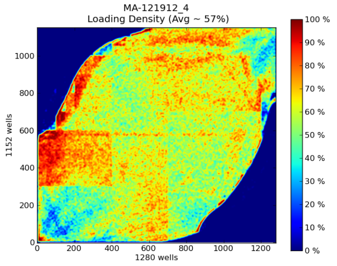


**377,059** Total Reads

**59%** Usable Sequence

## ISP Summary

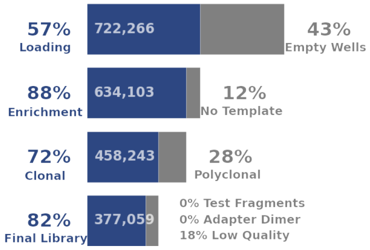


**28 bp** Mean Read Length

## Read Length

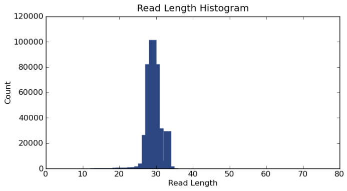


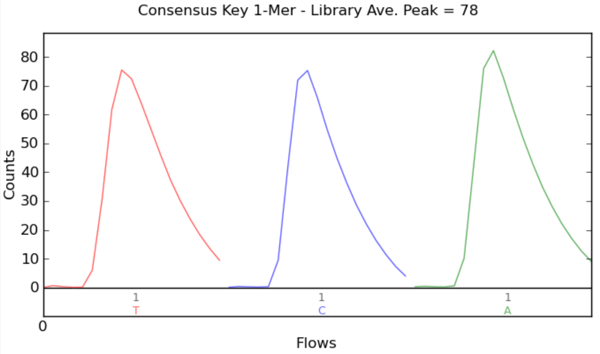


| Addressable Wells | 1,262,519 | |
|---|---|---|
| With ISPs | 722,266 | 57.2% |
| Live | 634,103 | 87.8% |
| Test Fragment | 257 | 00.0% |
| Library | 633,846 | 100.0% |

| **Library ISPs** | **633,846** |
|---|---|
| Filtered: Polyclonal | 175,860 |
| Filtered: Low Quality | 80,764 |
| Filtered: Primer Dimer | 163 |
| **Final Library ISPs** | **377,059** |
