# Supplementary material for: The Sequencing Bead Array (SBA), a Next-Generation Digital Suspension Array
Source: PLoS One. 2013 Oct 7;8(10):e76696. doi: 10.1371/journal.pone.0076696 (PMC3792038; doi:10.1371/journal.pone.0076696)
Supplement: Data S1 — Raw sequencing data files for presented experiments. The compressed file contains a table of content for all files included (0. TOC Supporting Data Files.txt) and raw sequencing data (PGM Torrent Suite run reports, FASTQ files and Sphix generated CSV files) for experiments presented in Figure 2 and Figure 3 (same data set as Figure S1). (ZIP) [file pone.0076696.s001.zip › Supporting Data Files/1. Figure 2/1.2 Figure 2b files/1.2.1 TS Reports/1_Figure_2b_NEGATIVE_X1_TS-342.pdf]

Run Summary

98K
Total Bases

15
Key Signal

20%
Bead Loading

ISP Density

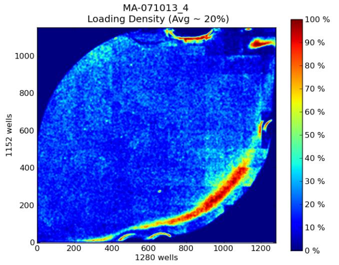

3,482
Total Reads

23%
Usable Sequence

ISP Summary

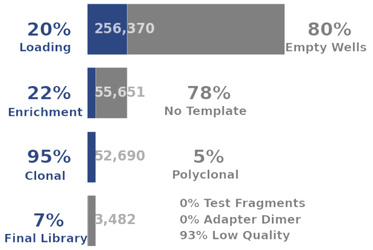

28 bp
Mean Read Length

Read Length

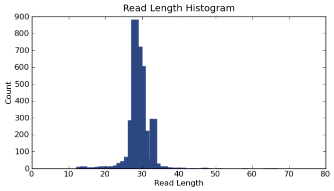

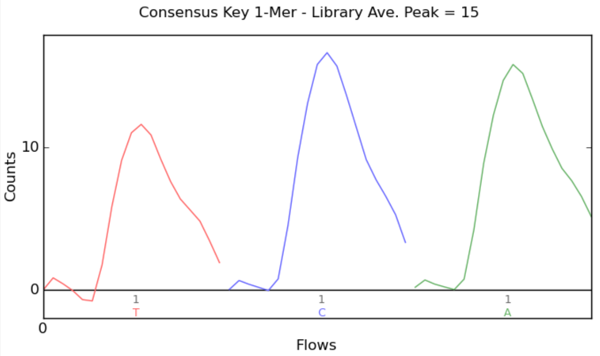

**Addressable Wells**	**1,262,519**	
With ISPs	256,370	20.3%
Live	55,651	21.7%
Test Fragment	40,420	72.6%
Library	15,231	27.4%

**Library ISPs**	**15,231**
Filtered: Polyclonal	2,961
Filtered: Low Quality	8,787
Filtered: Primer Dimer	1
**Final Library ISPs**	**3,482**

ion torrent
by life technologies™
