# Supplementary material for: The Sequencing Bead Array (SBA), a Next-Generation Digital Suspension Array
Source: PLoS One. 2013 Oct 7;8(10):e76696. doi: 10.1371/journal.pone.0076696 (PMC3792038; doi:10.1371/journal.pone.0076696)
Supplement: Data S1 — Raw sequencing data files for presented experiments. The compressed file contains a table of content for all files included (0. TOC Supporting Data Files.txt) and raw sequencing data (PGM Torrent Suite run reports, FASTQ files and Sphix generated CSV files) for experiments presented in Figure 2 and Figure 3 (same data set as Figure S1). (ZIP) [file pone.0076696.s001.zip › Supporting Data Files/1. Figure 2/1.2 Figure 2b files/1.2.1 TS Reports/2_Figure_2b_NEGATIVE_X2_TS-342.pdf]

Run Summary

165K Total Bases

57 Key Signal

17% Bead Loading

ISP Density

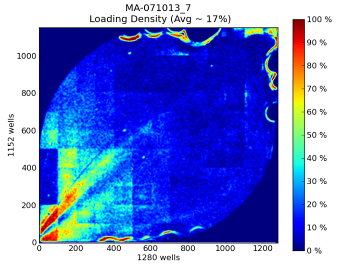

5,955 Total Reads

28% Usable Sequence

ISP Summary

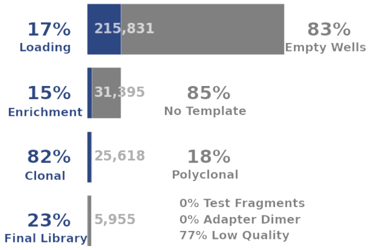

27 bp Mean Read Length

Read Length

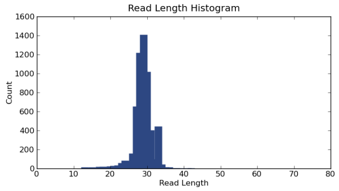

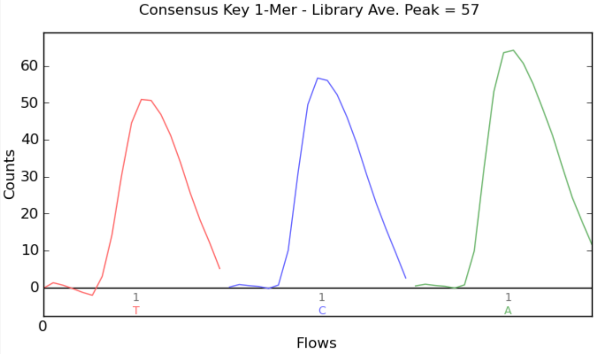

**Addressable Wells**	**1,262,519**	
With ISPs	215,831	17.1%
Live	31,395	14.5%
Test Fragment	9,827	31.3%
Library	21,568	68.7%

**Library ISPs**	**21,568**
Filtered: Polyclonal	5,777
Filtered: Low Quality	9,833
Filtered: Primer Dimer	3
**Final Library ISPs**	**5,955**
