# Supplementary material for: The Sequencing Bead Array (SBA), a Next-Generation Digital Suspension Array
Source: PLoS One. 2013 Oct 7;8(10):e76696. doi: 10.1371/journal.pone.0076696 (PMC3792038; doi:10.1371/journal.pone.0076696)
Supplement: Data S1 — Raw sequencing data files for presented experiments. The compressed file contains a table of content for all files included (0. TOC Supporting Data Files.txt) and raw sequencing data (PGM Torrent Suite run reports, FASTQ files and Sphix generated CSV files) for experiments presented in Figure 2 and Figure 3 (same data set as Figure S1). (ZIP) [file pone.0076696.s001.zip › Supporting Data Files/1. Figure 2/1.2 Figure 2b files/1.2.1 TS Reports/3_Figure_2b_NEGATIVE_X3_TS-342.pdf]

Run Summary

36.7K
Total Bases

11
Key Signal

53%
Bead Loading

ISP Density

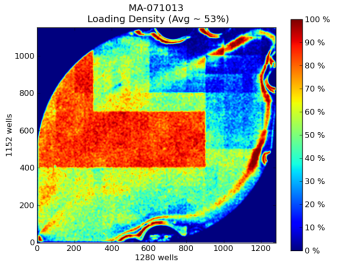

1,294
Total Reads

12%
Usable Sequence

ISP Summary

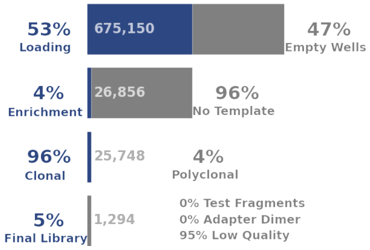

28 bp
Mean Read Length

Read Length

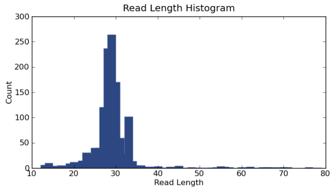

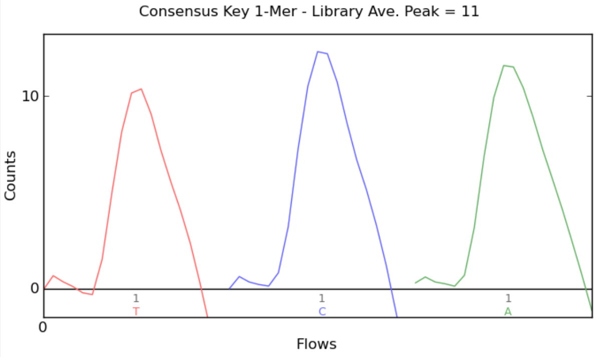

Addressable Wells	1,262,519	
With ISPs	675,150	53.5%
Live	26,856	04.0%
Test Fragment	16,331	60.8%
Library	10,525	39.2%

Library ISPs	10,525
Filtered: Polyclonal	1,108
Filtered: Low Quality	8,122
Filtered: Primer Dimer	1
**Final Library ISPs**	**1,294**

ion torrent
by life technologies™
