# Supplementary material for: The Sequencing Bead Array (SBA), a Next-Generation Digital Suspension Array
Source: PLoS One. 2013 Oct 7;8(10):e76696. doi: 10.1371/journal.pone.0076696 (PMC3792038; doi:10.1371/journal.pone.0076696)
Supplement: Data S1 — Raw sequencing data files for presented experiments. The compressed file contains a table of content for all files included (0. TOC Supporting Data Files.txt) and raw sequencing data (PGM Torrent Suite run reports, FASTQ files and Sphix generated CSV files) for experiments presented in Figure 2 and Figure 3 (same data set as Figure S1). (ZIP) [file pone.0076696.s001.zip › Supporting Data Files/1. Figure 2/1.2 Figure 2b files/1.2.1 TS Reports/5_Figure_2b_NEGATIVE_X5_TS-342.pdf]

Run Summary

89K Total Bases

16 Key Signal

38% Bead Loading

ISP Density

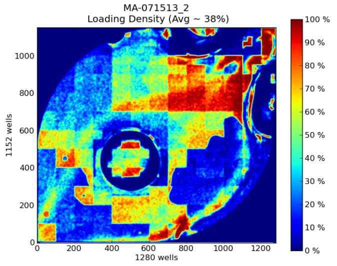

3,178 Total Reads

25% Usable Sequence

ISP Summary

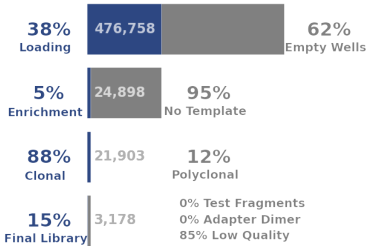

28 bp Mean Read Length

Read Length

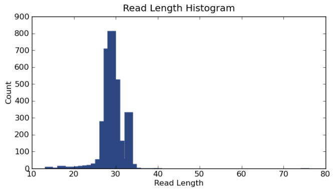

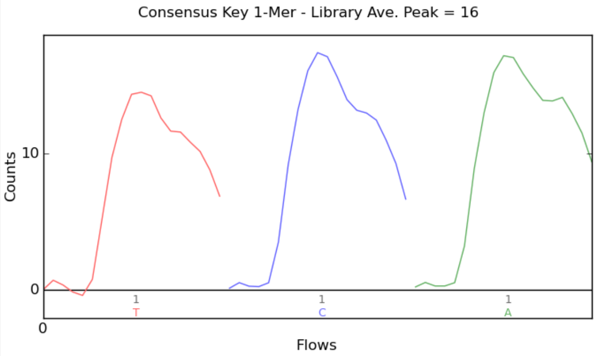

Addressable Wells	1,262,519	
With ISPs	476,758	37.8%
Live	24,898	05.2%
Test Fragment	12,072	48.5%
Library	12,826	51.5%

Library ISPs	12,826
Filtered: Polyclonal	2,995
Filtered: Low Quality	6,652
Filtered: Primer Dimer	1
**Final Library ISPs**	**3,178**

ion torrent by life technologies
