# Supplementary material for: The Sequencing Bead Array (SBA), a Next-Generation Digital Suspension Array
Source: PLoS One. 2013 Oct 7;8(10):e76696. doi: 10.1371/journal.pone.0076696 (PMC3792038; doi:10.1371/journal.pone.0076696)
Supplement: Data S1 — Raw sequencing data files for presented experiments. The compressed file contains a table of content for all files included (0. TOC Supporting Data Files.txt) and raw sequencing data (PGM Torrent Suite run reports, FASTQ files and Sphix generated CSV files) for experiments presented in Figure 2 and Figure 3 (same data set as Figure S1). (ZIP) [file pone.0076696.s001.zip › Supporting Data Files/1. Figure 2/1.2 Figure 2b files/1.2.1 TS Reports/6_Figure_2b_NEGATIVE_X6_TS-342.pdf]

Run Summary

39.5K Total Bases

9 Key Signal

27% Bead Loading

ISP Density

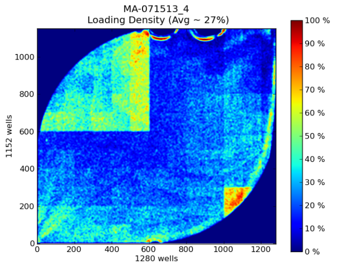

1,373 Total Reads

13% Usable Sequence

ISP Summary

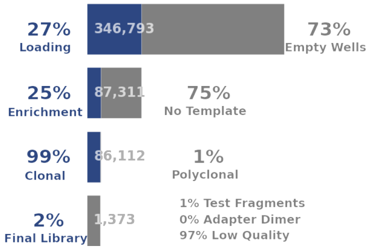

28 bp Mean Read Length

Read Length

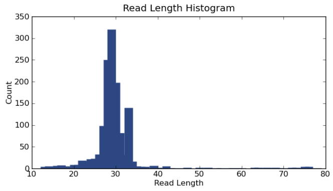

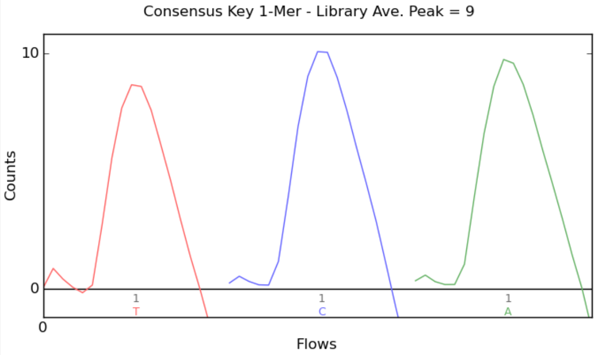

**Addressable Wells**	**1,262,519**	
With ISPs	346,793	27.5%
Live	87,311	25.2%
Test Fragment	76,955	88.1%
Library	10,356	11.9%

**Library ISPs**	**10,356**
Filtered: Polyclonal	1,199
Filtered: Low Quality	7,783
Filtered: Primer Dimer	1
**Final Library ISPs**	**1,373**
