# Supplementary material for: The Sequencing Bead Array (SBA), a Next-Generation Digital Suspension Array
Source: PLoS One. 2013 Oct 7;8(10):e76696. doi: 10.1371/journal.pone.0076696 (PMC3792038; doi:10.1371/journal.pone.0076696)
Supplement: Data S1 — Raw sequencing data files for presented experiments. The compressed file contains a table of content for all files included (0. TOC Supporting Data Files.txt) and raw sequencing data (PGM Torrent Suite run reports, FASTQ files and Sphix generated CSV files) for experiments presented in Figure 2 and Figure 3 (same data set as Figure S1). (ZIP) [file pone.0076696.s001.zip › Supporting Data Files/1. Figure 2/1.3 Figure 2c files/1.3.1 TS Reports/3_Figure_2c_QUINTUPLE_X3_TS-342.pdf]

Run Summary

**1.8M**	**88**
Total Bases	Key Signal

28%
Bead Loading

ISP Density

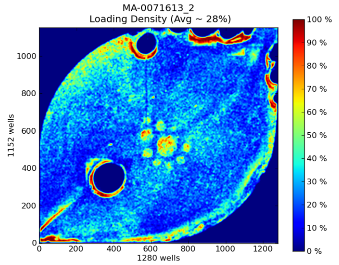

67,273
Total Reads

30%
Usable Sequence

ISP Summary

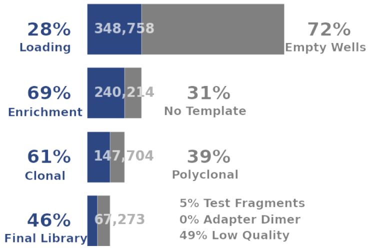

27 bp
Mean Read Length

Read Length

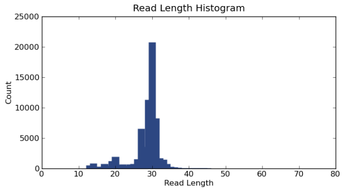

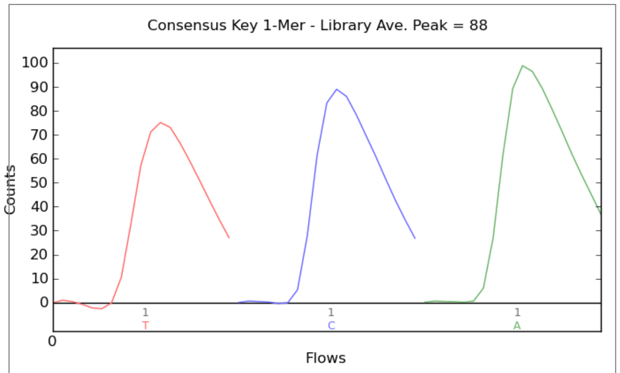

**Addressable Wells**	**1,262,530**	
With ISPs	348,758	27.6%
Live	240,214	68.9%
Test Fragment	13,439	05.6%
Library	226,775	94.4%

**Library ISPs**	**226,775**
Filtered: Polyclonal	92,510
Filtered: Low Quality	66,990
Filtered: Primer Dimer	2
**Final Library ISPs**	**67,273**

Test Fragment	Reads	Percent 50AQ17	Read Length Histogram
**TF_A**	**4,819**	**14%**	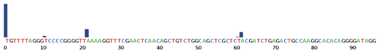

ion torrent by life technologies
