# Supplementary material for: The Sequencing Bead Array (SBA), a Next-Generation Digital Suspension Array
Source: PLoS One. 2013 Oct 7;8(10):e76696. doi: 10.1371/journal.pone.0076696 (PMC3792038; doi:10.1371/journal.pone.0076696)
Supplement: Data S1 — Raw sequencing data files for presented experiments. The compressed file contains a table of content for all files included (0. TOC Supporting Data Files.txt) and raw sequencing data (PGM Torrent Suite run reports, FASTQ files and Sphix generated CSV files) for experiments presented in Figure 2 and Figure 3 (same data set as Figure S1). (ZIP) [file pone.0076696.s001.zip › Supporting Data Files/2. Figure 3 and S1/2.1 Figure 3 and S1 files/2.1 TS Reports/11_OM-1751_TS-342.pdf]

Run Summary

142K Total Bases | **66** Key Signal

27% Bead Loading

ISP Density

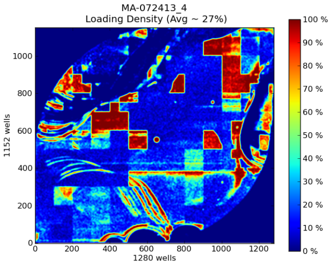

5,053 Total Reads

25% Usable Sequence

ISP Summary

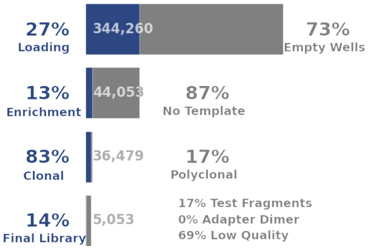

28 bp Mean Read Length

Read Length

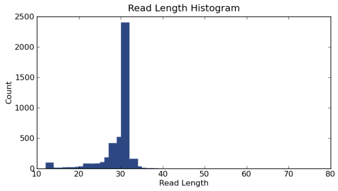

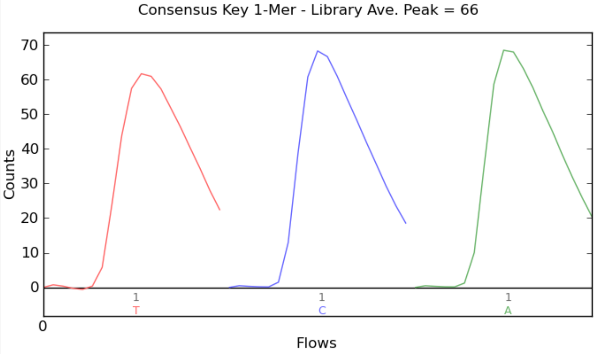

**Addressable Wells**	**1,262,528**	
With ISPs	344,260	27.3%
Live	44,053	12.8%
Test Fragment	23,888	54.2%
Library	20,165	45.8%

**Library ISPs**	**20,165**
Filtered: Polyclonal	7,574
Filtered: Low Quality	7,538
Filtered: Primer Dimer	0
**Final Library ISPs**	**5,053**

Test Fragment	Reads	Percent 50AQ17	Read Length Histogram
**TF_A**	**5,596**	**84%**	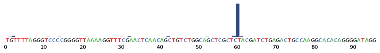

ion torrent by life technologies
