# Supplementary material for: The Sequencing Bead Array (SBA), a Next-Generation Digital Suspension Array
Source: PLoS One. 2013 Oct 7;8(10):e76696. doi: 10.1371/journal.pone.0076696 (PMC3792038; doi:10.1371/journal.pone.0076696)
Supplement: Data S1 — Raw sequencing data files for presented experiments. The compressed file contains a table of content for all files included (0. TOC Supporting Data Files.txt) and raw sequencing data (PGM Torrent Suite run reports, FASTQ files and Sphix generated CSV files) for experiments presented in Figure 2 and Figure 3 (same data set as Figure S1). (ZIP) [file pone.0076696.s001.zip › Supporting Data Files/2. Figure 3 and S1/2.1 Figure 3 and S1 files/2.1 TS Reports/16_OM-2006_TS-342.pdf]

Run Summary

167K Total Bases | **10** Key Signal

11%
Bead Loading

ISP Density

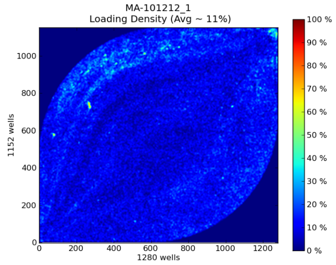

6,158
Total Reads

9%
Usable Sequence

ISP Summary

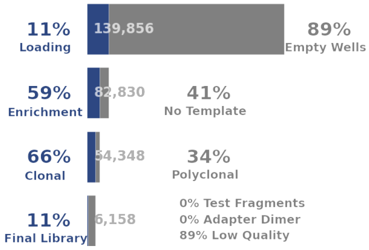

27 bp
Mean Read Length

Read Length

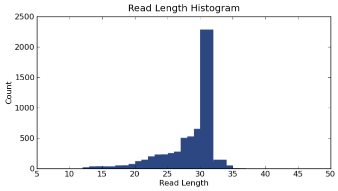

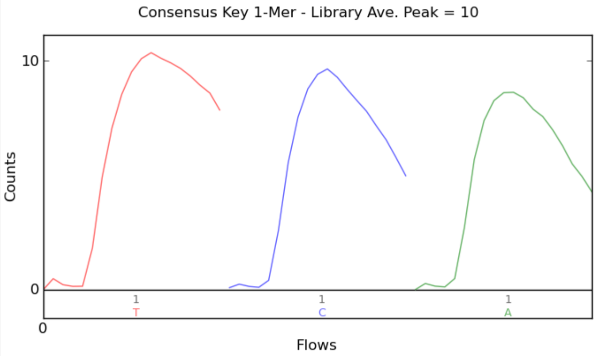

**Addressable Wells**	**1,262,519**	
With ISPs	139,856	11.1%
Live	82,830	59.2%
Test Fragment	12,541	15.1%
Library	70,289	84.9%

**Library ISPs**	**70,289**
Filtered: Polyclonal	28,482
Filtered: Low Quality	35,648
Filtered: Primer Dimer	1
**Final Library ISPs**	**6,158**
