# Supplementary material for: The Sequencing Bead Array (SBA), a Next-Generation Digital Suspension Array
Source: PLoS One. 2013 Oct 7;8(10):e76696. doi: 10.1371/journal.pone.0076696 (PMC3792038; doi:10.1371/journal.pone.0076696)
Supplement: Data S1 — Raw sequencing data files for presented experiments. The compressed file contains a table of content for all files included (0. TOC Supporting Data Files.txt) and raw sequencing data (PGM Torrent Suite run reports, FASTQ files and Sphix generated CSV files) for experiments presented in Figure 2 and Figure 3 (same data set as Figure S1). (ZIP) [file pone.0076696.s001.zip › Supporting Data Files/2. Figure 3 and S1/2.1 Figure 3 and S1 files/2.1 TS Reports/18_OM-2215_TS-342.pdf]

Run Summary

554K	36
Total Bases	Key Signal

18%
Bead Loading

ISP Density

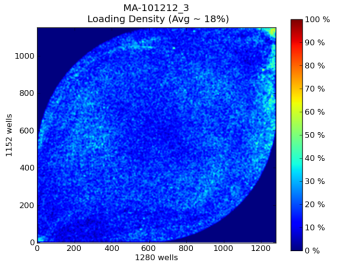

21,531
Total Reads

18%
Usable Sequence

ISP Summary

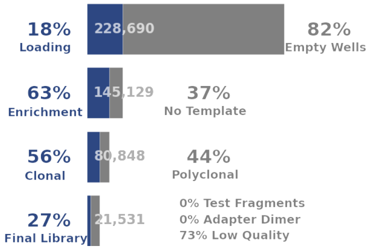

25 bp
Mean Read Length

Read Length

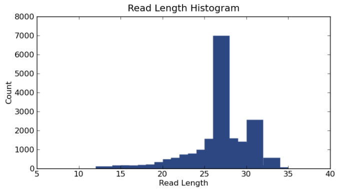

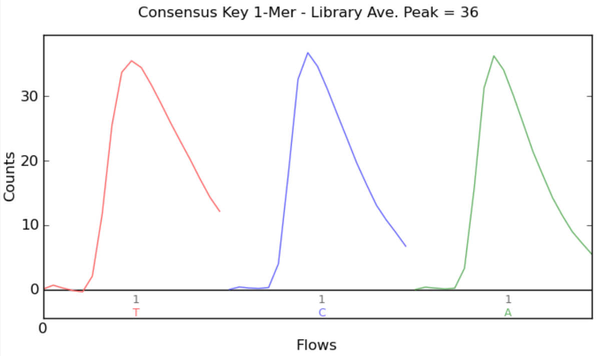

**Addressable Wells**	**1,262,522**	
With ISPs	228,690	18.1%
Live	145,129	63.5%
Test Fragment	24,361	16.8%
Library	120,768	83.2%

**Library ISPs**	**120,768**
Filtered: Polyclonal	64,281
Filtered: Low Quality	34,938
Filtered: Primer Dimer	18
**Final Library ISPs**	**21,531**
