# Supplementary material for: The Sequencing Bead Array (SBA), a Next-Generation Digital Suspension Array
Source: PLoS One. 2013 Oct 7;8(10):e76696. doi: 10.1371/journal.pone.0076696 (PMC3792038; doi:10.1371/journal.pone.0076696)
Supplement: Data S1 — Raw sequencing data files for presented experiments. The compressed file contains a table of content for all files included (0. TOC Supporting Data Files.txt) and raw sequencing data (PGM Torrent Suite run reports, FASTQ files and Sphix generated CSV files) for experiments presented in Figure 2 and Figure 3 (same data set as Figure S1). (ZIP) [file pone.0076696.s001.zip › Supporting Data Files/2. Figure 3 and S1/2.1 Figure 3 and S1 files/2.1 TS Reports/2_OM-1272_TS-342.pdf]

Run Summary

420K
Total Bases

38
Key Signal

17%
Bead Loading

ISP Density

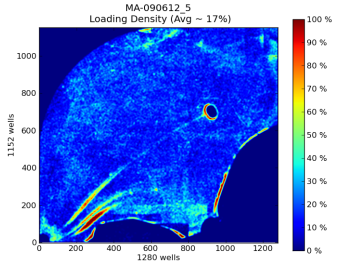

14,643
Total Reads

13%
Usable Sequence

ISP Summary

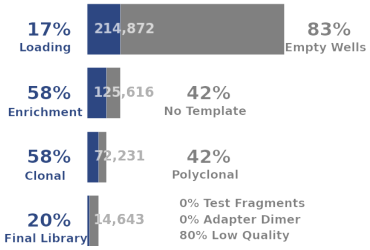

28 bp
Mean Read Length

Read Length

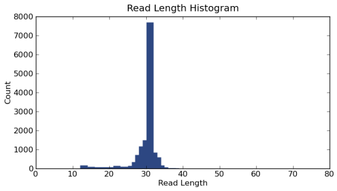

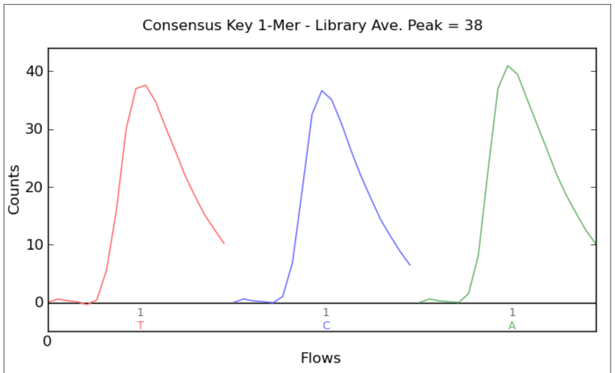

**Addressable Wells**	**1,263,924**	
With ISPs	214,872	17.0%
Live	125,616	58.5%
Test Fragment	9,646	07.7%
Library	115,970	92.3%

**Library ISPs**	**115,970**
Filtered: Polyclonal	53,385
Filtered: Low Quality	47,938
Filtered: Primer Dimer	4
**Final Library ISPs**	**14,643**
