# Supplementary figures and images for: The Sequencing Bead Array (SBA), a Next-Generation Digital Suspension Array
Source: PLoS One. 2013 Oct 7;8(10):e76696. doi: 10.1371/journal.pone.0076696 (PMC3792038; doi:10.1371/journal.pone.0076696)

## Run Summary

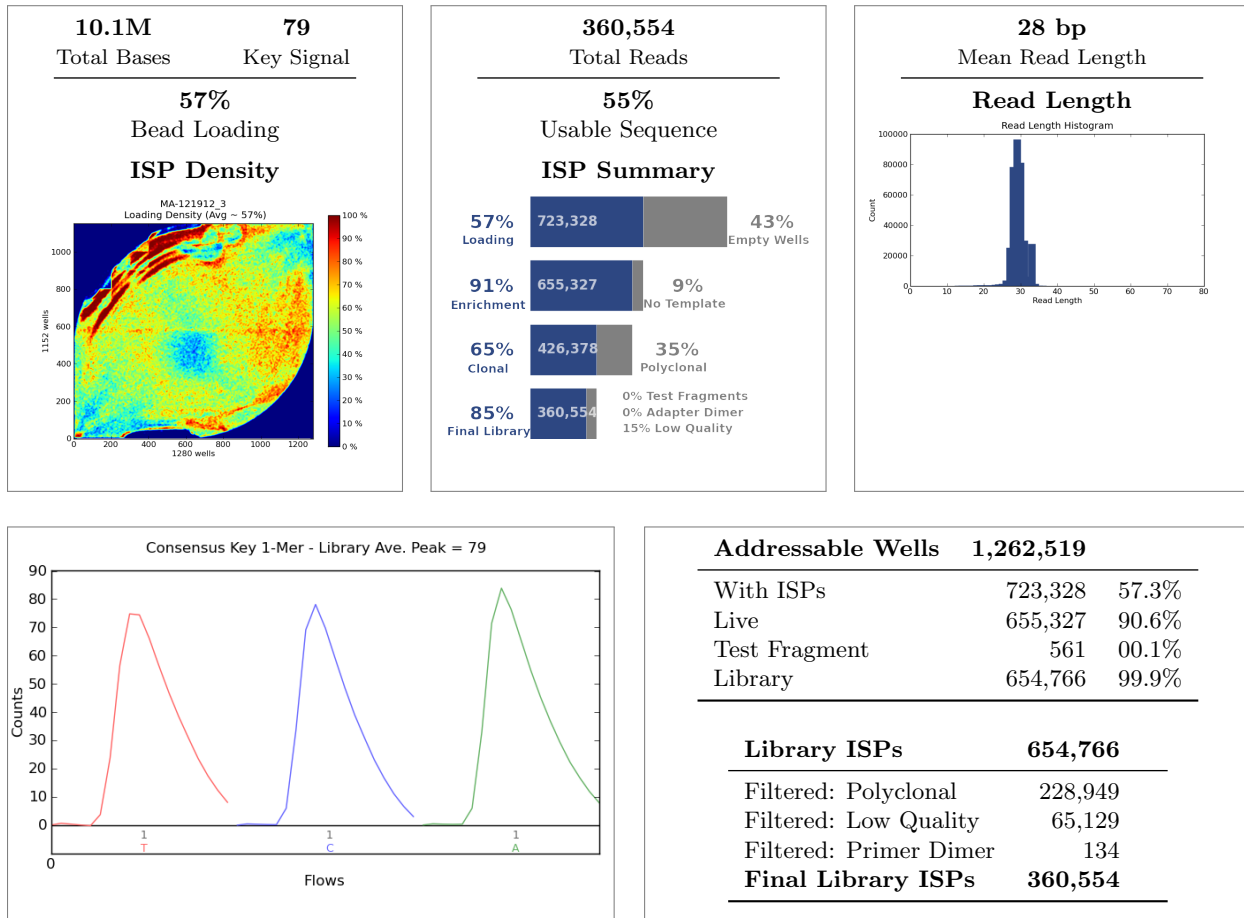

Supplement: Data S1 — Raw sequencing data files for presented experiments. The compressed file contains a table of content for all files included (0. TOC Supporting Data Files.txt) and raw sequencing data (PGM Torrent Suite run reports, FASTQ files and Sphix generated CSV files) for experiments presented in Figure 2 and Figure 3 (same data set as Figure S1). (ZIP) [file pone.0076696.s001.zip › Supporting Data Files/1. Figure 2/1.1 Figure 2a files/1.1.1 TS Reports/5_Figure_2a_L5_TS-342.pdf]

## Run Summary

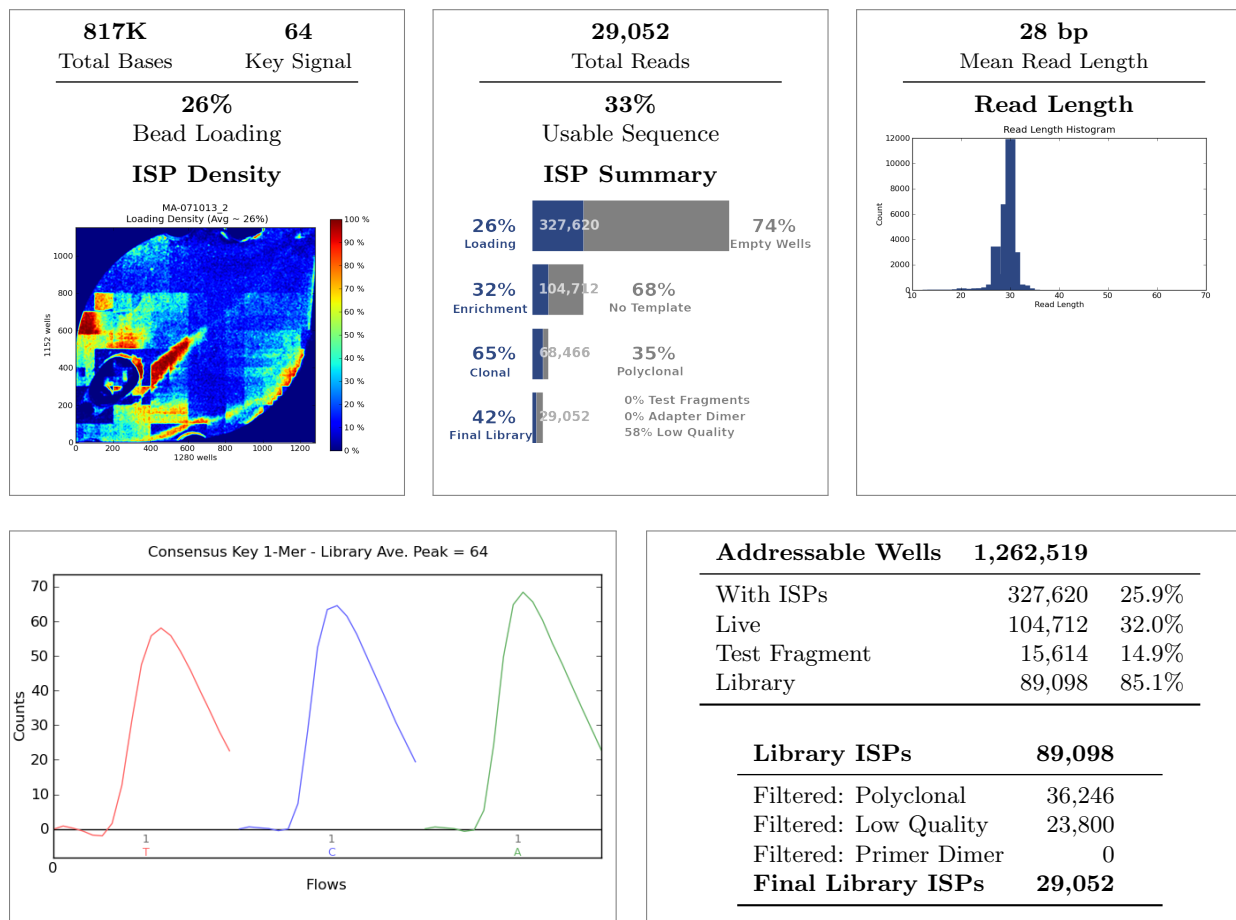

Supplement: Data S1 — Raw sequencing data files for presented experiments. The compressed file contains a table of content for all files included (0. TOC Supporting Data Files.txt) and raw sequencing data (PGM Torrent Suite run reports, FASTQ files and Sphix generated CSV files) for experiments presented in Figure 2 and Figure 3 (same data set as Figure S1). (ZIP) [file pone.0076696.s001.zip › Supporting Data Files/1. Figure 2/1.3 Figure 2c files/1.3.1 TS Reports/1_Figure_2c_QUINTUPLE_X1_TS-342.pdf]

## Run Summary

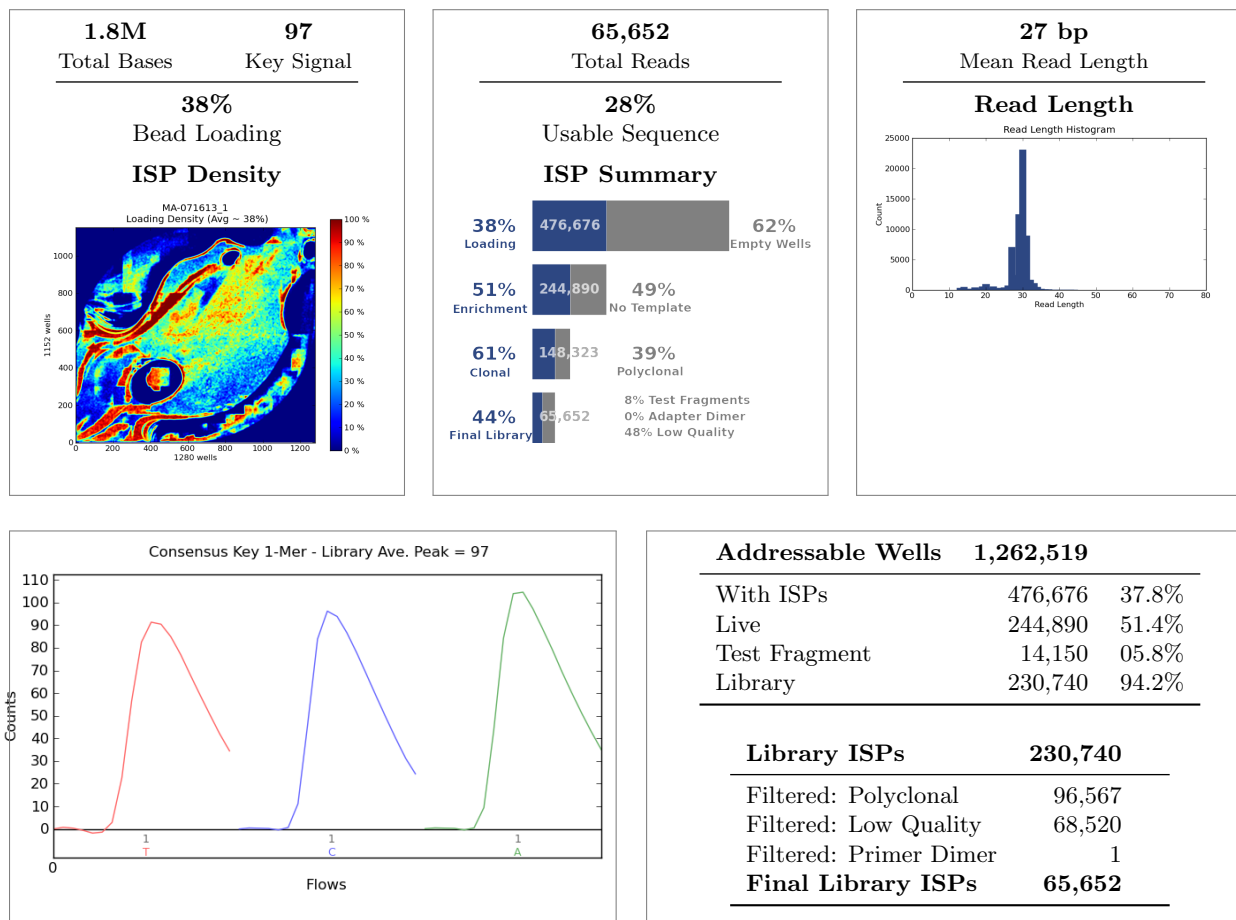

Test Fragment

Reads

Percent 50AQ17

Read Length Histogram

TF\_A

10,722

75%

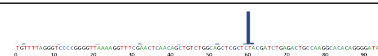

Supplement: Data S1 — Raw sequencing data files for presented experiments. The compressed file contains a table of content for all files included (0. TOC Supporting Data Files.txt) and raw sequencing data (PGM Torrent Suite run reports, FASTQ files and Sphix generated CSV files) for experiments presented in Figure 2 and Figure 3 (same data set as Figure S1). (ZIP) [file pone.0076696.s001.zip › Supporting Data Files/1. Figure 2/1.3 Figure 2c files/1.3.1 TS Reports/2_Figure_2c_QUINTUPLE_X2_TS-342.pdf]

## Run Summary

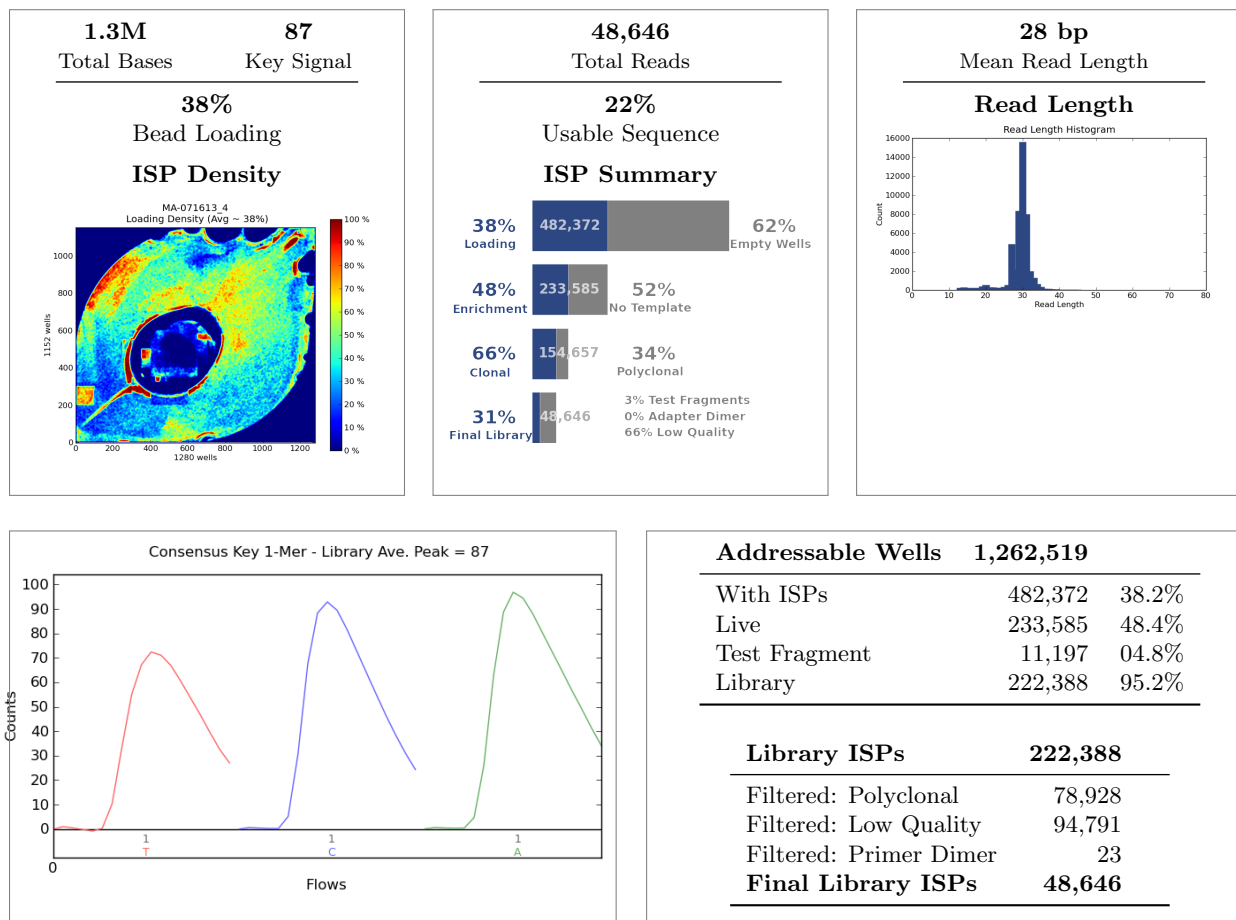

Test Fragment

Reads

Percent 50AQ17

Read Length Histogram

TF\_A

3,650

56%

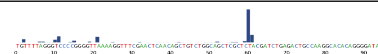

Supplement: Data S1 — Raw sequencing data files for presented experiments. The compressed file contains a table of content for all files included (0. TOC Supporting Data Files.txt) and raw sequencing data (PGM Torrent Suite run reports, FASTQ files and Sphix generated CSV files) for experiments presented in Figure 2 and Figure 3 (same data set as Figure S1). (ZIP) [file pone.0076696.s001.zip › Supporting Data Files/1. Figure 2/1.3 Figure 2c files/1.3.1 TS Reports/4_Figure_2c_QUINTUPLE_X4_TS-342.pdf]

## Run Summary

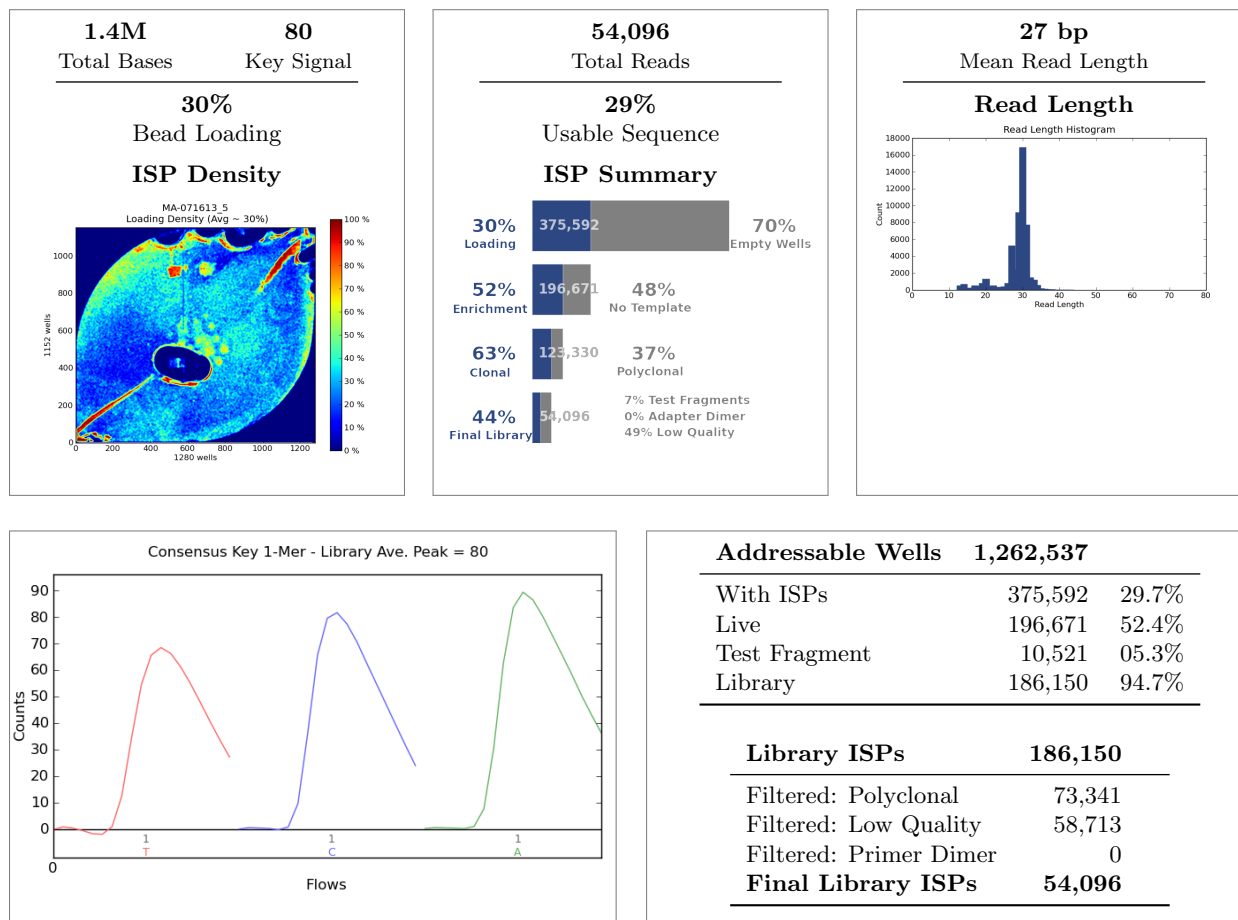

Test Fragment

Reads

Percent 50AQ17

Read Length Histogram

TF\_A

7,304

66%

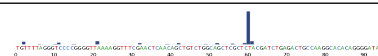

Supplement: Data S1 — Raw sequencing data files for presented experiments. The compressed file contains a table of content for all files included (0. TOC Supporting Data Files.txt) and raw sequencing data (PGM Torrent Suite run reports, FASTQ files and Sphix generated CSV files) for experiments presented in Figure 2 and Figure 3 (same data set as Figure S1). (ZIP) [file pone.0076696.s001.zip › Supporting Data Files/1. Figure 2/1.3 Figure 2c files/1.3.1 TS Reports/5_Figure_2c_QUINTUPLE_X5_TS-342.pdf]

Run Summary

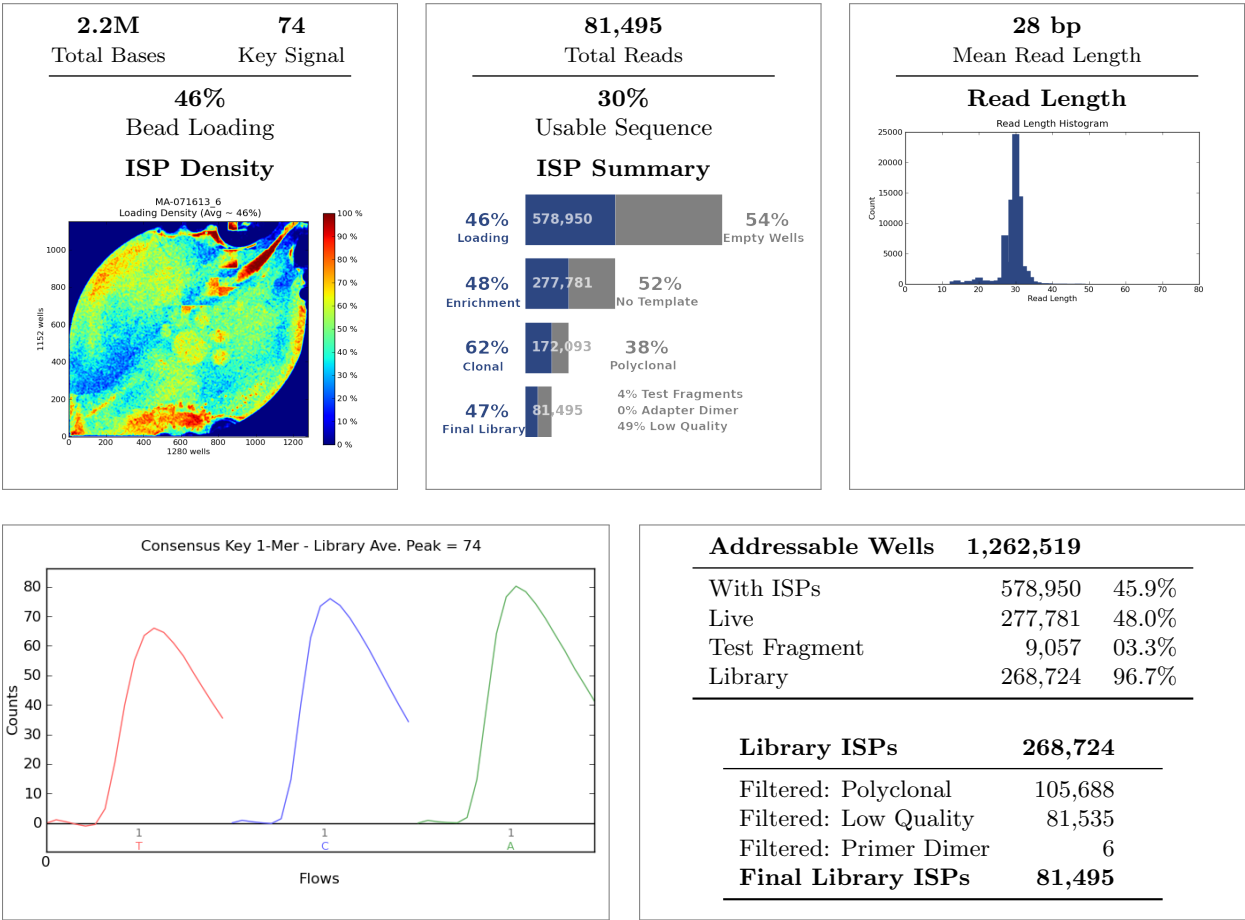

| Test Fragment | Reads | Percent 50AQ17 | Read Length Histogram                                                                |
|---------------|-------|----------------|--------------------------------------------------------------------------------------|
| TF_A          | 6,239 | 65%            | 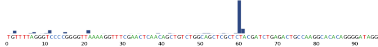 |

Supplement: Data S1 — Raw sequencing data files for presented experiments. The compressed file contains a table of content for all files included (0. TOC Supporting Data Files.txt) and raw sequencing data (PGM Torrent Suite run reports, FASTQ files and Sphix generated CSV files) for experiments presented in Figure 2 and Figure 3 (same data set as Figure S1). (ZIP) [file pone.0076696.s001.zip › Supporting Data Files/1. Figure 2/1.3 Figure 2c files/1.3.1 TS Reports/6_Figure_2c_QUINTUPLE_X6_TS-342.pdf]

## Run Summary

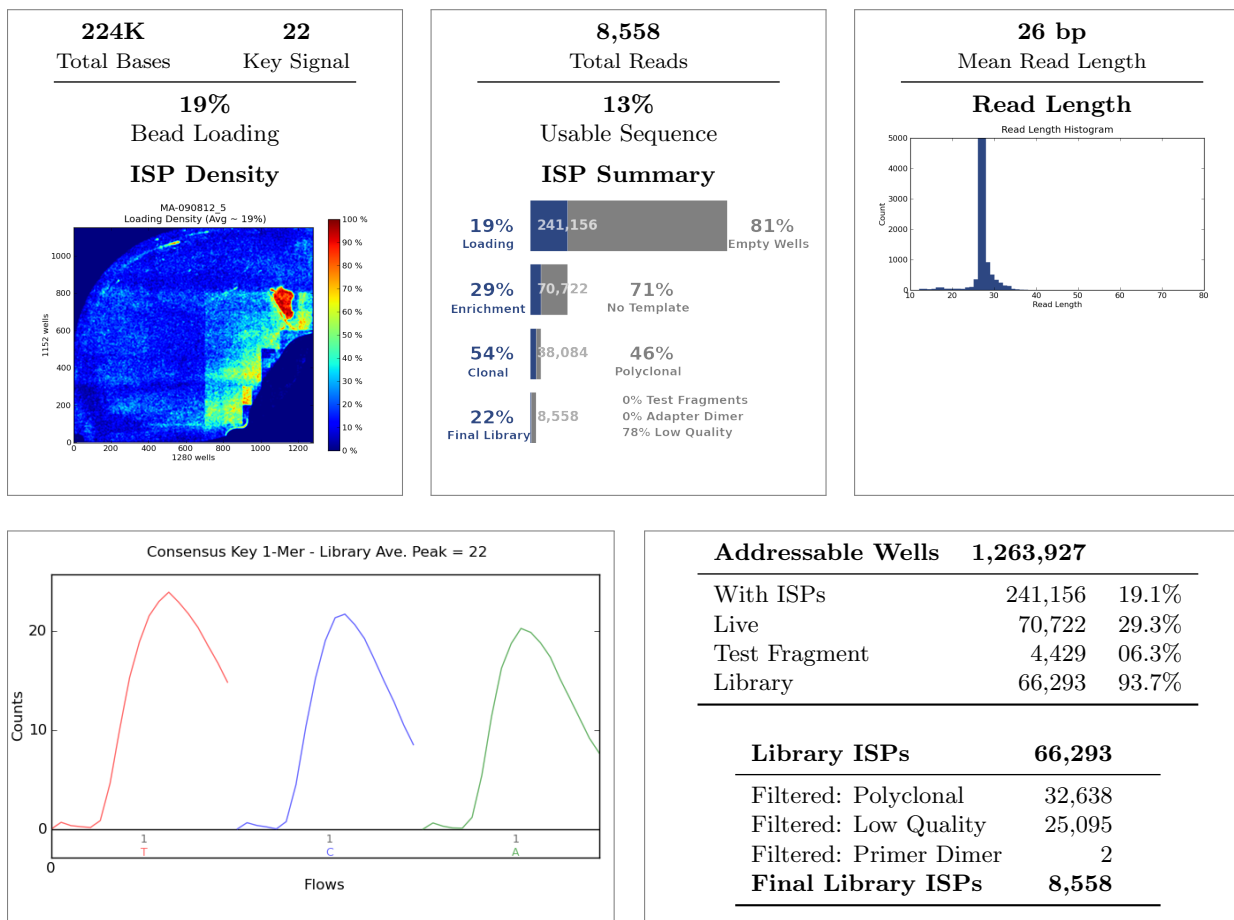

Supplement: Data S1 — Raw sequencing data files for presented experiments. The compressed file contains a table of content for all files included (0. TOC Supporting Data Files.txt) and raw sequencing data (PGM Torrent Suite run reports, FASTQ files and Sphix generated CSV files) for experiments presented in Figure 2 and Figure 3 (same data set as Figure S1). (ZIP) [file pone.0076696.s001.zip › Supporting Data Files/2. Figure 3 and S1/2.1 Figure 3 and S1 files/2.1 TS Reports/10_OM-1741_TS-342.pdf]

## Run Summary

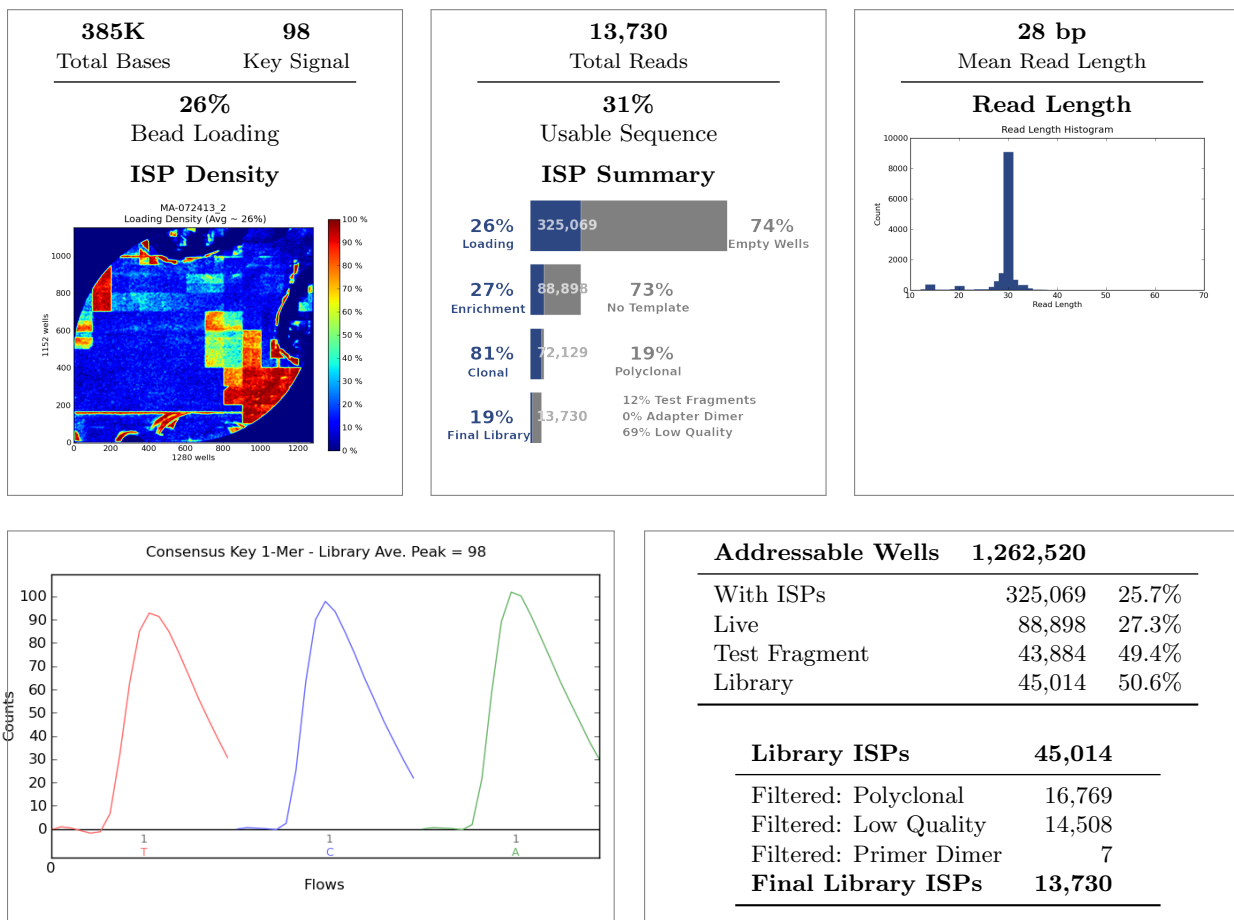

Test Fragment

Reads

Percent 50AQ17

Read Length Histogram

TF\_A

8,157

86%

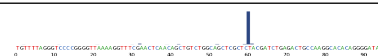

Supplement: Data S1 — Raw sequencing data files for presented experiments. The compressed file contains a table of content for all files included (0. TOC Supporting Data Files.txt) and raw sequencing data (PGM Torrent Suite run reports, FASTQ files and Sphix generated CSV files) for experiments presented in Figure 2 and Figure 3 (same data set as Figure S1). (ZIP) [file pone.0076696.s001.zip › Supporting Data Files/2. Figure 3 and S1/2.1 Figure 3 and S1 files/2.1 TS Reports/12_OM-1848_TS-342.pdf]

## Run Summary

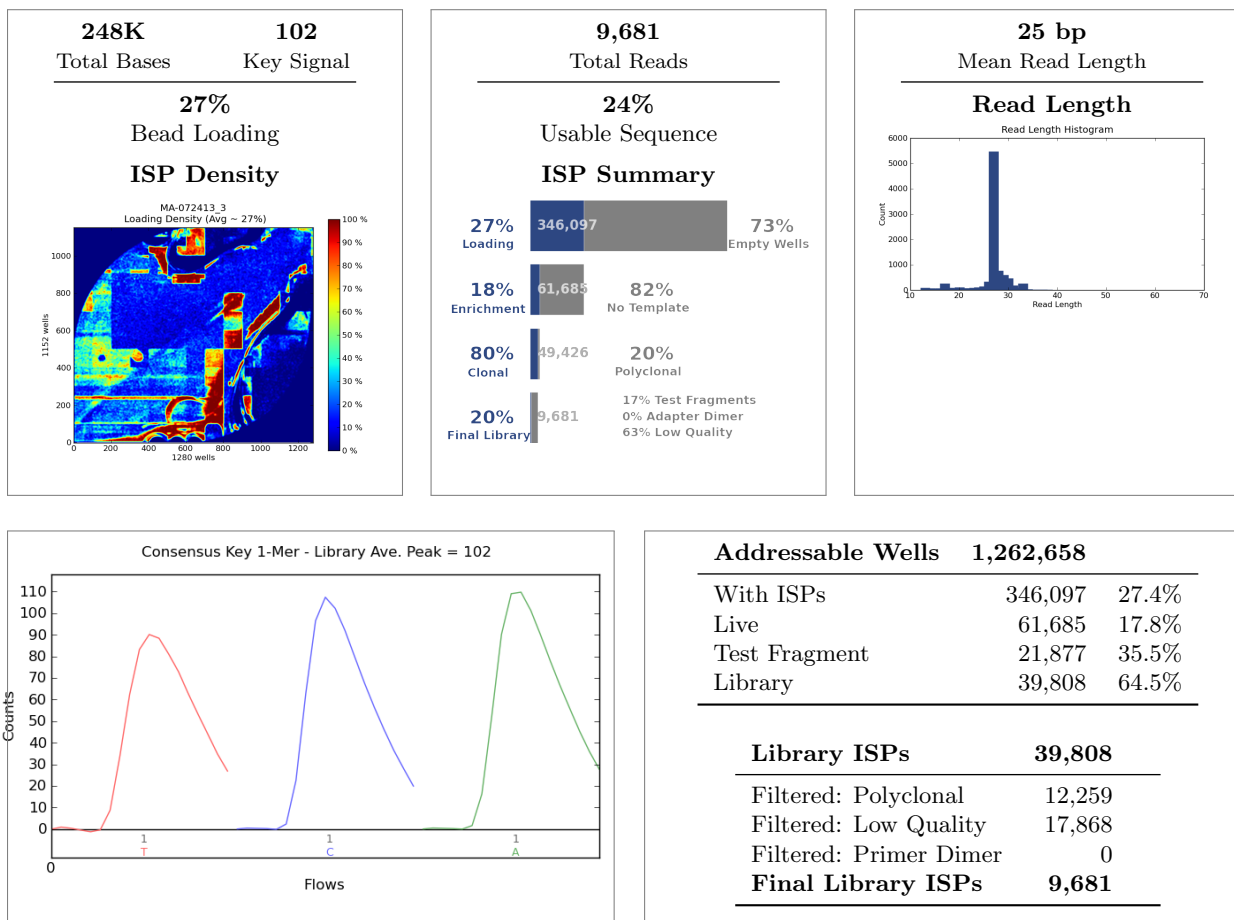

Test Fragment

Reads

Percent 50AQ17

Read Length Histogram

TF\_A

7,542

68%

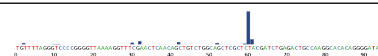

Supplement: Data S1 — Raw sequencing data files for presented experiments. The compressed file contains a table of content for all files included (0. TOC Supporting Data Files.txt) and raw sequencing data (PGM Torrent Suite run reports, FASTQ files and Sphix generated CSV files) for experiments presented in Figure 2 and Figure 3 (same data set as Figure S1). (ZIP) [file pone.0076696.s001.zip › Supporting Data Files/2. Figure 3 and S1/2.1 Figure 3 and S1 files/2.1 TS Reports/13_OM-1854_TS-342.pdf]

## Run Summary

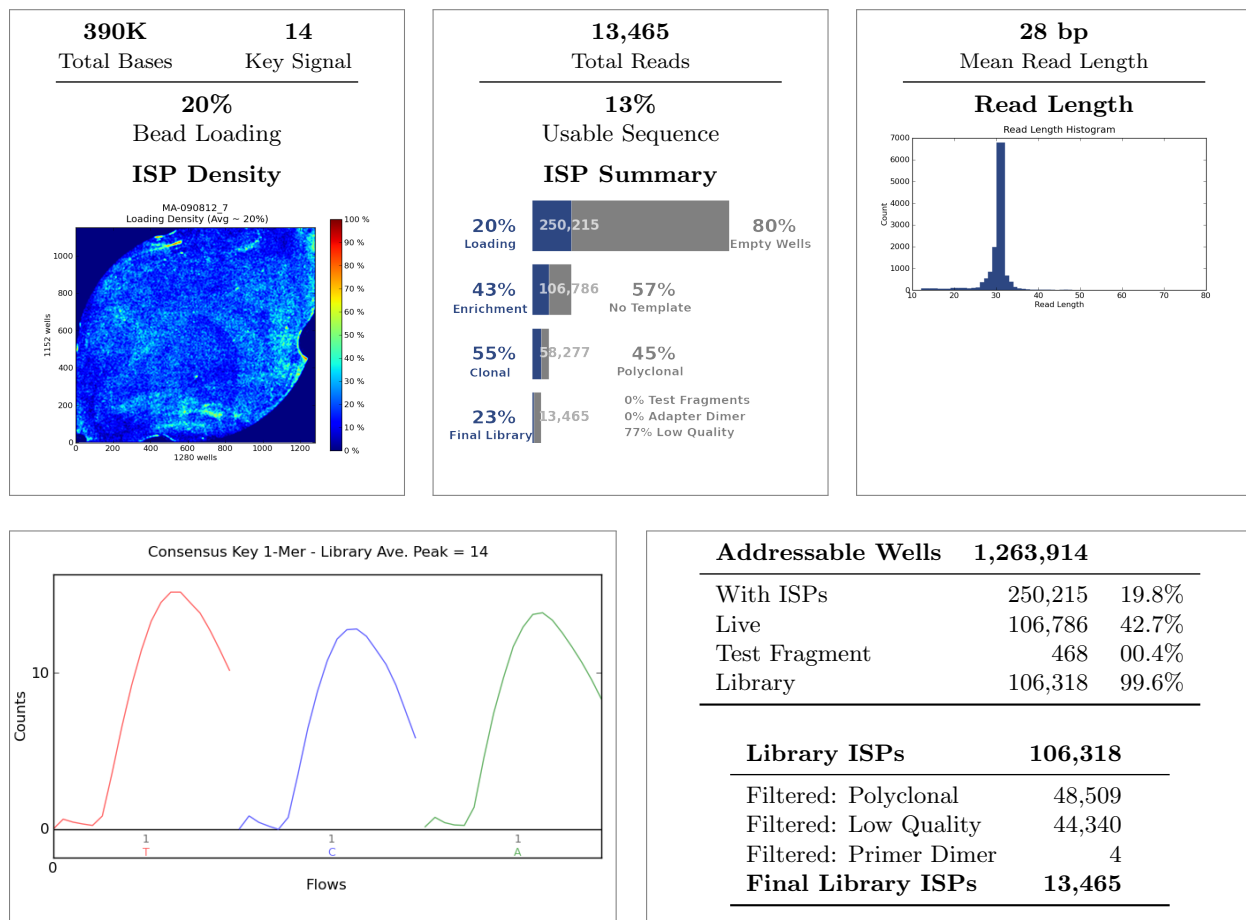

Supplement: Data S1 — Raw sequencing data files for presented experiments. The compressed file contains a table of content for all files included (0. TOC Supporting Data Files.txt) and raw sequencing data (PGM Torrent Suite run reports, FASTQ files and Sphix generated CSV files) for experiments presented in Figure 2 and Figure 3 (same data set as Figure S1). (ZIP) [file pone.0076696.s001.zip › Supporting Data Files/2. Figure 3 and S1/2.1 Figure 3 and S1 files/2.1 TS Reports/14_OM-1967_TS-342.pdf]

## Run Summary

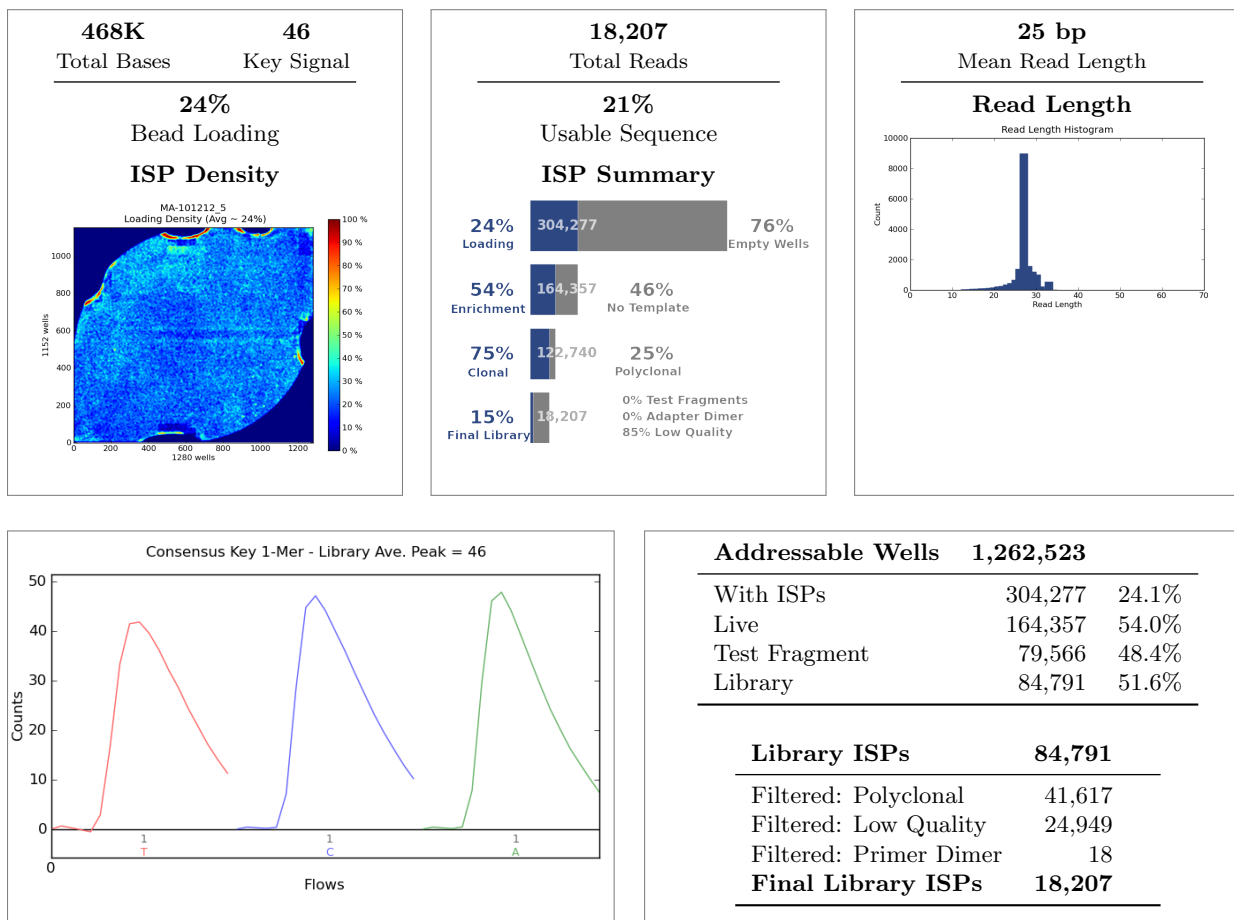

Supplement: Data S1 — Raw sequencing data files for presented experiments. The compressed file contains a table of content for all files included (0. TOC Supporting Data Files.txt) and raw sequencing data (PGM Torrent Suite run reports, FASTQ files and Sphix generated CSV files) for experiments presented in Figure 2 and Figure 3 (same data set as Figure S1). (ZIP) [file pone.0076696.s001.zip › Supporting Data Files/2. Figure 3 and S1/2.1 Figure 3 and S1 files/2.1 TS Reports/15_OM-1980_TS-342.pdf]

## Run Summary

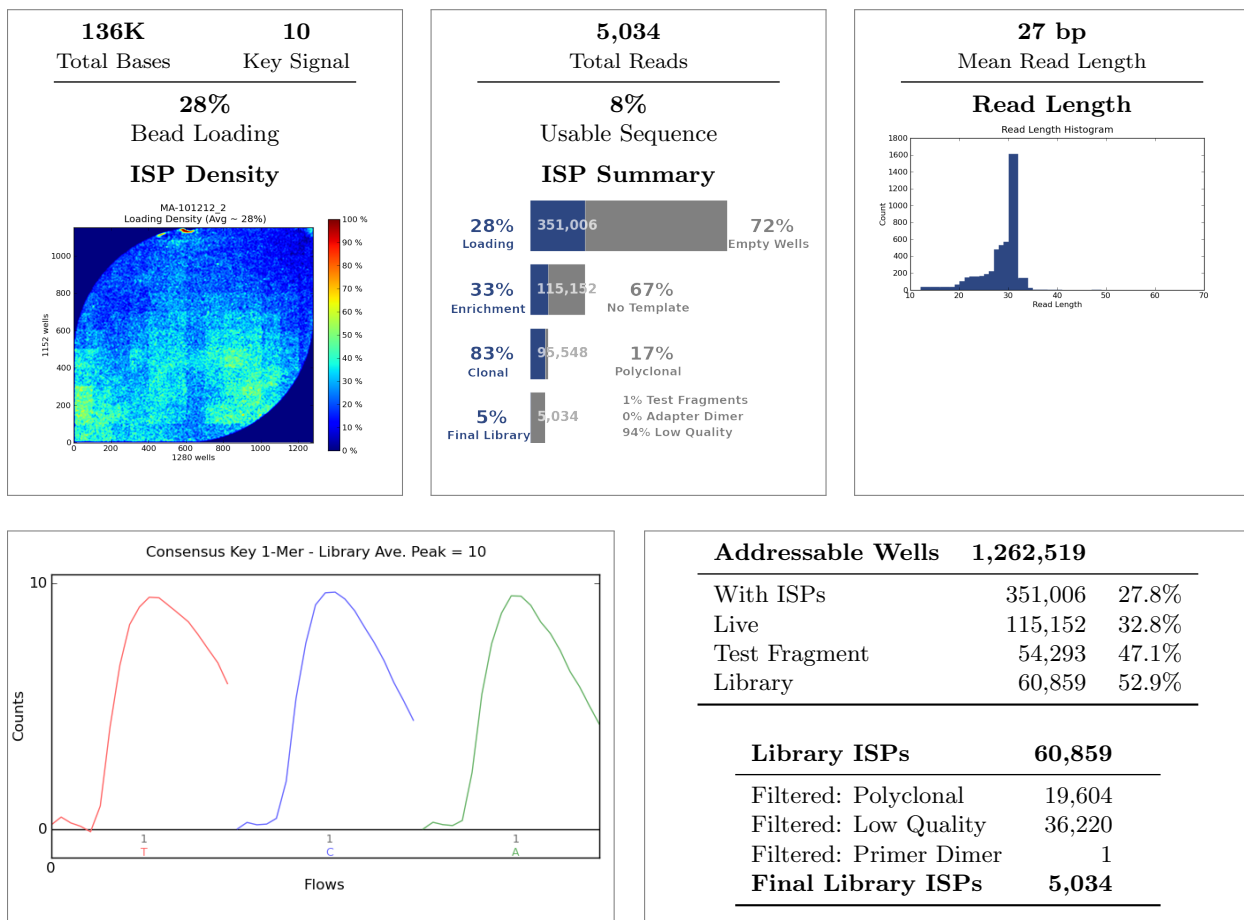

Supplement: Data S1 — Raw sequencing data files for presented experiments. The compressed file contains a table of content for all files included (0. TOC Supporting Data Files.txt) and raw sequencing data (PGM Torrent Suite run reports, FASTQ files and Sphix generated CSV files) for experiments presented in Figure 2 and Figure 3 (same data set as Figure S1). (ZIP) [file pone.0076696.s001.zip › Supporting Data Files/2. Figure 3 and S1/2.1 Figure 3 and S1 files/2.1 TS Reports/17_OM-2059_TS-342.pdf]

## Run Summary

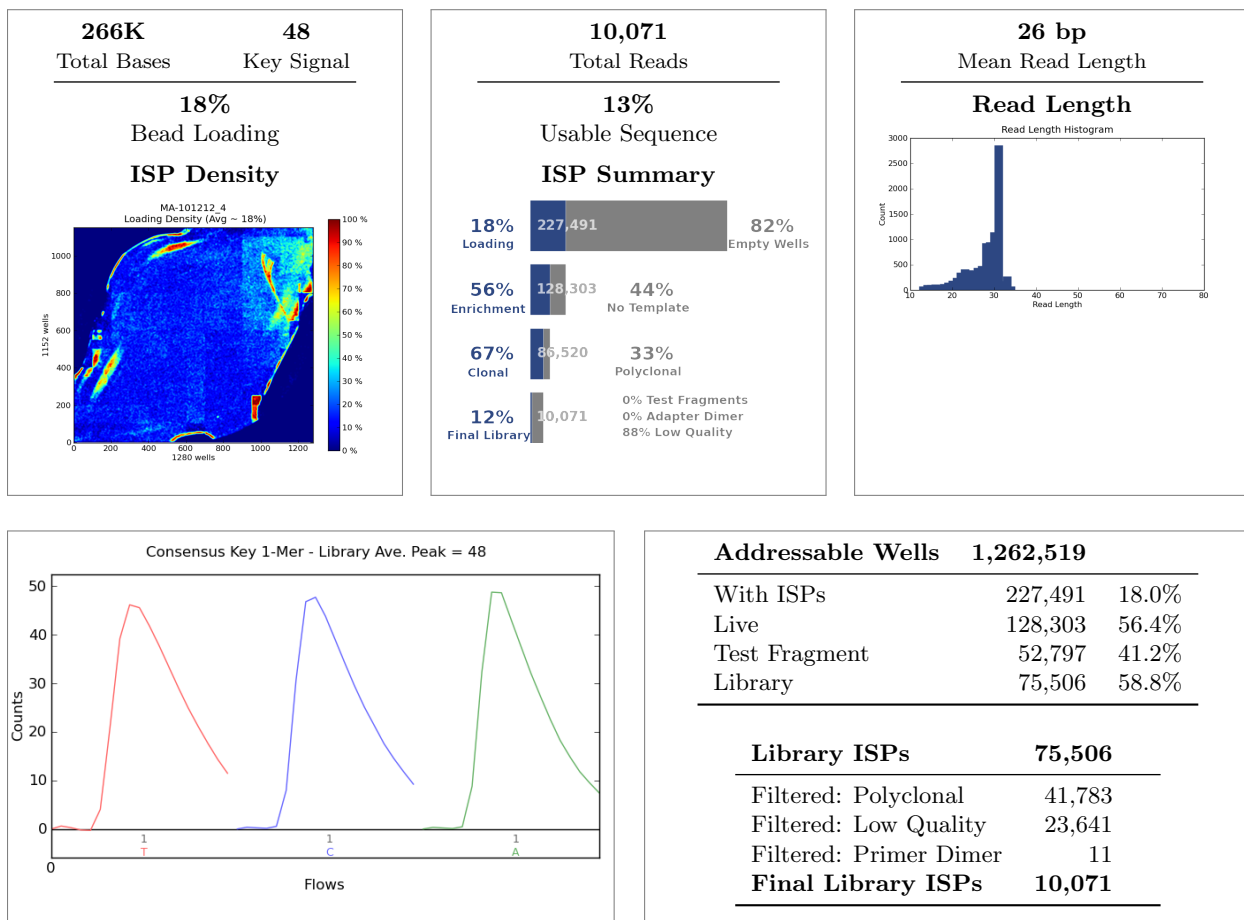

Supplement: Data S1 — Raw sequencing data files for presented experiments. The compressed file contains a table of content for all files included (0. TOC Supporting Data Files.txt) and raw sequencing data (PGM Torrent Suite run reports, FASTQ files and Sphix generated CSV files) for experiments presented in Figure 2 and Figure 3 (same data set as Figure S1). (ZIP) [file pone.0076696.s001.zip › Supporting Data Files/2. Figure 3 and S1/2.1 Figure 3 and S1 files/2.1 TS Reports/19_OM-2257_TS-342.pdf]

## Run Summary

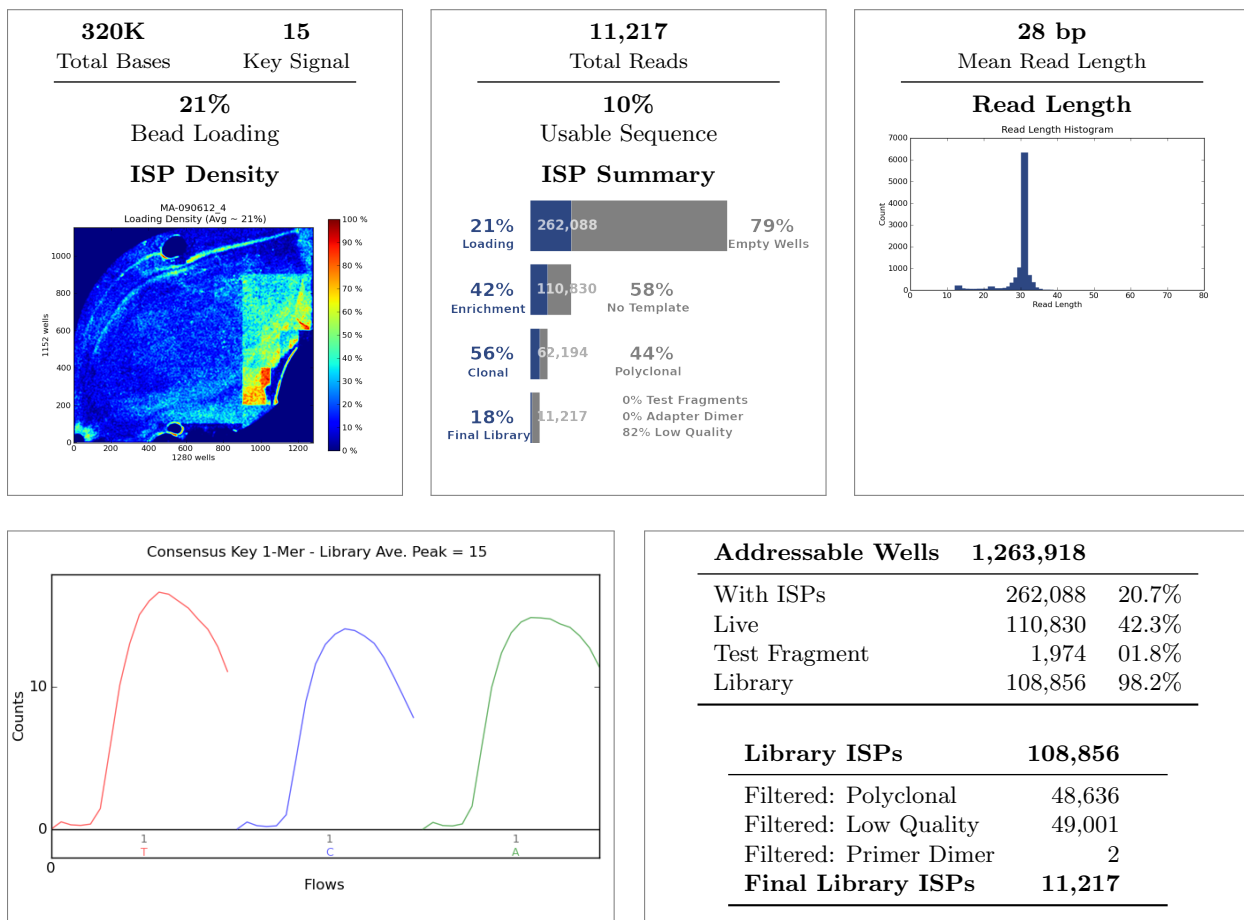

Supplement: Data S1 — Raw sequencing data files for presented experiments. The compressed file contains a table of content for all files included (0. TOC Supporting Data Files.txt) and raw sequencing data (PGM Torrent Suite run reports, FASTQ files and Sphix generated CSV files) for experiments presented in Figure 2 and Figure 3 (same data set as Figure S1). (ZIP) [file pone.0076696.s001.zip › Supporting Data Files/2. Figure 3 and S1/2.1 Figure 3 and S1 files/2.1 TS Reports/1_OM-1078_TS-342.pdf]

## Run Summary

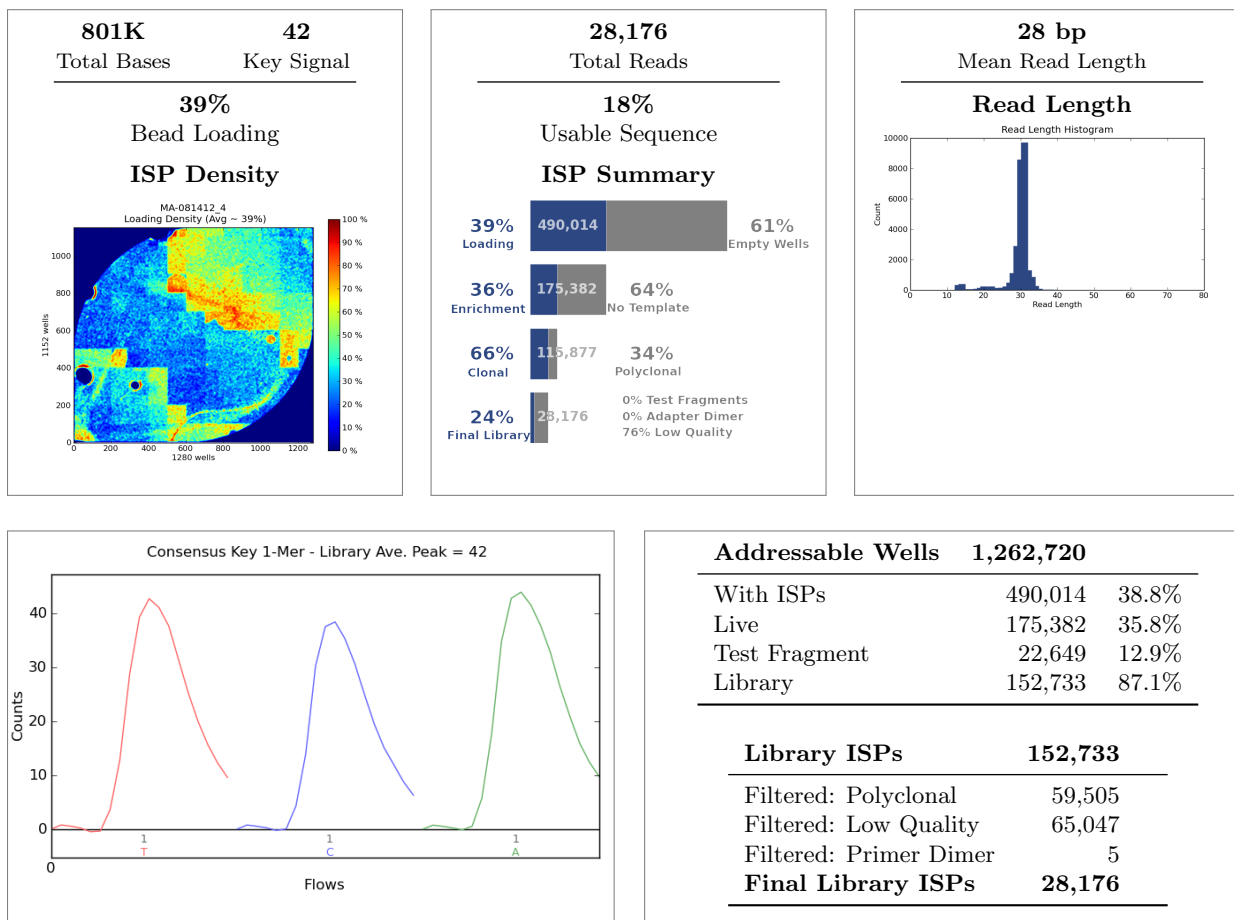

Supplement: Data S1 — Raw sequencing data files for presented experiments. The compressed file contains a table of content for all files included (0. TOC Supporting Data Files.txt) and raw sequencing data (PGM Torrent Suite run reports, FASTQ files and Sphix generated CSV files) for experiments presented in Figure 2 and Figure 3 (same data set as Figure S1). (ZIP) [file pone.0076696.s001.zip › Supporting Data Files/2. Figure 3 and S1/2.1 Figure 3 and S1 files/2.1 TS Reports/20_OM-2258_TS-342.pdf]

## Run Summary

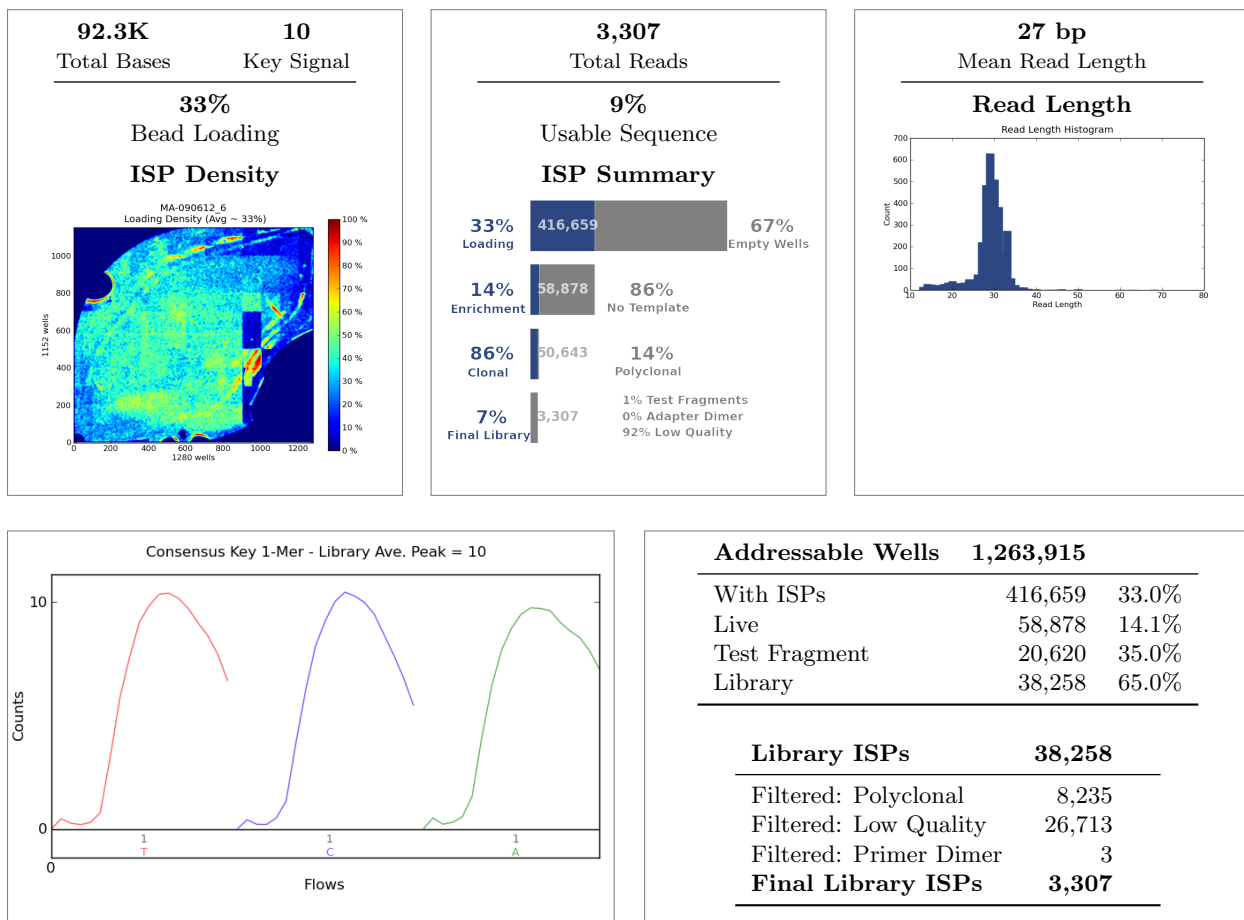

Supplement: Data S1 — Raw sequencing data files for presented experiments. The compressed file contains a table of content for all files included (0. TOC Supporting Data Files.txt) and raw sequencing data (PGM Torrent Suite run reports, FASTQ files and Sphix generated CSV files) for experiments presented in Figure 2 and Figure 3 (same data set as Figure S1). (ZIP) [file pone.0076696.s001.zip › Supporting Data Files/2. Figure 3 and S1/2.1 Figure 3 and S1 files/2.1 TS Reports/3_OM-1299_TS-342.pdf]

## Run Summary

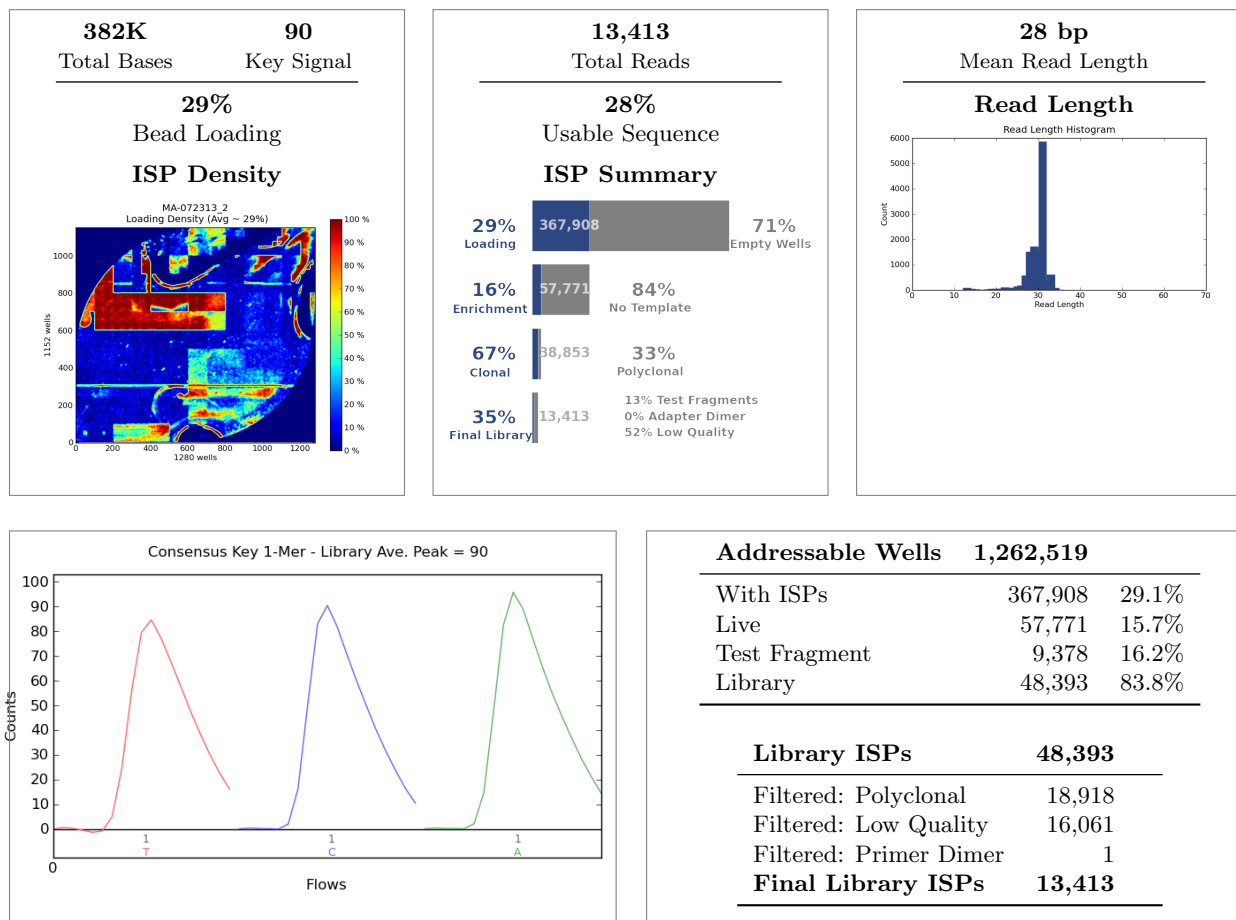

Test Fragment

Reads

Percent 50AQ17

Read Length Histogram

TF\_A

4,817

86%

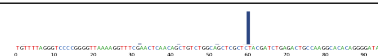

Supplement: Data S1 — Raw sequencing data files for presented experiments. The compressed file contains a table of content for all files included (0. TOC Supporting Data Files.txt) and raw sequencing data (PGM Torrent Suite run reports, FASTQ files and Sphix generated CSV files) for experiments presented in Figure 2 and Figure 3 (same data set as Figure S1). (ZIP) [file pone.0076696.s001.zip › Supporting Data Files/2. Figure 3 and S1/2.1 Figure 3 and S1 files/2.1 TS Reports/4_OM-1301_TS-342.pdf]

## Run Summary

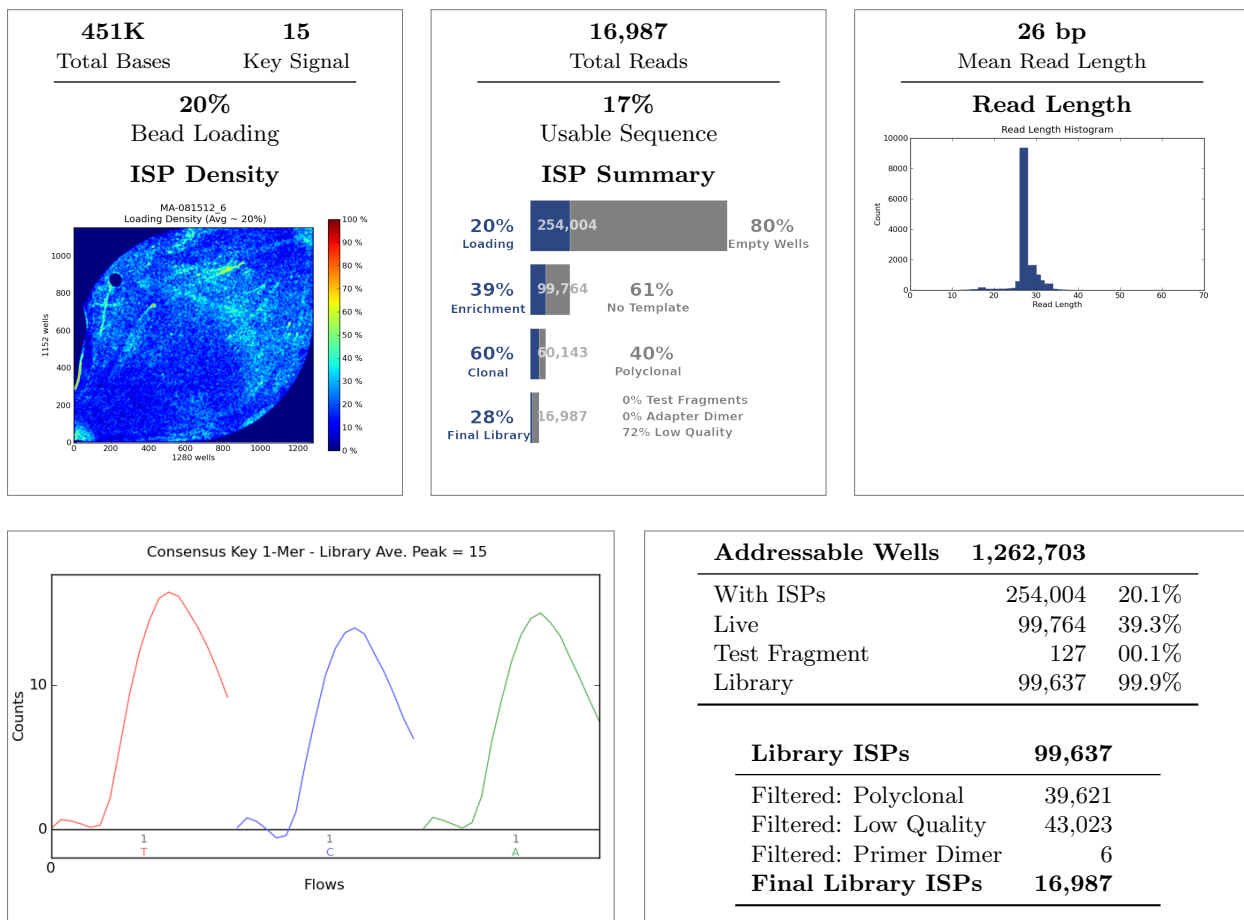

Supplement: Data S1 — Raw sequencing data files for presented experiments. The compressed file contains a table of content for all files included (0. TOC Supporting Data Files.txt) and raw sequencing data (PGM Torrent Suite run reports, FASTQ files and Sphix generated CSV files) for experiments presented in Figure 2 and Figure 3 (same data set as Figure S1). (ZIP) [file pone.0076696.s001.zip › Supporting Data Files/2. Figure 3 and S1/2.1 Figure 3 and S1 files/2.1 TS Reports/5_OM-1452_TS-342.pdf]

## Run Summary

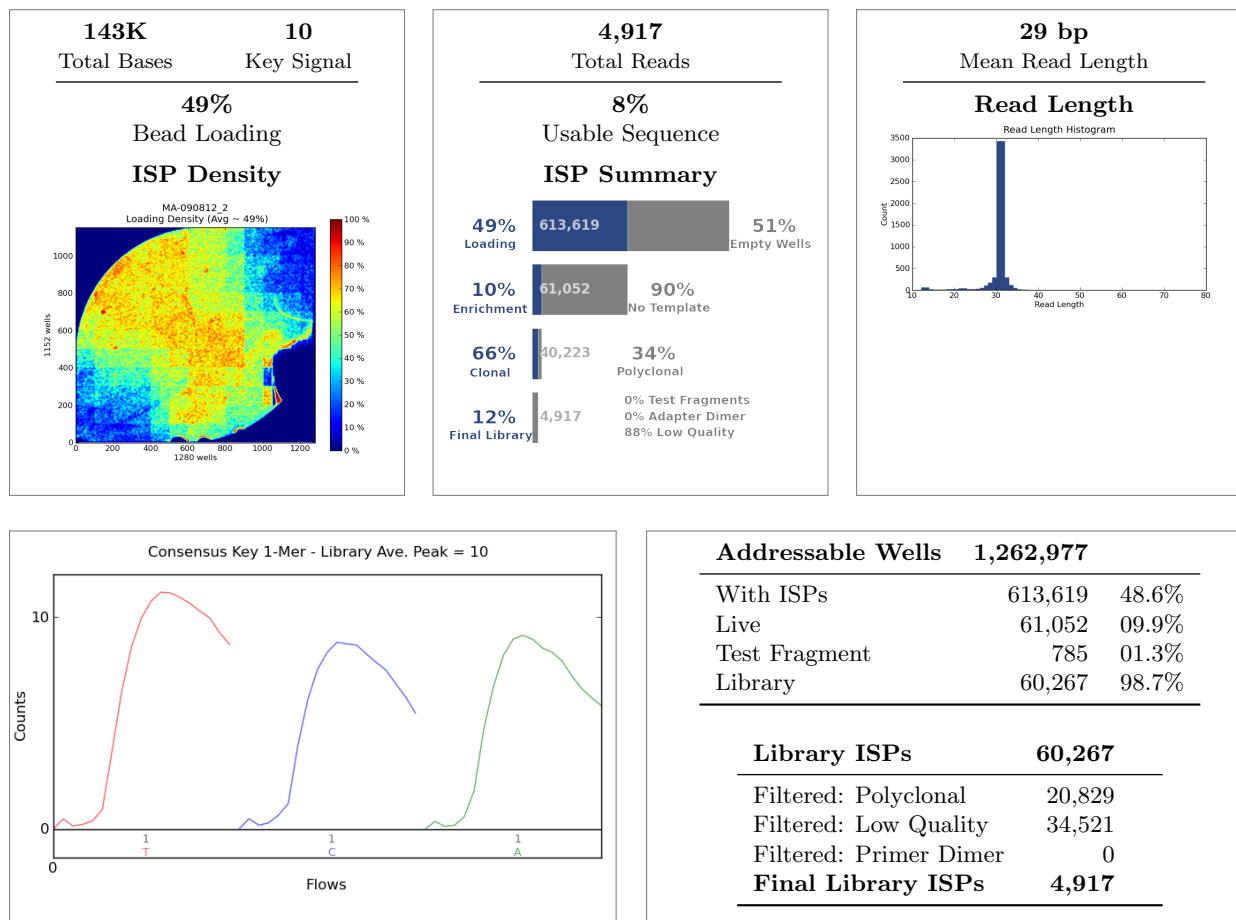

Supplement: Data S1 — Raw sequencing data files for presented experiments. The compressed file contains a table of content for all files included (0. TOC Supporting Data Files.txt) and raw sequencing data (PGM Torrent Suite run reports, FASTQ files and Sphix generated CSV files) for experiments presented in Figure 2 and Figure 3 (same data set as Figure S1). (ZIP) [file pone.0076696.s001.zip › Supporting Data Files/2. Figure 3 and S1/2.1 Figure 3 and S1 files/2.1 TS Reports/6_OM-1464_TS-342.pdf]

## Run Summary

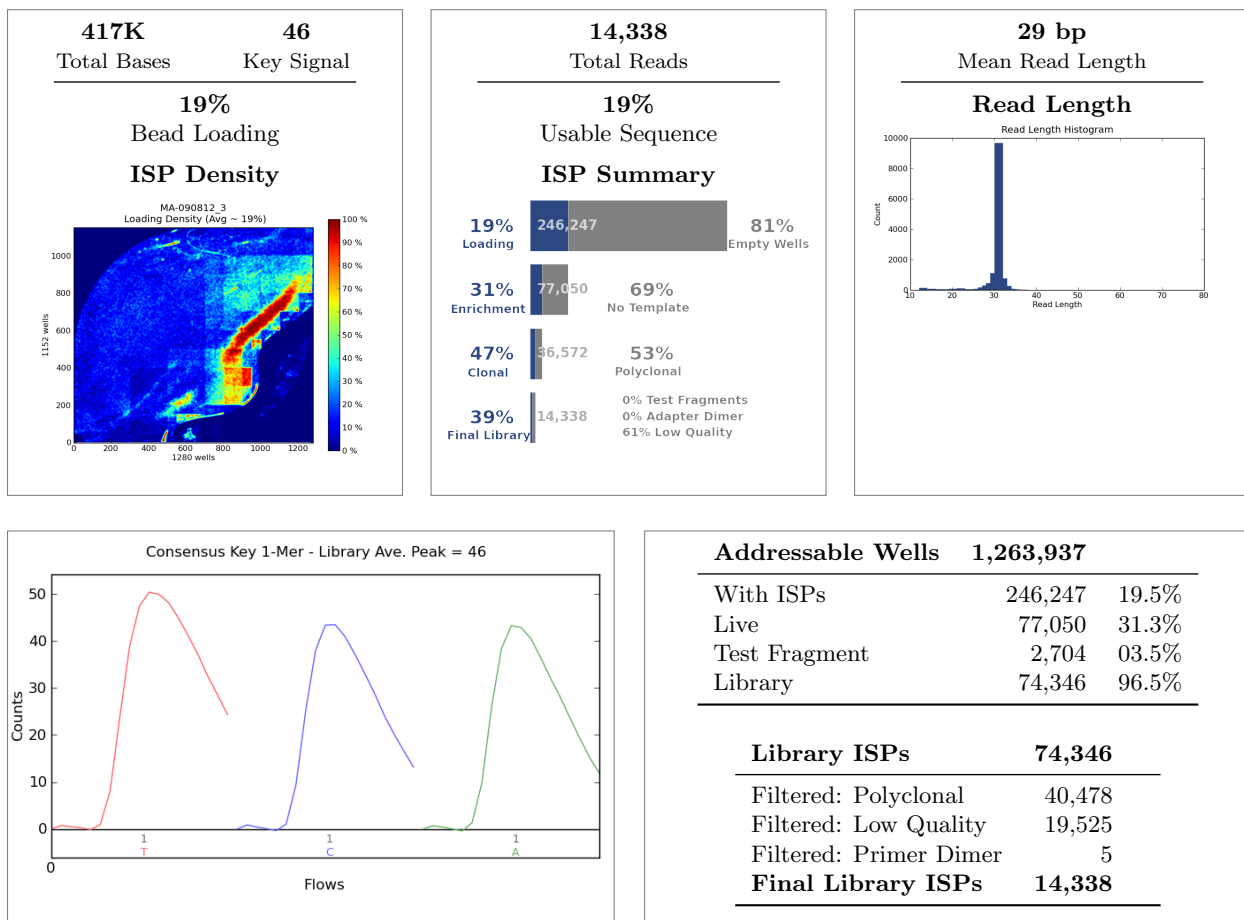

Supplement: Data S1 — Raw sequencing data files for presented experiments. The compressed file contains a table of content for all files included (0. TOC Supporting Data Files.txt) and raw sequencing data (PGM Torrent Suite run reports, FASTQ files and Sphix generated CSV files) for experiments presented in Figure 2 and Figure 3 (same data set as Figure S1). (ZIP) [file pone.0076696.s001.zip › Supporting Data Files/2. Figure 3 and S1/2.1 Figure 3 and S1 files/2.1 TS Reports/7_OM-1530_TS-342.pdf]

## Run Summary

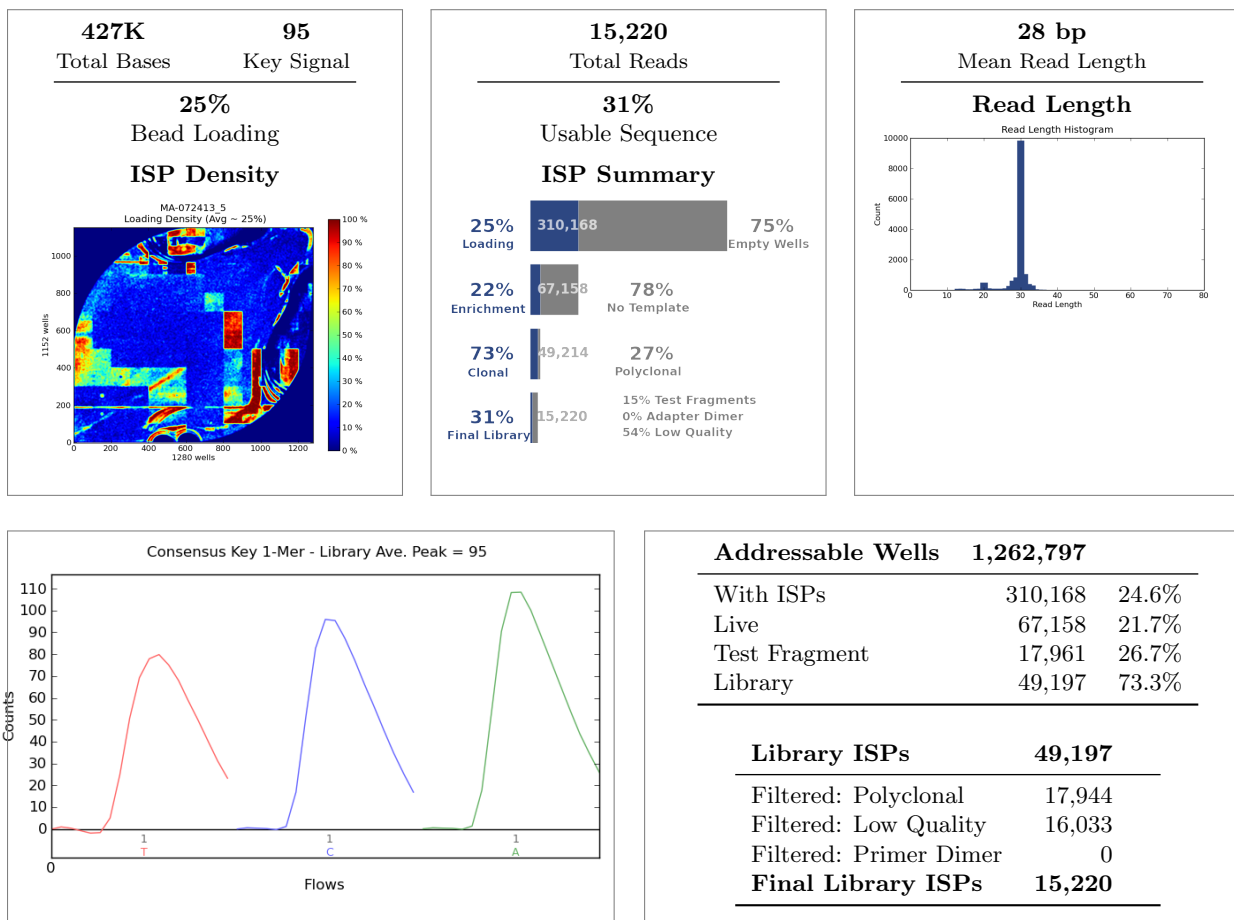

Test Fragment

Reads

Percent 50AQ17

Read Length Histogram

TF\_A

7,007

77%

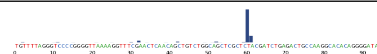

Supplement: Data S1 — Raw sequencing data files for presented experiments. The compressed file contains a table of content for all files included (0. TOC Supporting Data Files.txt) and raw sequencing data (PGM Torrent Suite run reports, FASTQ files and Sphix generated CSV files) for experiments presented in Figure 2 and Figure 3 (same data set as Figure S1). (ZIP) [file pone.0076696.s001.zip › Supporting Data Files/2. Figure 3 and S1/2.1 Figure 3 and S1 files/2.1 TS Reports/8_OM-1569_TS-342.pdf]

## Run Summary

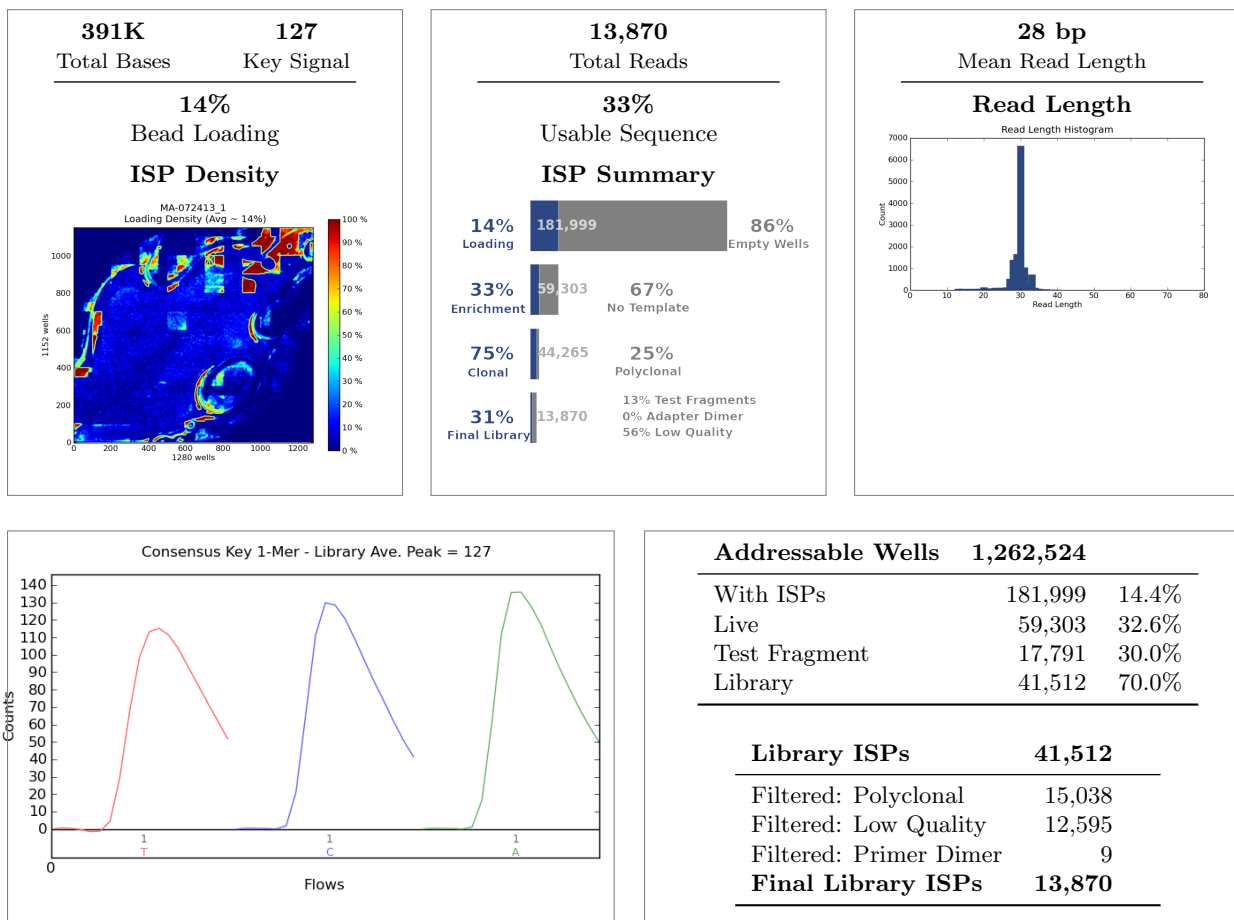

Test Fragment

Reads

Percent 50AQ17

Read Length Histogram

TF\_A

5,059

75%

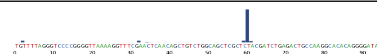

Supplement: Data S1 — Raw sequencing data files for presented experiments. The compressed file contains a table of content for all files included (0. TOC Supporting Data Files.txt) and raw sequencing data (PGM Torrent Suite run reports, FASTQ files and Sphix generated CSV files) for experiments presented in Figure 2 and Figure 3 (same data set as Figure S1). (ZIP) [file pone.0076696.s001.zip › Supporting Data Files/2. Figure 3 and S1/2.1 Figure 3 and S1 files/2.1 TS Reports/9_OM-1668_TS-342.pdf]

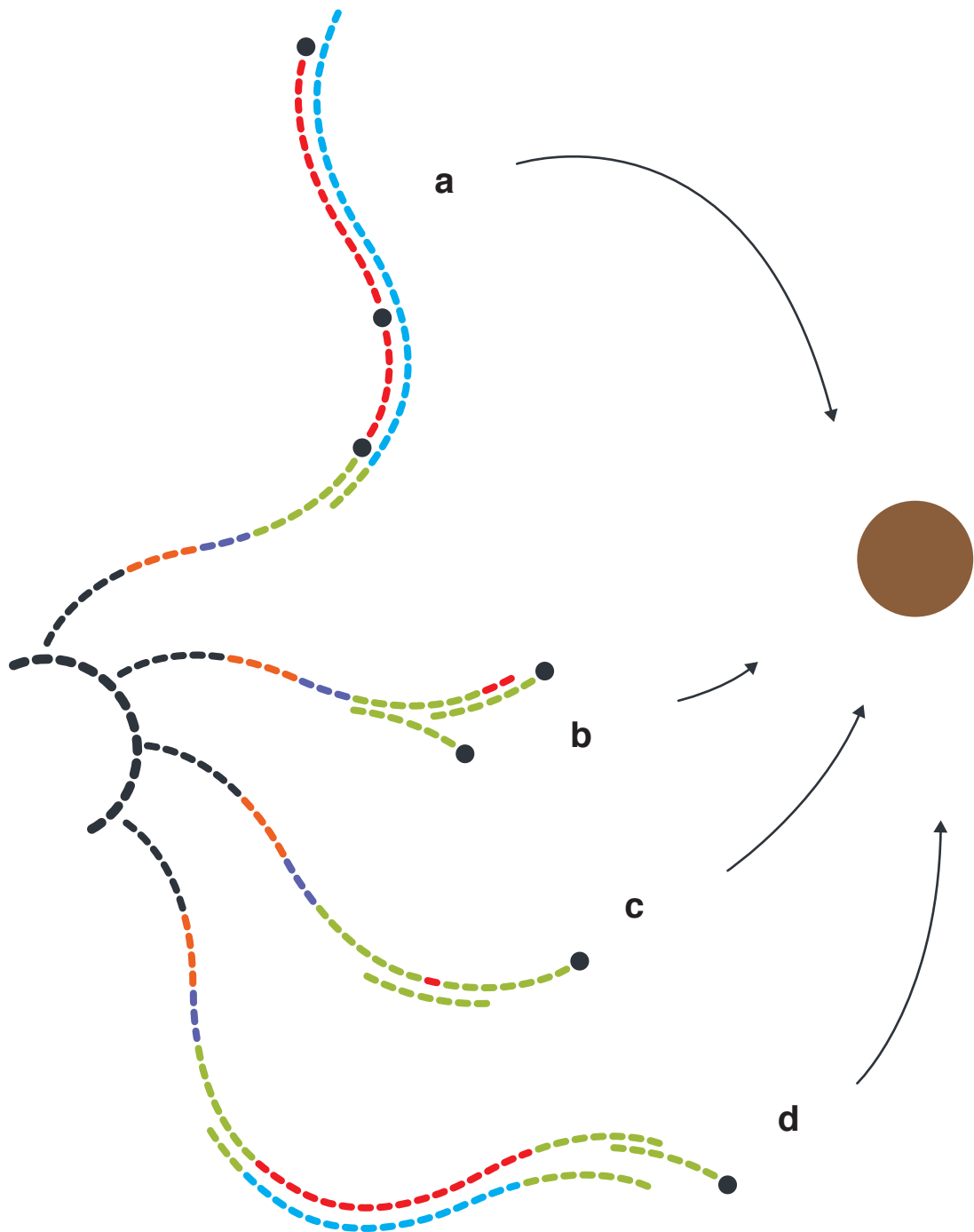

Supplement: Figure S2 — Conceptual illustration of alternative reporter activation strategies. A reporter library construction method in which synthetic oligonucleotides are directly coupled to the beads would allow for alternative reporter activation strategies. In contrast to the scheme outlined in Figure 1, the proposed cluster of synthetic oligonucleotides viewed from the 5´-end would instead consist of: i) a general linker site for bead coupling (black), ii) a target unique ID trace (orange), iii) a general priming site (purple), and iv) a target hybridization site (green). Biotin molecules are depicted as solid black dots that will bind to a streptavidin coated solid surface, here depicted as a paramagnetic bead (brown), for downstream separation. (a) Selectively hybridizing a complementary target (or tag) to a bead-clustered oligonucleotide at the 3´-end allows for a polymerization reaction using biotinylated nucleotides. This creates covalently bonded biotin molecules and thus possibly enables a stronger selection, and furthermore allows for single-base interrogations, e.g. single nucleotide polymorphisms (SNPs) detection. If the target template is longer (blue) the polymerization could continue further generating a longer complementary strand (red) with multiple biotin reporter activating molecules (black dots), given that a fraction of the dNTPs in the reaction are biotinylated. (b) Selectively hybridizing a biotinylated complementary target (or tag) to a bead-clustered oligonucleotide at the 3´-end could possibly ease steric hindrance. Also, by including multiple target hybridization sites (replicates or different targets), the process can be made more sensitive, with the possibility of binding multiple-targets (here represented as two duplicates). This strategy could be combined with a polymerase extension of the bead-clustered oligonucleotide at the 3´-end, generating a complementary strand (red), thus enabling a stronger selection basis. (c) Selectively hybridizing a compleme [file pone.0076696.s003.pdf]

**a**

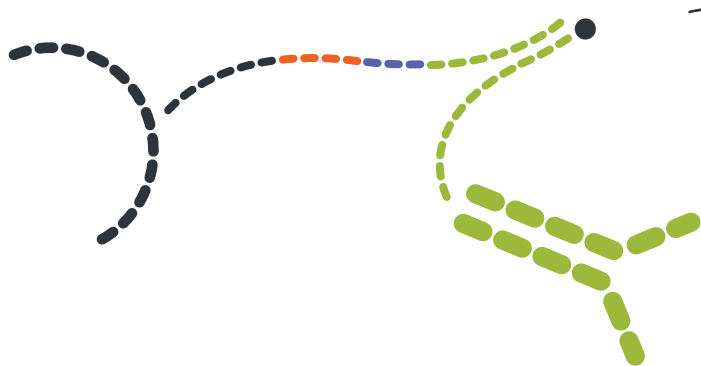

**b**

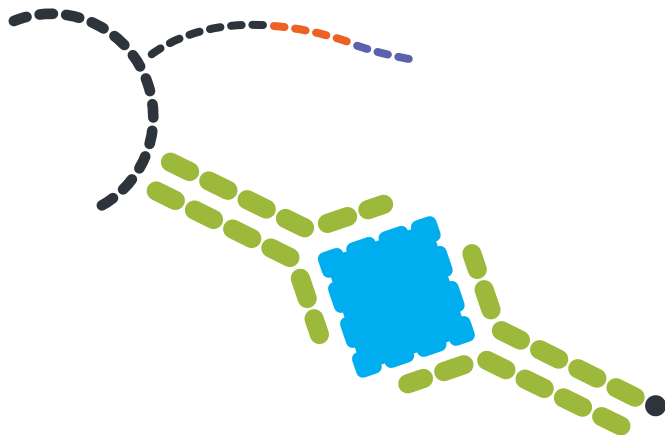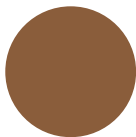

Supplement: Figure S3 — Conceptual illustration of immunoassay reporter activation strategies. A reporter library construction method in which synthetic oligonucleotides and other biomolecules (e.g. antibodies) are directly coupled to the beads. This would allow for alternative reporter activation strategies, and targeting other biomolecules than nucleic acids. Biotin molecules are depicted as solid black dots that will bind to a streptavidin coated solid surface, here depicted as a paramagnetic bead (brown), for downstream separation. (a) A cluster of synthetic oligonucleotides viewed from the 5´-end consisting of: i) a general linker site for bead coupling (black), ii) a target unique ID trace (orange), iii) a general priming site (purple), and iv) a target hybridization site (green). The oligonucleotides could also be oriented as described in Figure 1. In contrast to previous versions the oligonucleotide-target or tag is conjugated to antibody-reporter, as used in e.g. the proximity ligation assay (PLA) for immunoassays [39,41-43]. Reporter activation in this case can be any of the previously described strategies (Figure 1 and Figure S2). (b) A reporter containing both bead-clustered oligonucleotides for signal amplification, and bead-conjugated antibodies (or other biomarkers) (green) for immunoassay target reporter activation and selection. The clustered oligonucleotides would in this case contain i) a general linker site for bead coupling (black), ii) a target unique ID trace (orange), and iii) a general priming site (purple). The reporters are activated and selected via an antibody-antigen-antibody complex (antigen in blue). A combinatorial assay targeting both genomic and proteomic markers could thus be envisioned [44]. (PDF) [file pone.0076696.s004.pdf]
