# Supplementary material for: The Sequencing Bead Array (SBA), a Next-Generation Digital Suspension Array
Source: PLoS One. 2013 Oct 7;8(10):e76696. doi: 10.1371/journal.pone.0076696 (PMC3792038; doi:10.1371/journal.pone.0076696)

OM-1078

Reporter population:	108856	
Polyclonality:	48636	(44.68%)
Low quality:	49003	(45.02%)
Uncalled reporters:	1239	(1.14%)
Called reporters:	9978	(9.17%)

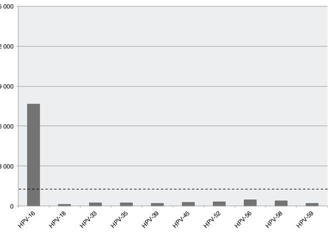

OM-1272

Reporter population:	115970	
Polyclonality:	53385	(46.03%)
Low quality:	47942	(41.34%)
Uncalled reporters:	1530	(1.32%)
Called reporters:	13113	(11.31%)

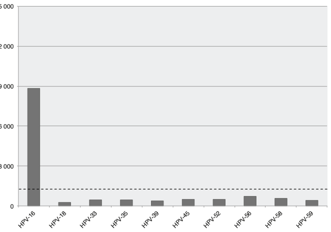

OM-1299

Reporter population:	38258	
Polyclonality:	8235	(21.52%)
Low quality:	26716	(69.83%)
Uncalled reporters:	328	(0.86%)
Called reporters:	2979	(7.79%)

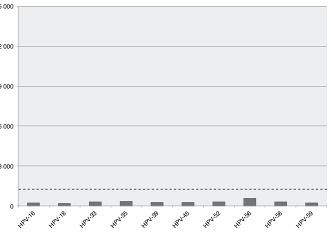

OM-1301

Reporter population:	48393	
Polyclonality:	18918	(39.09%)
Low quality:	16062	(33.19%)
Uncalled reporters:	719	(1.49%)
Called reporters:	12694	(26.23%)

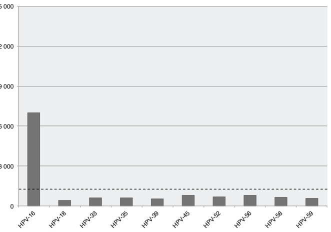

OM-1452

Reporter population:	99637	
Polyclonality:	39621	(39.77%)
Low quality:	43029	(43.19%)
Uncalled reporters:	1067	(1.07%)
Called reporters:	15920	(15.98%)

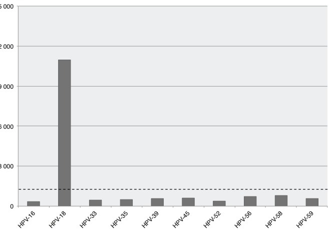

OM-1464

Reporter population:	60267	
Polyclonality:	20829	(34.56%)
Low quality:	34521	(57.28%)
Uncalled reporters:	438	(0.73%)
Called reporters:	4479	(7.43%)

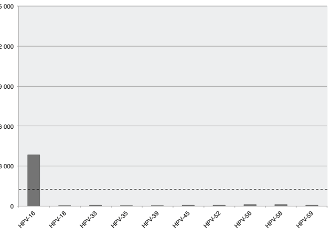

OM-1530

Reporter population:	74346	
Polyclonality:	40478	(54.45%)
Low quality:	19530	(26.27%)
Uncalled reporters:	1422	(1.91%)
Called reporters:	12916	(17.37%)

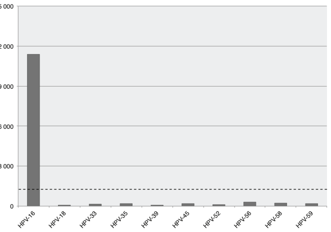

OM-1569

Reporter population:	49197	
Polyclonality:	17944	(36.47%)
Low quality:	16033	(32.59%)
Uncalled reporters:	613	(1.25%)
Called reporters:	14607	(29.69%)

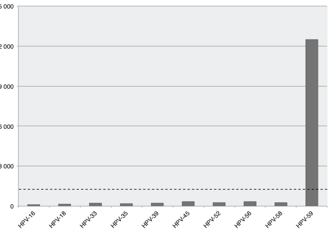

OM-1668

Reporter population:	41512	
Polyclonality:	15038	(36.23%)
Low quality:	12604	(30.36%)
Uncalled reporters:	743	(1.79%)
Called reporters:	13127	(31.62%)

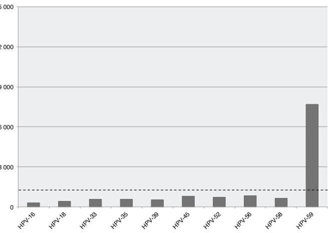

OM-1741

Reporter population:	66293	
Polyclonality:	32638	(49.23%)
Low quality:	25097	(37.86%)
Uncalled reporters:	725	(1.09%)
Called reporters:	7833	(11.82%)

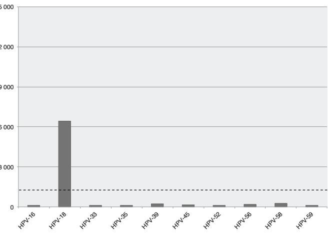

OM-1751

Reporter population:	20165	
Polyclonality:	7574	(37.56%)
Low quality:	7538	(37.38%)
Uncalled reporters:	288	(1.43%)
Called reporters:	4765	(23.63%)

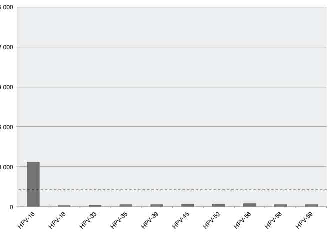

OM-1848

Reporter population:	45014	
Polyclonality:	16769	(37.25%)
Low quality:	14515	(32.25%)
Uncalled reporters:	437	(0.97%)
Called reporters:	13293	(29.53%)

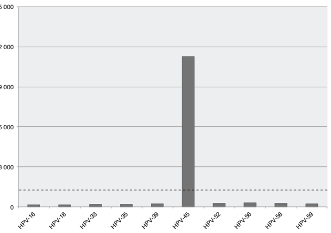

OM-1854

Reporter population:	39808	
Polyclonality:	12259	(30.80%)
Low quality:	17868	(44.89%)
Uncalled reporters:	562	(1.41%)
Called reporters:	9119	(22.91%)

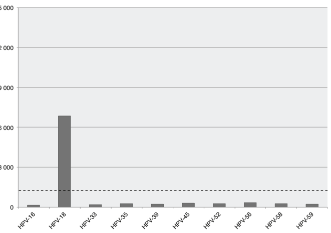

OM-1967

Reporter population:	106318	
Polyclonality:	48509	(45.63%)
Low quality:	44344	(41.71%)
Uncalled reporters:	1442	(1.36%)
Called reporters:	12023	(11.31%)

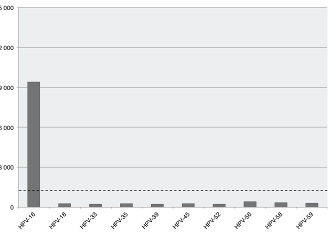

OM-1980

Reporter population:	84791	
Polyclonality:	41617	(49.08%)
Low quality:	24967	(29.45%)
Uncalled reporters:	964	(1.14%)
Called reporters:	17243	(20.34%)

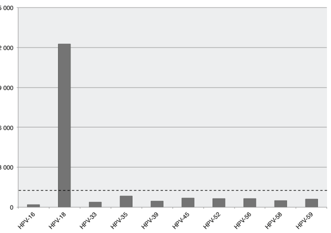

OM-2006

Reporter population:	70289	
Polyclonality:	28482	(40.52%)
Low quality:	35649	(50.72%)
Uncalled reporters:	478	(0.68%)
Called reporters:	5680	(8.08%)

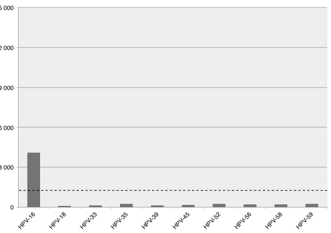

OM-2059

Reporter population:	60859	
Polyclonality:	19604	(32.21%)
Low quality:	36221	(59.52%)
Uncalled reporters:	417	(0.69%)
Called reporters:	4617	(7.59%)

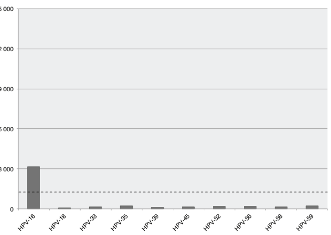

OM-2215

Reporter population:	120768	
Polyclonality:	64281	(53.23%)
Low quality:	34956	(28.94%)
Uncalled reporters:	1277	(1.06%)
Called reporters:	20254	(16.77%)

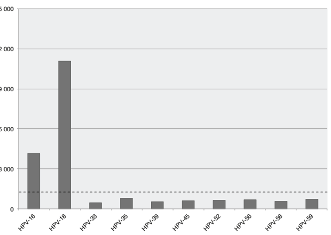

OM-2257

Reporter population:	75506	
Polyclonality:	41783	(55.34%)
Low quality:	23652	(31.32%)
Uncalled reporters:	890	(1.18%)
Called reporters:	9181	(12.16%)

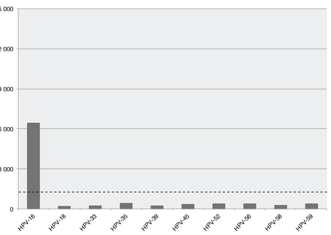

OM-2258

Reporter population:	152733	
Polyclonality:	59505	(38.96%)
Low quality:	65052	(42.59%)
Uncalled reporters:	1348	(0.88%)
Called reporters:	26828	(17.57%)

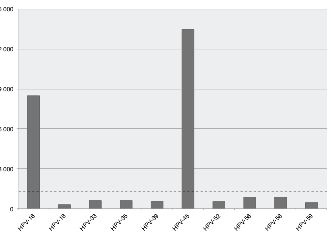

Supplement: Figure S1 — Bar-histograms for 20 cervical tumor samples (extracted genomic DNA). The reporter bars in the bar-histograms are represented with y-axis read counts (metadata in Table S5). Positive genotype calls reach beyond the included threshold (dashed line), while calls below are within background noise variation. The y-axis is scaled to a maximum of 15,000 reads in all of the graphs to allow for side-by-side comparison. Co-infections were observed in samples OM-2215 and OM-2258. OM-1299 was negative for all ten investigated HPV genotypes, i.e. no peak signal was distinguishable from background noise. Metadata for each sample-sequencing run is also included (upper right corners) for reporter population, polyclonality, low quality, uncalled reporters, and called reporters (defined in Table 1). Results were called as: ten single infections for HPV-16, four single infections for HPV-18, two single infections for HPV-59, one single infection for HPV-45, one dual co-infection for HPV-16, and HPV-18, one dual co-infection for HPV-16, and HPV-45, and one genotype-negative sample. (PDF) [file pone.0076696.s002.pdf]
