# Supplementary material for: The Sequencing Bead Array (SBA), a Next-Generation Digital Suspension Array
Source: PLoS One. 2013 Oct 7;8(10):e76696. doi: 10.1371/journal.pone.0076696 (PMC3792038; doi:10.1371/journal.pone.0076696)
Supplement: Table S1 — The Sequencing Bead Array (SBA) reporter oligonucleotide templates. Oligonucleotide templates for emulsion PCR reporter library construction. All oligonucleotides are presented in the 5´ to 3´ direction. The upper half of the table represents the oligonucleotides used for the described 10-plex HPV SBA assay, while the lower half details a list of 153 Multiplex Identifiers (MIDs) which could be used as ID traces for future SBA assay embodiments. The upper half table (white) is compartmentalized into five different sections. Sequence follows assembly of compartments, i.e. 5´-Priming site -Key - ID trace X -Target hybridization site X - Linker site -3´. Each oligonucleotide was synthesized as both the strand displayed in the table, and its reverse complementary strand to form duplex DNA templates. The priming site decodes where the sequence primer attaches. The Key is for PGM decoding and quality control purposes during the sequencing run. The ID trace is unique to each reporter and biomarker target. The target hybridization sites correspond to a genotype-specific sequence present in the L1 gene of the HPV-genome. The linker was used to clone the fragments onto the beads during the emulsion PCR step. The lower half table (grey) details a pre-defined library of 153 Multiplex Identifiers (MIDs) from 454 sequencing GS FLX Titanium chemistry, which provides an excellent resource for future ID traces (Roche Diagnostics 2009. Using multiplex identifier (MID) adaptors for the GS FLX titanium chemistry–extended MID set. Technical Bulletin: Genome Sequencer FLX System. TCB no. 005-2009. Roche, Branchburg, NJ.). (PDF) [file pone.0076696.s006.pdf]

**Sequence:**	**5´–Priming site – Key – ID trace X – Target hybridization site X – Linker site –3´**	
**Priming site:**	CCATCTCATCCCTGCGTGTCTCCGAC	
**Key:**	TCAG	
	**ID trace (MID #):**	**Target Hybridization site:**
**HPV-16**	ACGAGTGCGT (MID1)	TCCCCATGTCGTAGGTACTC
**HPV-18**	ACGCTCGACA (MID2)	TGCCCAGGTACAGGAGACTG
**HPV-33**	AGCACTGTAG (MID4)	CTGTCACTAGTTACTTGTGTGC
**HPV-35**	ATCAGACACG (MID5)	GACACAGCAGAACACACAGAC
**HPV-39**	ATATCGCGAG (MID6)	GGTATGGAAGACTCTATAGAGG
**HPV-45**	CGTGTCTCTA (MID7)	GGCACAGGATTTTGTGTAGAGGC
**HPV-52**	TAGTATCAGC (MID9)	CTCGCCATGACGAAGGTATTCC
**HPV-56**	TCTCTATGCG (MID10)	CTCCACATGTCTAAGGTACTG
**HPV-58**	TGATACGTCT (MID11)	CCTTCCTTAGTTACTTCAGTGC
**HPV-59**	TACTGAGCTA (MID12)	CACACACAGAAAGATTGGTGCTGCG
**Linker site:**	ATCACCGACTGCCCATAGAGAGGCTGAGACTGCCAAGGCACACAGGGGATAGG	

153 ID traces (MIDs):

Name:	Sequence:	Name:	Sequence:	Name:	Sequence:	Name:	Sequence:	Name:	Sequence:
MID1	ACGAGTGCGT	MID32	AGTACGCTAT	MID63	TAGTCGCATA	MID94	CGTATGCGAC	MID125	ATGTACGATG
MID2	ACGCTCGACA	MID33	ATAGAGTACT	MID64	TATATATACA	MID95	CGTCGATCTC	MID126	ATGTGTCTAG
MID3	AGACGCACTC	MID34	CACGCTACGT	MID65	TATGCTAGTA	MID96	CTACGACTGC	MID127	CACACGATAG
MID4	AGCACTGTAG	MID35	CAGTAGACGT	MID66	TCACGCGAGA	MID97	CTAGTCACTC	MID128	CACTCGCACG
MID5	ATCAGACACG	MID36	CGACGTGACT	MID67	TCGATAGTGA	MID98	CTCTACGCTC	MID129	CAGACGTCTG
MID6	ATATCGCGAG	MID37	TACACACACT	MID68	TCGCTGCGTA	MID99	CTGTACATAC	MID130	CAGTACTGCG
MID7	CGTGTCTCTA	MID38	TACACGTGAT	MID69	TCTGACGTCA	MID100	TAGACTGCAC	MID131	CGACAGCGAG
MID8	CTCGCGTGTC	MID39	TACAGATCGT	MID70	TGAGTCAGTA	MID101	TAGCGCGCGC	MID132	CGATCTGTCG
MID9	TAGTATCAGC	MID40	TACGCTGTCT	MID71	TGTAGTGTGA	MID102	TAGCTCTATC	MID133	CGCGTGCTAG
MID10	TCTCTATGCG	MID41	TAGTGTAGAT	MID72	TGTCACACGA	MID103	TATAGACATC	MID134	CGCTCGAGTG
MID11	TGATACGTCT	MID42	TCGATCACGT	MID73	TGTCGTCGCA	MID104	TATGATACGC	MID135	CGTGATGACG
MID12	TACTGAGCTA	MID43	TCGCACTAGT	MID74	ACACATACGC	MID105	TCACTCATAC	MID136	CTATGTACAG
MID13	CATAGTAGTG	MID44	TCTAGCGACT	MID75	ACAGTCGTGC	MID106	TCATCGAGTC	MID137	CTCGATATAG
MID14	CGAGAGATAC	MID45	TCTATACTAT	MID76	ACATGACGAC	MID107	TCGAGCTCTC	MID138	CTCGCACGCG
MID15	ATACGACGTA	MID46	TGACGTATGT	MID77	ACGACAGCTC	MID108	TCGCAGACAC	MID139	CTGCGTCACG
MID16	TCACGTACTA	MID47	TGTGAGTAGT	MID78	ACGTCTCATC	MID109	TCTGTCTCGC	MID140	CTGTGCGTCG
MID17	CGTCTAGTAC	MID48	ACAGTATATA	MID79	ACTCATCTAC	MID110	TGAGTGACGC	MID141	TAGCATACTG
MID18	TCTACGTAGC	MID49	ACGCGATCGA	MID80	ACTCGCGCAC	MID111	TGATGTGTAC	MID142	TATACATGTG
MID19	TGTACTACTC	MID50	ACTAGCAGTA	MID81	AGAGCGTCAC	MID112	TGCTATAGAC	MID143	TATCACTCAG
MID20	ACGACTACAG	MID51	AGCTCACGTA	MID82	AGCGACTAGC	MID113	TGCTCGCTAC	MID144	TATCTGATAG
MID21	CGTAGACTAG	MID52	AGTATACATA	MID83	AGTAGTGATC	MID114	ACGTGCAGCG	MID145	TCGTGACATG
MID22	TACGAGTATG	MID53	AGTCGAGAGA	MID84	AGTGACACAC	MID115	ACTCACAGAG	MID146	TCTGATCGAG
MID23	TACTCTCGTG	MID54	AGTGCTACGA	MID85	AGTGTATGTC	MID116	AGACTCAGCG	MID147	TGACATCTCG
MID24	TAGAGACGAG	MID55	CGATCGTATA	MID86	ATAGATAGAC	MID117	AGAGAGTGTG	MID148	TGAGCTAGAG
MID25	TCGTCGCTCG	MID56	CGCAGTACGA	MID87	ATATAGTCGC	MID118	AGCTATCGCG	MID149	TGATAGAGCG
MID26	ACATACGCGT	MID57	CGCGTATACA	MID88	ATCTACTGAC	MID119	AGTCTGACTG	MID150	TGCGTGTGCG
MID27	ACGCGAGTAT	MID58	CGTACAGTCA	MID89	CACGTAGATC	MID120	AGTGAGCTCG	MID151	TGCTAGTCAG
MID28	ACTACTATGT	MID59	CGTACTCAGA	MID90	CACGTGTCGC	MID121	ATAGCTCTCG	MID152	TGTATCACAG
MID29	ACTGTACAGT	MID60	CTACGCTCTA	MID91	CATACTCTAC	MID122	ATCACGTGCG	MID153	TGTGCGCGTG
MID30	AGACTATACT	MID61	CTATAGCGTA	MID92	CGACACTATC	MID123	ATCGTAGCAG		
MID31	AGCGTCGTCT	MID62	TACGTCATCA	MID93	CGAGACGCGC	MID124	ATCGTCTGTG		
