# Supplementary material for: The Sequencing Bead Array (SBA), a Next-Generation Digital Suspension Array
Source: PLoS One. 2013 Oct 7;8(10):e76696. doi: 10.1371/journal.pone.0076696 (PMC3792038; doi:10.1371/journal.pone.0076696)
Supplement: Table S2 — Metadata for library variability studies of six individually emulsion PCR constructed 10-plex HPV reporter libraries (L1-L6). The table contains Torrent Suite and Sphix-generated data from the sequencing runs performed on the interrogated libraries. Calls denote actual sequence read counts. Left columns contain sequence run information with reporter population and subcategories (defined in Table 1). Frequency denotes the fraction of the subcategories compared to the reporter population. Middle columns contain sequence read counts for reporter distribution, and frequency denotes the fractions of reporters as compared to called reporters. The right columns contain bead density estimates based on fluorescent quantification of single-stranded DNA in the libraries. Following estimates were made: i) an average of 800,000 oligonucleotides clustered on each bead, and ii) oligonucleotides have an average length of 114.8-bp, resulting in a single strand DNA molecular weight of 34943.76 g/mole (number of nucleotides x 303.7 + 79.0). Calculation of weight/bead = (34943.76/Avogadro’s constant) x 800,000. The table is summarized with average (AVG) and standard deviation (STDEV) values for all components. Furthermore maximum (MAXDEV) and minimum (MINDEV) values refer to the upper and lower deviation from the average value. (PDF) [file pone.0076696.s007.pdf]

		Reporter population	Polyclonality	Low quality	Uncalled reporters	Called reporters	HPV-16	HPV-18	HPV-33	HPV-35	HPV-39	HPV-45	HPV-52	HPV-56	HPV-58	HPV-59	Conc. [g/ul]	Density [beads/ul]
**L1**	**calls**	6.50E+05	2.33E+05	4.54E+04	2.14E+04	3.50E+05	2.62E+04	2.31E+04	2.83E+04	3.91E+04	3.39E+04	4.54E+04	2.87E+04	3.96E+04	4.20E+04	4.40E+04	2.85E-08	6.14E+05
	**frequency**		35.86%	6.98%	3.30%	53.86%	7.48%	6.59%	8.07%	11.16%	9.68%	12.96%	8.19%	11.31%	11.99%	12.56%		
**L2**	**calls**	7.34E+05	2.75E+05	6.88E+04	2.14E+04	3.68E+05	2.74E+04	2.41E+04	3.05E+04	3.95E+04	3.56E+04	4.75E+04	3.12E+04	4.28E+04	4.38E+04	4.58E+04	2.72E-08	5.85E+05
	**frequency**		37.54%	9.37%	2.92%	50.17%	7.44%	6.54%	8.29%	10.72%	9.66%	12.91%	8.47%	11.61%	11.90%	12.45%		
**L3**	**calls**	6.96E+05	2.50E+05	6.71E+04	2.09E+04	3.59E+05	2.62E+04	2.38E+04	2.97E+04	3.81E+04	3.46E+04	4.63E+04	3.14E+04	4.21E+04	4.27E+04	4.39E+04	3.13E-08	6.74E+05
	**frequency**		35.86%	9.63%	3.00%	51.50%	7.29%	6.64%	8.27%	10.62%	9.64%	12.90%	8.76%	11.73%	11.89%	12.25%		
**L4**	**calls**	7.55E+05	2.96E+05	7.09E+04	2.08E+04	3.67E+05	2.83E+04	2.66E+04	2.98E+04	3.81E+04	3.37E+04	4.72E+04	2.98E+04	4.38E+04	4.38E+04	4.63E+04	3.24E-08	6.98E+05
	**frequency**		39.22%	9.39%	2.75%	48.63%	7.70%	7.24%	8.10%	10.36%	9.18%	12.85%	8.12%	11.93%	11.92%	12.60%		
**L5**	**calls**	6.55E+05	2.29E+05	6.53E+04	2.07E+04	3.40E+05	2.52E+04	2.26E+04	2.85E+04	3.64E+04	3.31E+04	4.33E+04	2.77E+04	3.91E+04	4.09E+04	4.30E+04	2.43E-08	5.23E+05
	**frequency**		34.97%	9.97%	3.16%	51.90%	7.43%	6.66%	8.38%	10.72%	9.73%	12.75%	8.15%	11.52%	12.03%	12.65%		
**L6**	**calls**	6.34E+05	1.76E+05	8.09E+04	2.09E+04	3.56E+05	2.64E+04	2.36E+04	2.99E+04	3.86E+04	3.57E+04	4.42E+04	2.95E+04	4.17E+04	4.25E+04	4.41E+04	2.15E-08	4.62E+05
	**frequency**		27.74%	12.77%	3.29%	56.20%	7.41%	6.63%	8.40%	10.84%	10.03%	12.40%	8.29%	11.71%	11.92%	12.37%		
	**AVG calls**	6.87E+05	2.43E+05	6.64E+04	2.10E+04	3.57E+05	2.66E+04	2.40E+04	2.94E+04	3.83E+04	3.44E+04	4.56E+04	2.97E+04	4.15E+04	4.26E+04	4.45E+04	2.75E-08	5.93E+05
	**AVG frequency**		35.20%	9.68%	3.07%	52.04%	7.46%	6.72%	8.25%	10.74%	9.65%	12.79%	8.33%	11.64%	11.94%	12.48%		
	**STDEV calls**	4.93E+04	4.18E+04	1.17E+04	3.29E+02	1.07E+04	1.07E+03	1.38E+03	8.84E+02	1.07E+03	1.07E+03	1.68E+03	1.43E+03	1.81E+03	1.12E+03	1.27E+03	4.16E-09	8.95E+04
	**STDEV frequency**		3.95%	1.85%	0.22%	2.68%	0.13%	0.26%	0.14%	0.26%	0.27%	0.21%	0.25%	0.21%	0.06%	0.15%		
	**MAXDEV calls**	6.79E+04	5.30E+04	1.45E+04	4.20E+02	1.14E+04	1.67E+03	2.60E+03	1.10E+03	1.18E+03	1.31E+03	1.89E+03	1.69E+03	2.30E+03	1.20E+03	1.77E+03	4.89E-09	1.05E+05
	**MAXDEV frequency**		4.02%	3.08%	0.23%	4.15%	0.24%	0.52%	0.14%	0.43%	0.38%	0.16%	0.43%	0.29%	0.08%	0.17%		
	**MINDEV calls**	5.36E+04	6.74E+04	2.10E+04	3.11E+02	1.69E+04	1.38E+03	1.35E+03	1.17E+03	1.87E+03	1.36E+03	2.32E+03	2.03E+03	2.38E+03	1.73E+03	1.52E+03	6.05E-09	1.30E+05
	**MINDEV frequency**		7.46%	2.71%	0.32%	3.41%	0.17%	0.18%	0.18%	0.37%	0.47%	0.40%	0.21%	0.32%	0.05%	0.23%		
