# Supplementary material for: The Sequencing Bead Array (SBA), a Next-Generation Digital Suspension Array
Source: PLoS One. 2013 Oct 7;8(10):e76696. doi: 10.1371/journal.pone.0076696 (PMC3792038; doi:10.1371/journal.pone.0076696)
Supplement: Table S3 — Metadata for background noise negative (water) selections in six replicates (X1-X6). The table contains Torrent Suite and Sphix-generated data from the sequencing runs performed on the interrogated libraries. Calls denote actual sequence read counts. Left columns contain sequence run information with reporter population and subcategories (defined in Table 1). Frequency denotes the fraction of the subcategories compared to the reporter population. Right columns contain sequence read counts for reporter distribution, and frequency denotes the fractions of reporters as compared to called reporters. The table is summarized with average (AVG) and standard deviation (STDEV) values for all components. Furthermore maximum (MAXDEV) and minimum (MINDEV) values refer to the upper and lower deviation from the average value. The last row of the table details the threshold line based on the statistical three-sigma rule (mean + 3x standard deviation) for the different reporters. The highest value (reporter-56) was chosen for a conservative threshold line, which equals ~1261.5 reads. (PDF) [file pone.0076696.s008.pdf]

		Reporter population	Polyclonality	Low quality	Uncalled reporters	Called reporters	HPV-16	HPV-18	HPV-33	HPV-35	HPV-39	HPV-45	HPV-52	HPV-56	HPV-58	HPV-59
**X1**	**calls**	1.52E+04	2.96E+03	8.79E+03	1.84E+02	3.30E+03	1.92E+02	1.97E+02	3.35E+02	3.33E+02	3.00E+02	4.21E+02	3.12E+02	5.03E+02	4.03E+02	3.02E+02
	**frequency**		19.44%	57.70%	1.21%	21.65%	5.82%	5.97%	10.16%	10.10%	9.10%	12.77%	9.46%	15.25%	12.22%	9.16%
**X2**	**calls**	2.16E+04	5.78E+03	9.84E+03	2.98E+02	5.66E+03	3.16E+02	3.93E+02	6.17E+02	6.20E+02	5.26E+02	7.37E+02	4.92E+02	8.11E+02	6.61E+02	4.84E+02
	**frequency**		26.79%	45.60%	1.38%	26.23%	5.59%	6.95%	10.91%	10.96%	9.30%	13.03%	8.70%	14.34%	11.68%	8.56%
**X3**	**calls**	1.05E+04	1.11E+03	8.12E+03	1.25E+02	1.17E+03	6.50E+01	8.10E+01	1.27E+02	1.22E+02	1.01E+02	1.38E+02	1.13E+02	1.93E+02	1.40E+02	8.90E+01
	**frequency**		10.53%	77.18%	1.19%	11.11%	5.56%	6.93%	10.86%	10.44%	8.64%	11.80%	9.67%	16.51%	11.98%	7.61%
**X4**	**calls**	1.74E+04	4.56E+03	7.53E+03	3.90E+02	4.90E+03	3.07E+02	3.09E+02	4.89E+02	5.39E+02	4.13E+02	6.69E+02	4.45E+02	7.10E+02	5.64E+02	4.59E+02
	**frequency**		26.22%	43.33%	2.24%	28.21%	6.26%	6.30%	9.97%	10.99%	8.42%	13.64%	9.07%	14.48%	11.50%	9.36%
**X5**	**calls**	1.28E+04	3.00E+03	6.65E+03	1.46E+02	3.03E+03	1.49E+02	2.13E+02	3.13E+02	3.27E+02	3.28E+02	3.58E+02	3.55E+02	4.16E+02	3.12E+02	2.61E+02
	**frequency**		23.35%	51.87%	1.14%	23.64%	4.91%	7.03%	10.32%	10.78%	10.82%	11.81%	11.71%	13.72%	10.29%	8.61%
**X6**	**calls**	1.04E+04	1.20E+03	7.78E+03	1.89E+02	1.18E+03	6.70E+01	6.90E+01	1.24E+02	1.23E+02	1.31E+02	1.35E+02	1.45E+02	1.57E+02	1.21E+02	1.12E+02
	**frequency**		11.58%	75.16%	1.83%	11.43%	5.66%	5.83%	10.47%	10.39%	11.06%	11.40%	12.25%	13.26%	10.22%	9.46%
	**AVG calls**	1.46E+04	3.10E+03	8.12E+03	2.22E+02	3.21E+03	1.83E+02	2.10E+02	3.34E+02	3.44E+02	3.00E+02	4.10E+02	3.10E+02	4.65E+02	3.67E+02	2.85E+02
	**AVG frequency**		19.65%	58.47%	1.50%	20.38%	5.63%	6.50%	10.45%	10.61%	9.56%	12.41%	10.14%	14.59%	11.32%	8.79%
	**STDEV calls**	4.35E+03	1.84E+03	1.10E+03	1.02E+02	1.85E+03	1.11E+02	1.26E+02	1.96E+02	2.06E+02	1.63E+02	2.56E+02	1.55E+02	2.65E+02	2.20E+02	1.67E+02
	**STDEV frequency**		7.16%	14.61%	0.44%	7.40%	0.44%	0.53%	0.38%	0.36%	1.12%	0.87%	1.47%	1.16%	0.86%	0.69%
	**MAXDEV calls**	6.92E+03	2.68E+03	1.72E+03	1.68E+02	2.45E+03	1.33E+02	1.83E+02	2.83E+02	2.76E+02	2.26E+02	3.27E+02	1.82E+02	3.46E+02	2.94E+02	2.00E+02
	**MAXDEV frequency**		7.14%	18.70%	0.75%	7.83%	0.63%	0.52%	0.46%	0.38%	1.51%	1.23%	2.10%	1.92%	0.90%	0.67%
	**MINDEV calls**	4.29E+03	1.99E+03	1.47E+03	9.70E+01	2.04E+03	1.18E+02	1.41E+02	2.10E+02	2.22E+02	1.99E+02	2.75E+02	1.97E+02	3.08E+02	2.46E+02	1.96E+02
	**MINDEV frequency**		9.12%	15.15%	0.36%	9.27%	0.72%	0.67%	0.48%	0.51%	1.13%	1.01%	1.45%	1.33%	1.10%	1.18%
	**THRESHOLD**						5.16E+02	5.90E+02	9.21E+02	9.63E+02	7.89E+02	1.18E+03	7.74E+02	1.26E+03	1.03E+03	7.85E+02
