# Supplementary material for: The Sequencing Bead Array (SBA), a Next-Generation Digital Suspension Array
Source: PLoS One. 2013 Oct 7;8(10):e76696. doi: 10.1371/journal.pone.0076696 (PMC3792038; doi:10.1371/journal.pone.0076696)
Supplement: Table S5 — Metadata for Sequencing Bead Array (SBA) assay screens of 20 cervical tumor samples. Cervical tumor samples (extracted genomic DNA) derived from different females (OM-1078, OM-1272, OM-1299, OM-1301, OM-1452, OM-1464, OM-1530, OM-1569, OM-1668, OM-1741, OM-1751, OM-1848, OM-1854, OM-1967, OM-1980, OM-2006, OM-2059, OM-2215, OM-2257, and OM-2258). The table contains Torrent Suite and Sphix-generated data from the sequencing runs performed on the interrogated libraries. Calls denote actual sequence read counts. Left columns contain sequence run information with reporter population and subcategories (defined in Table 1). Frequency denotes the fraction of the subcategories compared to the reporter population. Right columns contain sequence read counts for reporter distribution, and frequency denotes the fractions of reporters as compared to called reporters. (PDF) [file pone.0076696.s010.pdf]

		Reporter population	Polyclonality	Low quality	Uncalled reporters	Called reporters	HPV-16	HPV-18	HPV-33	HPV-35	HPV-39	HPV-45	HPV-52	HPV-56	HPV-58	HPV-59
**OM-1078**	**calls**	1.09E+05	4.86E+04	4.90E+04	1.24E+03	9.98E+03	7.67E+03	1.03E+02	2.21E+02	2.24E+02	1.78E+02	2.61E+02	2.88E+02	4.52E+02	3.80E+02	2.01E+02
	**frequency**		44.68%	45.02%	1.14%	9.17%	76.87%	1.03%	2.21%	2.24%	1.78%	2.62%	2.89%	4.53%	3.81%	2.01%
**OM-1272**	**calls**	1.16E+05	5.34E+04	4.79E+04	1.53E+03	1.31E+04	8.85E+03	2.64E+02	4.60E+02	4.62E+02	3.87E+02	4.81E+02	4.81E+02	7.33E+02	5.85E+02	4.11E+02
	**frequency**		46.03%	41.34%	1.32%	11.31%	67.48%	2.01%	3.51%	3.52%	2.95%	3.67%	3.67%	5.59%	4.46%	3.13%
**OM-1299**	**calls**	3.83E+04	8.24E+03	2.67E+04	3.28E+02	2.98E+03	2.44E+02	1.99E+02	2.88E+02	3.45E+02	2.58E+02	2.75E+02	2.96E+02	5.55E+02	3.00E+02	2.19E+02
	**frequency**		21.52%	69.83%	0.86%	7.79%	8.19%	6.68%	9.67%	11.58%	8.66%	9.23%	9.94%	18.63%	10.07%	7.35%
**OM-1301**	**calls**	4.84E+04	1.89E+04	1.61E+04	7.19E+02	1.27E+04	7.01E+03	4.22E+02	6.27E+02	6.00E+02	5.46E+02	7.97E+02	6.67E+02	8.05E+02	6.57E+02	5.65E+02
	**frequency**		39.09%	33.19%	1.49%	26.23%	55.21%	3.32%	4.94%	4.73%	4.30%	6.28%	5.25%	6.34%	5.18%	4.45%
**OM-1452**	**calls**	9.96E+04	3.96E+04	4.30E+04	1.07E+03	1.59E+04	3.52E+02	1.10E+04	4.50E+02	5.12E+02	5.55E+02	5.92E+02	3.84E+02	7.19E+02	7.98E+02	5.88E+02
	**frequency**		39.77%	43.19%	1.07%	15.98%	2.21%	68.91%	2.83%	3.22%	3.49%	3.72%	2.41%	4.52%	5.01%	3.69%
**OM-1464**	**calls**	6.03E+04	2.08E+04	3.45E+04	4.38E+02	4.48E+03	3.85E+03	2.30E+01	5.90E+01	5.10E+01	3.90E+01	7.00E+01	6.80E+01	1.17E+02	1.22E+02	7.80E+01
	**frequency**		34.56%	57.28%	0.73%	7.43%	86.00%	0.51%	1.32%	1.14%	0.87%	1.56%	1.52%	2.61%	2.72%	1.74%
**OM-1530**	**calls**	7.43E+04	4.05E+04	1.95E+04	1.42E+03	1.29E+04	1.14E+04	8.90E+01	1.55E+02	1.79E+02	8.40E+01	1.79E+02	1.30E+02	2.87E+02	2.41E+02	1.79E+02
	**frequency**		54.45%	26.27%	1.91%	17.37%	88.21%	0.69%	1.20%	1.39%	0.65%	1.39%	1.01%	2.22%	1.87%	1.39%
**OM-1569**	**calls**	4.92E+04	1.79E+04	1.60E+04	6.13E+02	1.46E+04	1.23E+02	1.58E+02	2.25E+02	1.98E+02	2.09E+02	3.42E+02	2.53E+02	3.59E+02	2.69E+02	1.25E+04
	**frequency**		36.47%	32.59%	1.25%	29.69%	0.84%	1.08%	1.54%	1.36%	1.43%	2.34%	1.73%	2.46%	1.84%	85.38%
**OM-1668**	**calls**	4.15E+04	1.50E+04	1.26E+04	7.43E+02	1.31E+04	3.21E+02	4.15E+02	5.58E+02	5.85E+02	5.29E+02	7.95E+02	7.16E+02	8.55E+02	6.49E+02	7.70E+03
	**frequency**		36.23%	30.36%	1.79%	31.62%	2.45%	3.16%	4.25%	4.46%	4.03%	6.06%	5.45%	6.51%	4.94%	58.69%
**OM-1741**	**calls**	6.63E+04	3.26E+04	2.51E+04	7.25E+02	7.83E+03	1.21E+02	6.42E+03	1.18E+02	1.24E+02	2.09E+02	1.65E+02	1.09E+02	1.73E+02	2.77E+02	1.20E+02
	**frequency**		49.23%	37.86%	1.09%	11.82%	1.54%	81.92%	1.51%	1.58%	2.67%	2.11%	1.39%	2.21%	3.54%	1.53%
**OM-1751**	**calls**	2.02E+04	7.57E+03	7.54E+03	2.88E+02	4.77E+03	3.35E+03	8.50E+01	1.20E+02	1.47E+02	1.33E+02	1.96E+02	1.84E+02	2.37E+02	1.50E+02	1.67E+02
	**frequency**		37.56%	37.38%	1.43%	23.63%	70.22%	1.78%	2.52%	3.08%	2.79%	4.11%	3.86%	4.97%	3.15%	3.50%
**OM-1848**	**calls**	4.50E+04	1.68E+04	1.45E+04	4.37E+02	1.33E+04	1.45E+02	1.46E+02	2.08E+02	1.91E+02	2.25E+02	1.13E+04	2.80E+02	3.13E+02	2.64E+02	2.38E+02
	**frequency**		37.25%	32.25%	0.97%	29.53%	1.09%	1.10%	1.56%	1.44%	1.69%	84.88%	2.11%	2.35%	1.99%	1.79%
**OM-1854**	**calls**	3.98E+04	1.23E+04	1.79E+04	5.62E+02	9.12E+03	1.47E+02	6.86E+03	2.01E+02	2.49E+02	2.15E+02	3.20E+02	2.74E+02	3.51E+02	2.68E+02	2.31E+02
	**frequency**		30.80%	44.89%	1.41%	22.91%	1.61%	75.26%	2.20%	2.73%	2.36%	3.51%	3.00%	3.85%	2.94%	2.53%
**OM-1967**	**calls**	1.06E+05	4.85E+04	4.43E+04	1.44E+03	1.20E+04	9.39E+03	2.76E+02	2.43E+02	2.84E+02	2.26E+02	2.70E+02	2.33E+02	4.35E+02	3.60E+02	3.04E+02
	**frequency**		45.63%	41.71%	1.36%	11.31%	78.12%	2.30%	2.02%	2.36%	1.88%	2.25%	1.94%	3.62%	2.99%	2.53%
**OM-1980**	**calls**	8.48E+04	4.16E+04	2.50E+04	9.64E+02	1.72E+04	1.90E+02	1.23E+04	3.90E+02	8.19E+02	4.65E+02	7.00E+02	6.34E+02	6.53E+02	5.09E+02	6.18E+02
	**frequency**		49.08%	29.45%	1.14%	20.34%	1.10%	71.13%	2.26%	4.75%	2.70%	4.06%	3.68%	3.79%	2.95%	3.58%
**OM-2006**	**calls**	7.03E+04	2.85E+04	3.56E+04	4.78E+02	5.68E+03	4.08E+03	9.30E+01	1.21E+02	2.35E+02	1.31E+02	1.68E+02	2.19E+02	1.97E+02	1.96E+02	2.44E+02
	**frequency**		40.52%	50.72%	0.68%	8.08%	71.76%	1.64%	2.13%	4.14%	2.31%	2.96%	3.86%	3.47%	3.45%	4.30%
**OM-2059**	**calls**	6.09E+04	1.96E+04	3.62E+04	4.17E+02	4.62E+03	3.16E+03	8.70E+01	1.38E+02	2.23E+02	1.22E+02	1.53E+02	1.76E+02	1.76E+02	1.62E+02	2.18E+02
	**frequency**		32.21%	59.52%	0.69%	7.59%	68.49%	1.88%	2.99%	4.83%	2.64%	3.31%	3.81%	3.81%	3.51%	4.72%
**OM-2215**	**calls**	1.21E+05	6.43E+04	3.50E+04	1.28E+03	2.03E+04	4.15E+03	1.11E+04	4.55E+02	7.86E+02	5.48E+02	6.11E+02	6.60E+02	6.81E+02	5.61E+02	7.12E+02
	**frequency**		53.23%	28.94%	1.06%	16.77%	20.49%	54.75%	2.25%	3.88%	2.71%	3.02%	3.26%	3.36%	2.77%	3.52%
**OM-2257**	**calls**	7.55E+04	4.18E+04	2.37E+04	8.90E+02	9.18E+03	6.42E+03	1.75E+02	2.34E+02	4.01E+02	2.46E+02	3.23E+02	3.65E+02	3.68E+02	2.79E+02	3.75E+02
	**frequency**		55.34%	31.32%	1.18%	12.16%	69.87%	1.91%	2.55%	4.37%	2.68%	3.52%	3.98%	4.01%	3.04%	4.08%
**OM-2258**	**calls**	1.53E+05	5.95E+04	6.51E+04	1.35E+03	2.68E+04	8.48E+03	3.14E+02	6.12E+02	5.93E+02	5.85E+02	1.35E+04	5.32E+02	8.67E+02	8.92E+02	4.61E+02
	**frequency**		38.96%	42.59%	0.88%	17.57%	31.62%	1.17%	2.28%	2.21%	2.18%	50.28%	1.98%	3.23%	3.32%	1.72%
